# SpatialCOC: an integrative framework for spatial continuous mapping and cross-omics correction in spatial multi-omics data

**DOI:** 10.1038/s41467-026-71882-2

**Published:** 2026-04-16

**Authors:** Mingxuan Li, Peisen Sun, Yisi Luo, Guancheng Zhou, Xiaofei Yang, Deyu Meng, Kai Ye

**Affiliations:** 1 MOE Key Lab for Intelligent Networks & Networks Security, Faculty of Electronic and Information Engineering, Xi’an Jiaotong University, Xi’an, China; 2 School of Automation Science and Engineering, Faculty of Electronic and Information Engineering, Xi’an Jiaotong University, Xi’an, China; 3 School of Mathematics and Statistics, Xi’an Jiaotong University, Xi’an, China; 4 School of Computer Science and Technology, Faculty of Electronic and Information Engineering, Xi’an Jiaotong University, Xi’an, China; 5 Macao Institute of Systems Engineering, Macau University of Science and Technology, Macao, China; 6 Department of Gynecology and Obstetrics, Center for Mathematical Medical, The First Affiliated Hospital of Xi’an Jiaotong University, Xi’an, China; 7 Genome Institute, The First Affiliated Hospital of Xi’an Jiaotong University, Xi’an, China; 8 Faculty of Science, Leiden University, Leiden, The Netherlands

**Keywords:** Data integration, Machine learning, Computational models, Software

## Abstract

Integrating spatial multi-omics data presents significant challenges, particularly in uncovering the spatial patterns of cells and deciphering the real regulatory mechanisms among various omics. These insights are critical for harnessing the full potential of each modality while minimizing the impact of biotechnological biases that will lead to unstable results. Here, we introduce SpatialCOC, a framework that treats spatial information as prior knowledge to learn omics-specific spatial distributions, then discovering nonlinear correlations among modalities. The effectiveness and robustness of SpatialCOC are validated using real-world datasets, encompassing diverse tissue sections analyzed with multiple experimental techniques. Compared to existing methods, SpatialCOC excels in identifying region-specific continuous spatial domains and maintains batch-consistency across trajectory inferences. By providing a novel perspective on the interplay between spatial information and multi-omics modalities, SpatialCOC offers a flexible approach that can accommodate modality data of arbitrary dimensions.

## Abstract (web summary)

Analyzing spatial patterns and regulatory mechanisms in spatial multi-omics data remains challenging. Here, the authors present SpatialCOC, a deep learning framework that deciphers cellular spatial organization and cross-omics relationship.

## Introduction

Spatial multi-omics integrates multiple omics modalities, such as genomics, epigenomics, transcriptomics, and proteomics within shared spatial coordinates, providing comprehensive insights into biological processes with spatial dimension. Technologies such as Slide-DNA-seq^1^, Spatial ATAC-RNA-seq^2^, SPOTS^3^, and MIP-seq^4^ enable precise localization of cells within tissues and quantitative measurement of various omics^5^. These advances have unlocked new opportunities for understanding cellular functions and disease mechanisms^4^. For instance, spatial multi-omics reveals genetic characteristics, cell types, and their interactions within a spatial context, deepening our understanding of organ structure and function^6^. Additionally, these technologies facilitate the exploration of metabolite crosstalk and competition within the tumor microenvironment (TME)^7^, offering valuable insights into cancer pathogenesis. Despite its potential, integrating multiple omics modalities within a spatial context remains a significant challenge. The integration process must account for spatial information and the complex nonlinear interactions among omics modalities, both of which are critical for uncovering novel biomedical insights.

Existing integrative methods employ a “bottom-up” strategy: modeling spatial information and each omics data separately, followed by a systematic, bottom-up fusion process. They usually rely on structured representations—such as neighborhood graphs—to uniformly characterize multiple modalities (e.g., SpatialGlue^8^, COSMOS^9^, SpaTrio^10^, STAGATE^11^, SpaGCN^12^, and GraphST^13^), and subsequently integrate them by “weighted summation” or by extracting shared latent embeddings (e.g., Seurat WNN^14^, MultiVI^15^, MultiMAP^16^, and GLUE^17^). The “bottom-up” integrative approach provides a unified paradigm for spatial multi-omics integration, offering a classical and effective framework for constructing unified representations across modalities. Nonetheless, this strategy still suffers from inherent limitations. The structured representations primarily capture local neighborhood information and are noise-sensitive^18^, making it difficult to construct a unified space that fairly reflects the characteristics of each modality. Moreover, weighted summation is inherently linear and competitive, resulting in suboptimal integration. This approach may not only fail to adequately capture complex inter-modality relationships but also aggregate noise from each modality. Analyses of the mouse thymus dataset below further suggest that this may compromise the reliability of downstream analyses.

To overcome these limitations, we propose SpatialCOC, a “top-down” integrative framework that explicitly incorporates two key characteristics of spatial multi-omics data: spatial colocalization and complementarity among omics layers. SpatialCOC treats spatial information as top-level prior knowledge to learn omics-specific spatial distribution functions, and subsequently captures bottom-level nonlinear inter-modal relationships through a “correction” approach. This process enables the capture of diverse spatial patterns, ensures spatial smoothness, and removes modality-specific noise. To validate the effectiveness and robustness of SpatialCOC, we utilized augmented datasets of human lymph node tissue (HLN-augmented datasets), which were generated from real-based data by simulating multiple spatial patterns and introducing various noise types. Subsequently, we applied SpatialCOC to tissue sections from the mouse brain, thymus, and spleen, thereby systematically confirming the accuracy and reliability of downstream analyses. Compared to seven state-of-the-art methods—covering spatial multi-omics (SpatialGlue^8^ and COSMOS^9^), single-cell multi-omics (Seurat WNN^14^, MultiVI^15^, and MultiMAP^16^), and spatial transcriptomics (STAGATE^11^ and SpaGCN^12^)—SpatialCOC demonstrated superior performance across multiple downstream analytical tasks. It accurately identified tissue structures while maintaining spatial smoothness in mouse brain and spleen datasets. Additionally, SpatialCOC exhibited strong consistency in trajectory inference across multiple slices of the mouse thymus.

## Results

### SpatialCOC framework

**Fig. 1:**
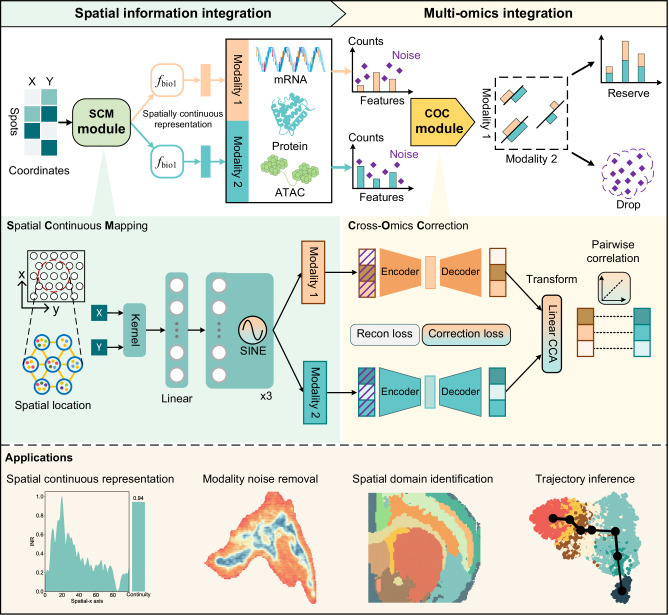
Overview of spatial multi-omics integration using SpatialCOC. SpatialCOC treats spatial information as a prior distribution for each omics modality and employs the Spatial Continuous Mapping (SCM) module to learn its continuous spatial representation. By leveraging sinusoidal activation functions and kernel functions, the SCM module captures modality-specific spatial patterns while ensuring spatial smoothness, thereby implicitly integrating spatial information with omics data. Furthermore, SpatialCOC learns nonlinear inter-omics relationships through the Cross-Omics Correction (COC) module. This module uses an autoencoder that builds upon the reconstruction loss by introducing a correction loss, paired with a linear CCA layer, to both capture nonlinear correlations across omics modalities and ensure proper alignment between them. The output of SpatialCOC can be used for spatial domain identification, modality noise removal, and trajectory inference. The mRNA icon is by Servier (https://smart.servier.com/) and is licensed under CC-BY 3.0 Unported (https://creativecommons.org/licenses/by/3.0/). The protein icon is by DBCLS and is licensed under CC-BY 4.0 Unported (https://creativecommons.org/licenses/by/4.0/). The aspect ratio, orientation, and color scheme of both icons have been adjusted to fit the canvas size.

To address the key challenges of integrating spatial multi-omics—the diversity in the spatial distribution of omics data and the nonlinearity of inter-omics relationships—our framework was designed with the following properties: (1) Integration of spatial information. Instead of treating spatial information as a parallel modality alongside omics, the framework must leverage its sharable nature to enhance integration; (2) Integration among omics modalities. A “correction” approach should be employed to avoid the simple linear integration, such as “weighted summation”, which fails to capture nonlinear interaction mechanisms and propagates modality-specific noise. With these principles in mind, we developed SpatialCOC, a unified computational framework to integrate spatial multi-omics data (Fig. 1).

Spatial continuous mapping (SCM) module for spatial information integration. To capture diverse spatial patterns across different omics, the SCM module first learns a continuous spatial representation using mathematical function mappings from spatial coordinates to multi-omics. Specifically, spatial coordinates serve as inputs to a neural network employing three sine periodic activation functions to reconstruct each omics modality. This reconstruction process builds upon implicit neural representations (INRs) with periodic activations^19,20^, and further enhances continuous representation capability through kernel functions. Leveraging the powerful representational capacity of this paradigm, the SCM module addresses three significant heterogeneities across different omics data, including differences in biological implications, dimensionalities, and sparsity levels. We provided the underlying mathematical principles of this module, as well as systematic testing of the sinusoidal activation function and kernel functions (Supplementary Note 1, Supplementary Figs. 1a, b and 2a, b). By treating spatial information as prior knowledge rather than a standalone modality, SpatialCOC learns continuous spatial patterns of omics modalities. This design accommodates diverse combinations of modalities across varying dimensions, from raw features to reduced-dimension embeddings (Supplementary Fig. 3a).

Cross-omics correction (COC) module for multi-omics integration. To capture the nonlinear correlations among omics modalities while eliminating modality-specific noise, the COC module incorporates a dual-loss optimization strategy, an approach inspired by canonical correlation analysis (CCA), particularly its deep learning variant, DCCAE^21^. The COC module processes spatially continuous omics reconstructions and jointly optimizes Correction Loss and Reconstruction Loss during training. Subsequently, a linear CCA layer transformation is performed to produce well-aligned multimodal representations. This design captures deep nonlinear relationships among omics modalities while preserving their individual characteristics, and facilitates pairwise interaction of the integrated embeddings. By identifying and removing cross-modally irrelevant noise^22^, SpatialCOC integrates omics data in a “correction” manner rather than relying on a “weighted summation” approach that may amplify noise.

SpatialCOC pioneers the treatment spatial information as prior knowledge to learn omics-specific spatial distributions, and discovers nonlinear correlations among them. By capturing the globally continuous spatial distribution of omics data, the SCM module enables the COC module to better model nonlinear correlation across both expression and spatial contexts. The necessity of each component within the SpatialCOC framework and its loss function design was validated through ablation experiments conducted on HLN-augmented datasets (Supplementary Fig. 3b and Supplementary Note 2). The logical coherence between modules was validated through comparative experiment conducted on the mouse brain dataset (Supplementary Fig. 4a, b).

### Validating the effectiveness of the SCM and COC modules on the human lymph node (HLN)-augmented datasets

**Fig. 2:**
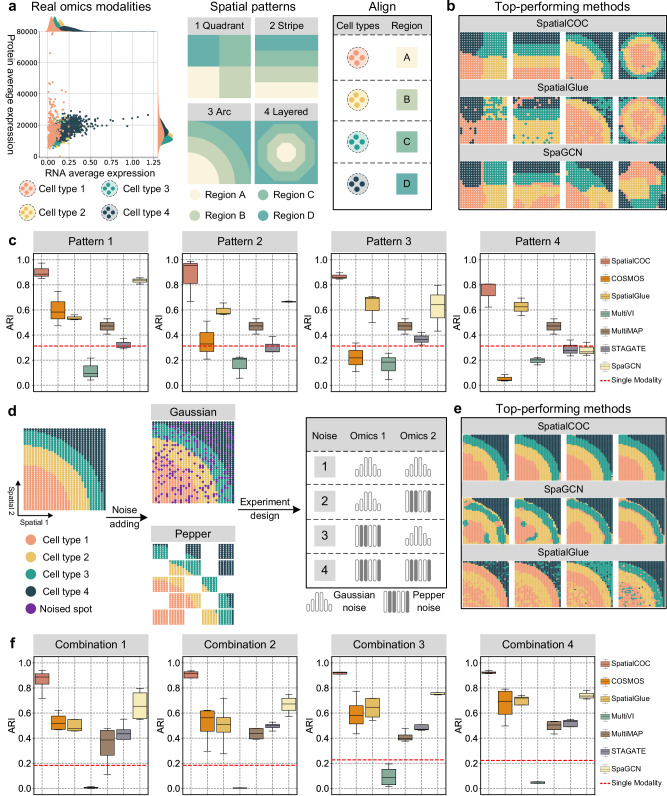
Design and benchmarking of experiments on the human lymph node (HLN)-augmented datasets. **a** Simulation of four spatial distribution patterns. Real omics expressions and labels from the Human Lymph Node dataset, comprising four cell types, were randomly sampled (300 spots per cell type, 1200 spots in total) and divided into three replicate groups. Subsequently, four spatial patterns of increasing complexity were simulated: quadrant, stripe, arc, and layered, each comprising four regions designated for specific cell types with assigned spatial coordinates. **b** Spatial domain identification by top-performing methods across four spatial patterns: SpatialCOC, SpatialGlue, and SpaGCN (visualized for the first replicate group). **c** Box plots of clustering accuracy across spatial patterns for seven methods. Adjusted rand index (ARI) was used to quantify performance under four spatial patterns (*n* = 3 replicate groups for each method). In the boxplot, the center line, box limits, and whiskers denote the median, upper, and lower quartiles, and 1.5× interquartile range, respectively. The red dashed line denotes the best median ARI achieved by mono-omics clustering and serves as the reference baseline. **d** Noise simulation and experimental design. Two types of noise were introduced into the arc spatial pattern data: Gaussian noise was added to simulate realistic experimental measurement errors, while pepper noise was used to model dropout events. All pairwise combinations of these noise types were designed across three intensity levels, with a no-noise condition included as the control. **e** Spatial domain identification by top-performing methods across four noise combinations: SpatialCOC, SpaGCN, and SpatialGlue (visualized for the second intensity gradient). **f** Box plots of clustering accuracy across noise combinations for seven methods. The performance of each method was quantified using the ARI under three levels of added noise and a no-noise baseline condition (*n* = 4 combinations). The boxplot representation and the red dashed line follow the same formats as in (**c**). Source data are provided as a Source data file.

To evaluate the rationality and effectiveness of the proposed SCM and COC modules, we constructed two distinct augmented datasets derived from real human lymph node tissue. By incorporating diverse spatial patterns (Fig. 2a–c) and graded noise (Fig. 2d–f), we established a more realistic and challenging benchmark than simulator-based alternatives.

- Dataset 1: spatial patterns of varying complexity. This dataset simulates different spatial structure patterns of cells, which is crucial for the functions of organism tissues^23^. Inspired by the work of Yang, P. et al.^10^, we designed and generated four different spatial patterns: quadrant, stripe, arc, and layered. Authentic omics data from the Human Lymph Node dataset^24^ were spatially allocated based on their true labels, providing a robust foundation for testing (Fig. 2a).
- Dataset 2: different noise levels and combinations. This dataset accounts for random errors commonly introduced by spatial multi-omics technologies, such as tissue degradation, reduced RNA molecule availability^25^, and “dropout” events^26^. Gaussian noise and pepper noise were applied to simulate varying levels and combinations, mimicking the impact of experimental variability on data integrity (Fig. 2d). Details of the HLN-augmented datasets generation process are provided in the “Methods” section.

Evaluation of the SCM module’s effectiveness. Using dataset 1, we evaluated the SCM module’s ability to simultaneously capture diverse spatial patterns while maintaining spatial smoothness. Clustering tasks were constructed across multiple spatial patterns, with performance evaluated using three metrics: adjusted rand index (ARI), adjusted mutual information (AMI), and normalized mutual information (NMI). SpatialCOC outperforms other methods in both accurately and continuously identifying spatial domains in all patterns. SpatialCOC, SpatialGlue, and SpaGCN ranked as the top-performing methods overall (Fig. 2b). Notably, SpatialCOC excelled in complex spatial patterns (such as layered pattern), while STAGATE and SpaGCN underperformed, achieving average performance even below those of mono-omics (Fig. 2c). Although SpaGCN generated relatively smooth categorizations, it failed to capture global spatial patterns consistently. This limitation highlights the challenges faced by structured approaches such as graph neighborhoods, which rely heavily on local neighborhood information. In contrast, SpatialCOC’s SCM module uses spatial information as prior knowledge for omics modalities, enabling it to flexibly explore and reconstruct diverse global spatial patterns.

Evaluation of the COC module’s effectiveness. Using dataset 2, we evaluated the COC module’s capacity to capture the nonlinear correlations among omics. Clustering performance was assessed using the same metrics (ARI, AMI, and NMI). Our results demonstrated that SpatialCOC consistently outperformed other methods in handling various noise levels and combinations. For instance, methods like COSMOS and SpatialGlue exhibited significant performance fluctuation across different noise combinations (Fig. 2e), while MultiVI failed to extract effective information when adding Gaussian noise to modality 1, with ARIs of 0.05 and 0.08 on average (Fig. 2f). In contrast, SpatialCOC demonstrated robustness, effectively distinguishing modality-specific signals even under high noise conditions. This robustness is attributed to the COC module’s “correction”-based approach, which removes noise irrelevant to the correlations among modalities^22^. By comparison, methods relying on “weighted summation” for integration are only able to reduce the weights of noisy modalities^14^, failing to address situations where both modalities contained noise.

Through benchmarking on two augmented datasets, we demonstrated that SpatialCOC’s SCM and COC modules can implicitly integrate spatial information and capture the complex nonlinear correlations across omics modalities. Complete results, including all technical replicates, are provided in Supplementary Figs. 5a–e, 6a–c and Supplementary Figs. 7, 8. Detailed procedures for all compared methods are provided in Supplementary Note 3. The detailed calculation process of the evaluation metrics can be found in Supplementary Note 4.

### Deciphering region-specific continuous spatial domains

Capturing diverse spatial patterns while maintaining appropriate spatial smoothness is essential for the effective integration of spatial information. To evaluate SpatialCOC’s capabilities, we analyzed two real datasets with distinct spatial characteristics:

- Mouse brain dataset^2^: the mouse brain—comprising gray matter (cerebral cortex and nuclei), fiber tracts, and ventricular systems—exhibits strong spatial patterns. This dataset captures distinct structural regions such as the layered isocortex (L1–L6) and anterior cingulate area (aca) within the cerebral cortex, as well as central nuclei like the caudoputamen (cp) and lateral septal nucleus (ls), alongside fiber tracts such as the genu of the corpus callosum (ccg)^27^. The reference for mouse brain ATAC-RNA integration was sourced from a P56 mouse brain coronal section provided by Allen Mouse Brain Atlas^28^ (see example in Supplementary Fig. 9a);
- Mouse spleen dataset^3^: this dataset includes regions with varying degrees of smoothness, including the vesicular structures formed by germinal centers (GCs) surrounded by the marginal zones (MZs), alongside the smoother macrophage-dominated areas of the red pulp/stroma regions^3^. This structural heterogeneity requires region-specific continuous spatial domain identification. By applying SpatialCOC to these two distinct datasets, we evaluated its capabilities to effectively capture diverse spatial patterns and maintain region-specific spatial smoothness.

**Fig. 3:**
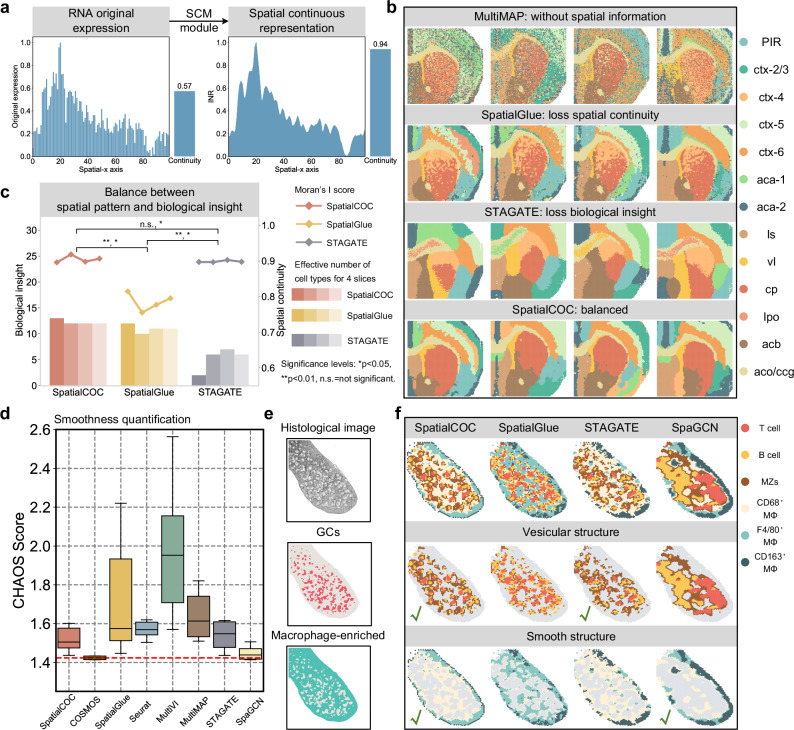
Spatial domain identifications in mouse brain and mouse spleen datasets. **a** Spatially continuous representation of RNA modality from ATAC slice in the mouse brain dataset. The average normalized expression along the x-axis was compared before and after reconstruction using the Spatial Continuous Mapping (SCM) module, with spatial continuity evaluated by Moran’s I score (*n* = 100 coordinates along the *x*-axis). **b** Spatial domain identification by different types of methods across four slices in the mouse brain dataset: MultiMAP, SpatialGlue, STAGATE, and SpatialCOC. Colors in the legend correspond to cell types identified from the ATAC slice. The full names of the abbreviations of brain regions are defined in Supplementary Note 5. **c** Quantification of spatial smoothness and biological insight. Moran’s I score (line plot) and number of identified biological structures (histogram) are shown. Statistical significance was determined using two-sided paired t-tests without adjustment for multiple comparisons. Significance level between groups describes spatial continuity first, and then biological insight. Spatial continuity: SpatialCOC vs. SpatialGlue, *P* = 0.006; SpatialCOC vs. STAGATE, *P* = 0.371; SpatialGlue vs. STAGATE, *P* = 0.003. Biological insight: SpatialCOC vs. SpatialGlue, *P* = 0.015; SpatialCOC vs. STAGATE, *P* = 0.014; SpatialGlue vs. STAGATE, *P* = 0.028 (*n* = 4 slices). **d** Evaluation of spatial smoothness of slice 1 in the mouse spleen dataset. The CHAOS score is calculated to evaluate the spatial smoothness of clustering results for different methods (*n* = 6 clusters for each method). A lower CHAOS score indicates better spatial smoothness. In the boxplot, the center line, box limits, and whiskers denote the median, upper, and lower quartiles, and 1.5× interquartile range, respectively. The red dashed line represents the best median CHAOS score of mono-omics clustering, serving as a reference baseline. **e** Histological validation of mouse spleen structures. The histological image of mouse spleen slice 1 is processed using standard image-processing techniques (filtering and binarization) to identify vesicular germinal centers (GCs) and smooth macrophage-enriched regions, with the corresponding areas highlighted. **f** Spatial domain identification in mouse spleen slice 1 by SpatialCOC, SpatialGlue, STAGATE, and SpaGCN, with a focus on how each method captures two types of region-specific continuous structures: vesicular and smooth. Source data are provided as a Source data file.

Analysis of the mouse brain dataset. We applied SpatialCOC to four slices from the mouse brain dataset generated using spatial ATAC-RNA-seq and spatial CUT&Tag-RNA-seq^2^. Moran’s I score quantifies spatial autocorrelation, capturing both spatial patterns and local smoothness. To quantitatively evaluate spatial reconstruction quality, we visualized the expression profile along the X-axis and computed the Moran’s I score based on the mean expression across all spots, yielding values of 0.57 before reconstruction and 0.94 after reconstruction (Fig. 3a). Similarly, we applied Moran’s I to evaluate the performance of different methods in spatial domain identification (detailed in Supplementary Note 4). SpatialGlue, STAGATE, and other spatial-integrating methods consistently outperformed non-spatial approaches in achieving higher spatial smoothness (Fig. 3b). However, these methods may overlook biological insights while pursuing spatial smoothness or focus on correlation in high dimensions but ignore the real structure of tissue. For instance, SpatialGlue captured abundant biological features but lacked overall smoothness (Moran’s I score = 0.785), whereas STAGATE maintained high overall smoothness at the expense of biological signal loss, with the effective number of cell types less than six on average. In contrast, SpatialCOC preserved a balance between spatial smoothness and biological feature retention, with an average Moran’s I score of 0.905, higher than other methods (Fig. 3b, c). SpatialCOC identified biologically meaningful structures and their marker genes across all mouse brain slices. A notable example is the neural signal transduction gene *Pde10a*, identified in the CP structure, which is consistent with prior research^29^. All clustering results, quantitative comparisons, and marker gene data are presented in Supplementary Figs. 9a–f and 10a–f, with a corresponding detailed analysis provided in Supplementary Note 5. The superiority of SpatialCOC is attributed to its use of spatial coordinates as prior information for omics modalities, allowing it to capture global spatial patterns without sacrificing biological signals. By contrast, methods that treat spatial and omics modalities as parallel entities and integrate them through “weighted summation” face challenges in weight optimization, especially with increasing modalities.

Analysis of the mouse spleen dataset. To evaluate SpatialCOC’s performance in regions with varying spatial smoothness, we analyzed two replicates from the mouse spleen dataset and computed CHAOS scores^30^ to quantify spatial smoothness (Lower CHAOS scores indicate better spatial smoothness). Globally, SpaGCN achieved the highest smoothness, followed by SpatialCOC and STAGATE (Fig. 3d). The histological image was processed using image filtering and binarization to highlight vesicular GCs and relatively smooth macrophage-enriched regions (Fig. 3e). The spatial domain identification of each method was compared to these histological images. While SpaGCN exhibited good smoothness in the macrophage-enriched areas, it failed to identify GC structure accurately. Conversely, STAGATE identified GC structure but struggled with smoothness in the macrophage-enriched areas. Notably, SpatialCOC achieved region-specific smoothness, accurately recognizing the vesicular GC structure while maintaining smoothness in peripheral macrophage-enriched areas (Fig. 3f). Cluster validation was performed using both marker genes and proteins. Subsequently, the identity of macrophage (MΦ) subclusters was definitively annotated based on the enriched expression of canonical protein markers, including CD68, F4/80, and CD163. The clustering results of the two mouse spleen sections and the corresponding marker analyses are shown in Supplementary Figs. 11a–d and 12a–d, respectively. While analysis of the RNA modality revealed that genes like mt-Co2 were common to both T and B cells—reflecting the heightened mitochondrial activity necessary for lymphocyte proliferation during the GC reaction—the integrated multi-modal analysis revealed a significant upregulation of CD3 in T cells and elevated expression of CD19 and IgD in B cells at the protein level, thereby corroborating the biological validity of the classified clusters^31^. Detailed multimodal biomarker analyses are provided in Supplementary Note 5. The performance highlights SpatialCOC’s adaptability in complex patterns where multiple distinct spatial patterns coexist within the same slice. Structured approaches like graph neighborhoods lack the local specificity required for such tasks. In contrast, SpatialCOC’s SCM module allows it to capture fine-grained and spatial continuous details, offering flexible adaptation to regions with heterogeneous spatial patterns and smoothness.

By systematically evaluating datasets with two distinct spatial patterns and levels of smoothness, we demonstrated the superiority of SpatialCOC’s SCM module over graph neighborhood-based structured approaches. It can effectively balance biological significance and spatial patterns on a global scale while accommodating local heterogeneity. This capability positions SpatialCOC as a flexible tool for integrating spatial information with multi-omics data.

### Achieving robust, batch-consistent trajectory inference on mouse thymus dataset

After integrating spatial information with each omics modality, we subsequently focus on integrating multiple omics modalities while ensuring seamless compatibility with downstream tasks (e.g., trajectory inference). For meaningful biological insights, it is crucial that replicate slice experiments yield consistent results, highlighting the need for robust, noise-resistant integration methods. We applied SpatialCOC to mouse thymus dataset^32^ to demonstrate its capability for robust and batch-consistent integration across modalities.

**Fig. 4:**
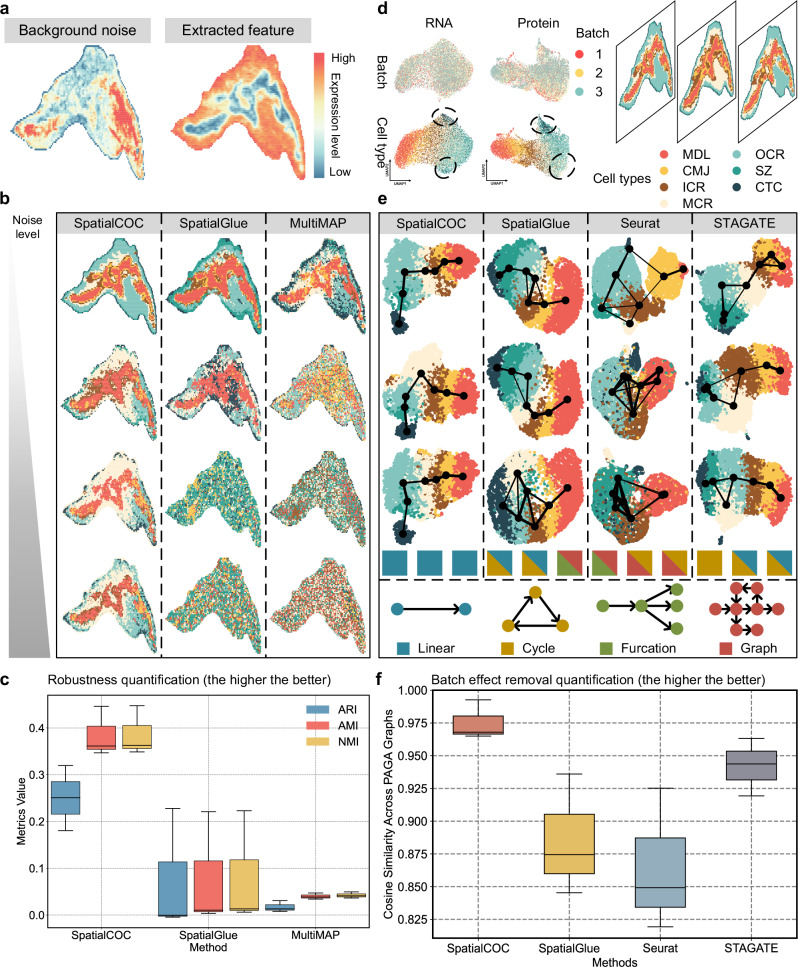
Robustness test and trajectory inference on mouse thymus replicates. **a** Comparison of “background noise” and feature extraction. Taking the RNA modality of replicate 1 from the mouse thymus dataset as an example, the spatial distributions were shown for both the “background noise” and for the first embedding extracted by the Cross-Omics Correction (COC) module. **b** Robustness test for spatial domain identification of three methods. Unsupervised clustering results for SpatialCOC, SpatialGlue, and MultiMAP are compared across increasing noise levels, displayed from top to bottom as no noise followed by three gradient-increasing noise levels. Consistent with **e**, different colors represent distinct cell types, and their correspondence can be found in (**d**). **c** Quantitative measurement of robustness using adjusted rand index (ARI). ARI values are calculated to measure the robustness of spatial domain identification under varying noise levels (*n* = 3 noise levels for each method). In the boxplot, the center line, box limits, and whiskers denote the median, upper, and lower quartiles, and 1.5× interquartile range, respectively. **d** Left: dimensionality reduction visualization of batch and cell type mixture. Uniform Manifold Approximation and Projection (UMAP) applied to three mouse thymus slices reveals discernible batch effects in both omics. Right: spatial domain identification in the three replicates of the mouse thymus dataset using SpatialCOC. **e** Trajectory inference performance. UMAP visualizations of partition-based graph abstraction (PAGA) for each of the three slices show the relationships among cell types individually. Line thickness indicates the strength of the connection among cell types. The trajectories inferred by SpatialCOC, SpatialGlue, Seurat, and STAGATE on each slice can be classified into four topological categories or their combinations: linear, cycle, furcation, and graph. **f** Quantitative measurement of batch effect removal. The cosine similarity of node-aligned PAGA graphs was calculated to compare how well each method maintained consistent spatial neighborhood structures across replicates (*n* = 3 pairwise comparisons). Boxplot representation follows the same format as in (**c**). Source data are provided as a Source data file.

Single-slice robustness analysis. We first evaluated SpatialCOC’s performance on a single slice of mouse thymus dataset. The thymus, a small gland divided into two left- and right-hand lobes connected by a fibrous isthmus, contains a central medulla and a peripheral cortex. RNA modality often exhibits “background noise” due to uneven cell density^33^, which can obscure accurate feature extraction. Using SpatialCOC, we extracted features that overcame these defects, revealing clear spatial domains corresponding to the cortex, medulla, and connective tissue (Fig. 4a). To evaluate robustness against noise which is common in spatial transcriptome data, we artificially added Gaussian noise to the original data. While methods like SpatialGlue and MultiMAP showed significant deviations in spatial domain identification as noise increased, SpatialCOC consistently identified core areas, such as the central medulla, outer cortex, and connective tissue (Fig. 4b). The ARI further confirmed SpatialCOC’s superior performance under varying noise levels, with higher ARI values compared to SpatialGlue and MultiMAP (Fig. 4c). This robustness is attributed to SpatialCOC’s “correction”-based integration, which effectively removes noise lacking inter-modal correlation. In contrast, methods relying on “weighted summation” merely reduce the impact of noisy modalities^14^, rather than removing the noise entirely.

Batch-consistent trajectory inference. Next, we extended the analysis to three parallel slices of the mouse thymus dataset serving as biological replicates. These slices exhibited noticeable batch effects in the RNA modality, and the two modalities revealed distinct biological structural relationships, which might hinder a consistent biological interpretation (Fig. 4d and Supplementary Fig. 13a–c). To evaluate the reliability and consistency of cell-type and state relationships across replicates for each method, we performed trajectory inference using the partition-based graph abstraction (PAGA) algorithm^34^ (Fig. 4d, e). SpatialCOC consistently reconstructed the thymus structure across replicates, clearly identifying the central medulla and outer cortex within each lobe, separated by connective tissue at their boundary. Marker genes and proteins are calculated to validate and annotate clusters across three replicates (Supplementary Fig. 14a–c). Trajectory inference revealed a consistent linear progression from medulla through connective tissue to cortex across all replicates, aligning with the thymus structure and matching the cell-type relationship classification proposed by Wouter Saelens^35^ (Fig. 4e). Other methods failed to maintain consistent results across the slices. For instance, SpatialGlue identified a linear relationship in slice two but a graph relationship in slice three. The consistency across batches can be quantified by calculating the cosine similarity of the node-aligned PAGA graphs generated by different methods (detailed in Supplementary Note 4). The average distance for SpatialCOC was 0.975, lower than the second-ranked STAGATE’s 0.942 (Fig. 4f). This inconsistency underscores the limitations of traditional methods in addressing batch effects. By contrast, SpatialCOC effectively removed batch effects through the mutual correction of modalities within an individual slice, enhancing downstream analysis reliability.

SpatialCOC achieves inter-modality integration through cross-correction, addressing challenges posed by experimental artifacts, such as RNA “background noise”, multimodal interference, and batch effects among replicates. Unlike methods based on “weighted summation”, which reduces the impact of noisy modalities without eliminating the noise itself^14^, SpatialCOC directly removes the underlying noise. This robust approach enhances the interpretability and reliability of downstream analyses.

## Discussion

SpatialCOC is a computational framework designed to treat spatial information as prior knowledge to learn omics-specific spatial distribution functions, uncovering nonlinear correlations among them. Through systematic evaluations across tissue types and platforms, SpatialCOC demonstrated superior performance compared to seven existing multi-omics integration and spatial mono-omics methods. It consistently achieves region-specific continuous spatial domain identifications and preserves batch-consistent trajectory inferences. These results highlight the contributions of its two core modules: (1) Spatially Continuous Reconstruction of Multi-omics Modalities through the SCM module; (2) Integration Among omics Modalities through the COC module. Future improvements of SpatialCOC will also focus on these two core modules.

The SCM module enables continuous representation of omics modalities at the shared spatial coordinates, facilitating implicit integration of spatial information. Extending the SCM module to handle non-aligned data will be a key focus for future development, which will require systematic alignment strategies. Building upon the demonstrated effectiveness across diverse omics datasets, another promising future direction involves its extension to multi-scale modality integration, encompassing histological image data, tissue microenvironment characteristics, and temporal dynamic patterns. For example, in integrating image data, the resolution disparity between images and measurement spots often results in information loss. Effective interpolation and mapping strategies are essential to align these data types. The SCM module enables precise data representation across scales, it could advance high-precision, multi-scale spatial data integration.

The COC module excels at integrating modalities with arbitrary feature dimensions, such as raw data matrices or dimensionality-reduced representations. This flexibility enables it to handle data combinations with vastly different dimensions effectively. It primarily focuses on vertical integration, requiring all modalities to correspond to measurements of the same cell. This restricts its application to horizontal integration and mosaic integration tasks across slices. Despite these limitations, the COC module has demonstrated strong potential for batch effect correction through mutual correction within single slices. A promising future direction is to extend the COC module cell embedding to feature embedding, enabling cross-horizontal and vertical integration across sections and omics data. This enhancement would allow this module to process more complex datasets, such as those spanning different tissue types and time points, thereby uncovering deeper biological insights.

Overall, SpatialCOC offers a two-stage framework for integrating spatial multi-omics data, opening new avenues for exploring relationships between spatial information and multiple omics modalities. While it currently focuses on vertical integration tasks, SpatialCOC has significant potential to be extended for horizontal and mosaic integration tasks^36^. As multi-modal, multi-dimensional, and across-scale data accumulate, SpatialCOC can unlock modalities’ potential and reveal their interaction mechanisms. There are several avenues for extending SpatialCOC. One such avenue is incorporating temporal dynamics and additional multi-omics dimensions, which will enhance its ability to model scalable spatiotemporal regulatory networks.

## Methods

### Data

#### Human lymph node (HLN)-augmented datasets

Authentic omics data from the Human Lymph Node dataset^23^ were selected for four cell types with the highest spot counts (medulla cords, medulla sinuses, cortex, and pericapsular adipose tissue). For each cell type, 300 spots were randomly sampled (1200 spots in total) and divided into three replicate groups. Four spatial distribution patterns were generated inspired by the work of Yang et al.^10^, each comprising four regions designated for specific cell types with assigned spatial coordinates: (1) quadrant pattern: a simple spatial distribution with well-separated regions; (2) stripe pattern: a moderate spatial complexity with gradual transitions between regions, forming stripe-like patterns; (3) arc pattern: a more intricate spatial pattern with gradual transitions between regions, exhibiting curved patterns; (4) layered pattern: the most complex spatial structure, characterized by concentric, progressively changing regions. To simulate technical noise, four different noise combinations were designed by adding Gaussian noise and pepper noise (“dropout” events). The four combinations were generated by applying two types of noise to modalities in all possible arrangements. Three noise levels were applied to both modalities. The coordinates generated by the arc spatial pattern are used as the spatial information across noise combinations. The details of spatial coordinates and noise generation are provided in Supplementary Note 6 and Supplementary Table 1.

#### Mouse brain dataset

We obtained the dataset for four brain tissue sections from a juvenile (P22) mouse based on ATAC–RNA-seq (co-sequencing of chromatin accessibility and gene expression) and CUT&Tag-RNA-seq (profiling histone modifications), as developed by Zhang et al.^2^. The dataset included four slices: ATAC, H3K27me3, H3K27ac, and H3K4me3, with an average of 9471 spots per sample. The ATAC sample was selected as the primary analysis instance, while the results for all samples are provided in Supplementary Figs. 9a–f and 10a–f. In the filtering step, genes with nonzero expression in at least 10 spots were retained, and spots expressing at least 200 genes were included in further analysis.

#### Mouse spleen dataset

We obtained the dataset of two mouse spleen sections (biological replicates 1 and 2) based on SPOTS (Spatial PrOtein and Transcriptome Sequencing), as developed by Ben-Chetrit et al.^3^. Each section captures both gene expression and protein abundance, with 32,285 genes and 21 proteins captured per spot. The number of spots is 2568 and 2768 spots for the two replicates, respectively. Filtering criteria included retaining genes expressed in at least 10 spots.

#### Mouse thymus dataset

We obtained the Stereo-CITE-seq dataset of three mouse thymus sections using Stereo-CITE-seq, as developed by Liao et al.^32^. Each sample contains an average of 4376 spots. Filtering criteria included retaining genes with nonzero expression in at least 10 spots, spots expressing at least 80 genes, and proteins detected in at least 50 spots.

To ensure the robust and generalization of SpatialCOC’s evaluation, we analyzed the spatial patterns and noise interference present in the benchmark datasets (Supplementary Note 7 and Supplementary Fig. 15). The datasets used for SpatialCOC’s evaluation have been summarized in Supplementary Table 2.

### Data preprocessing

We used SCANPY v1.9.5 package^37^ to preprocess all datasets, ensuring consistency across integration methods. The specific steps for each modality are as follows:

#### RNA modality

For the RNA modality across all datasets employed, we selected 3000 highly variable genes using scanpy.pp.highly_variable_genes() and performed log-normalization using scanpy.pp.log1p(). The log-normalization can stabilize the variance-mean relationship of gene expression levels, while preserving the information of low-expression genes.

#### Protein modality

For the protein modality in the HLN-augmented datasets, as well as in the mouse thymus and spleen datasets, we first filtered the samples to ensure that they were aligned with the RNA modality. Then, we applied the Centered Log Ratio (CLR) transformation to normalize protein counts. The CLR transformation is suitable for antibody-derived tag (ADT) data in the protein modality, as it can eliminate differences in cell capture efficiency while preserving the relative abundance information of protein markers.

#### Epigenome modality

For the chromatin peak accessibility data in the mouse brain dataset, we employed the Term Frequency-Inverse Document Frequency (TF-IDF) method for normalization. This method captures TF, which indicates how frequently a particular chromatin peak occurs within a single cell, and IDF, which signifies the scarcity of that peak across the entire dataset. The TF-IDF method can address the issue of sparse binary features and effectively distinguish functionally related open chromatin regions.

#### Dimensionality reduction

To ensure consistent feature representation across methods, we applied different dimensionality reduction techniques based on modality. For RNA and protein modalities, we applied principal component analysis (PCA) using scanpy.pp.pca() to project data into a lower-dimensional space. For epigenome modality, we applied Latent Semantic Indexing (LSI) to reduce the dimensionality of chromosome accessibility data. Unlike other methods that require input modalities to have identical feature dimensions, SpatialCOC can process either raw features or reduced-dimension embeddings (e.g., PCA or LSI embeddings) of arbitrary dimensions. This flexibility enables the effective integration of datasets with imbalanced feature spaces, making it robust to variations in data structure.

### SpatialCOC model structure

We introduce SpatialCOC, a novel framework designed to learn a joint multimodal representation of multi-omics data by fully leveraging both expression profiles and their shared spatial location information. Unlike conventional methods that treat spatial information as a separate modality, SpatialCOC implicitly integrates spatial information by modeling omics data as a continuous function that maps an arbitrary spatial coordinate to the corresponding spot expression, hence resulting in a spatially continuous representation of omics data. This allows SpatialCOC to effectively capture nonlinear correlations of multi-omics data within a spatial context.

SpatialCOC is a two-stage framework designed for integrating spatial multi-omics datasets with different omics modalities: The first stage involves implicit neural representations (INRs), which refer to the functions that map any spatial coordinate to the corresponding spot expression, to construct spatially continuous representations of individual omics modalities. The second focuses on correlation subspace learning that captures cross-modal relationships and facilitates mutual correction between different omics data.

For simplicity of illustrations, a typical spatial multi-omics dataset contains two omics is considered and formalized as a tuple ${{\bf{D}}}=({{\bf{X}}},{{{\bf{Z}}}}_{1},{{{\bf{Z}}}}_{2})$. The tuple consists of spatial coordinates and two multi-omics matrices, defined as follows:

- Spatial coordinates ${{\bf{X}}}\in {{{\bf{R}}}}^{n\times 2}$ define the 2D locations of spots and are shared by each omics modality, where $n$ denotes the number of spots, and ${{{\bf{X}}}}_{i}$ are the $i-{\mbox{th}}$ spot’s spatial coordinates in ${{\bf{X}}}$.
- Omics expression matrices ${{{\bf{Z}}}}_{1}\in {{{\bf{R}}}}^{n\times {m}_{1}},{{{\bf{Z}}}}_{2}\in {{{\bf{R}}}}^{n\times {m}_{2}}$ capture molecular profiles across two omics modalities, where ${m}_{1},{m}_{2}$ are the respective feature dimensions of the two omics modalities.

Neural networks are employed in both stages of our framework. To avoid confusion, we define the following notations:

- Embeddings: ${{{{\bf{e}}}}_{i1}}^{({{\boldsymbol{\cdot }}})},{{{{\bf{e}}}}_{i2}}^{({{\boldsymbol{\cdot }}})}$ denote the embeddings of the two omics in the SCM stage, while ${{{{\bf{h}}}}_{i1}}^{(\cdot )},{{{{\bf{h}}}}_{i2}}^{(\cdot )}$ represent the corresponding embeddings in the COC stage.
- Weights and Biases: $\left\{{{{\bf{W}}}}_{\cdot \cdot },{{{\bf{b}}}}_{\cdot \cdot }\right\}$ denote the trainable weight matrices and bias vectors in the SCM stage, while $\left\{{{{\bf{U}}}}_{\cdot \cdot },{{{\bf{c}}}}_{\cdot \cdot }\right\}$ denote those in the COC stage.

#### Spatial continuous mapping (SCM) module

Spatially adjacent spots within a tissue tend to exhibit similar cell types or cell states. Thus, the goal of the SCM learning stage is to learn spatially continuous representations of each omics modality, ensuring smooth transitions across spatial locations to capture such spatial local similarities. This is achieved by using INRs, a class of neural networks that maps an arbitrary spatial coordinate to the corresponding spot expression of an omics modality. Unlike traditional grid or spot-based methods limited by discrete spot resolution, the learned INRs could infer the spot expression at any given spatial coordinate in the continuous domain, thus allowing interpolation across unobserved spatial locations.

Due to the powerful representation abilities of neural networks, the INRs can provide high-fidelity modeling to capture complex and nonlinear patterns in omics data and exhibits resolution independence to overcome spatial granularity limitations in spot-based techniques. In Supplementary Note 1, we provide further information about this module, including the foundational mathematics and experimental validations of the relevant parameters.

Mathematically, we aim to learn the following function:

1 $${{{\bf{R}}}}^{2}\mapsto {{{\bf{R}}}}^{{m}_{1}},{{{\bf{R}}}}^{2}\mapsto {{{\bf{R}}}}^{{m}_{2}}$$

The mappings above represent the INRs between the two modalities for each pair of coordinates, parallel input of all the coordinates forms the final mapping function:

2 $$\begin{array}{c}{{{\bf{R}}}}^{n\times 2}\mapsto {{{\bf{R}}}}^{{n\times m}_{1}},{{{\bf{R}}}}^{n\times 2}\mapsto {{{\bf{R}}}}^{{n\times m}_{2}}\\ {{\bf{X}}}\mapsto {\Phi }_{1}\left({{\bf{X}}}\right),{{\bf{X}}}\mapsto {\Phi }_{2}\left({{\bf{X}}}\right)\end{array}$$

where:

- ${{\bf{X}}}\in {{{\bf{R}}}}^{n\times 2}$ is the shared spatial coordinates,
- ${\Phi }_{1}$ and ${\Phi }_{2}$ are INRs of two modalities, which continuously parameterize omics modal matrices ${{{\bf{Z}}}}_{1}\in {{{\bf{R}}}}^{n\times {m}_{1}},{{{\bf{Z}}}}_{2}\in {{{\bf{R}}}}^{n\times {m}_{2}}$.

The only constraint is that $\Phi$ shall output the omics modal features at the specified spot coordinates, the constraint ${C}_{1}$ and ${C}_{2}$ are as follows:

3 $$\begin{array}{c}{C}_{1}\left({{{\bf{Z}}}}_{1},{\Phi }_{1}\left({{\bf{X}}}\right)\right)={\Phi }_{1}\left({{\bf{X}}}\right)-{{{\bf{Z}}}}_{1}\\ {C}_{2}\left({{{\bf{Z}}}}_{2},{\Phi }_{2}\left({{\bf{X}}}\right)\right)={\Phi }_{2}\left({{\bf{X}}}\right)-{{{\bf{Z}}}}_{2}\end{array}$$

where:

- ${{{\bf{Z}}}}_{1}\in {{{\bf{R}}}}^{n\times {m}_{1}},{{{\bf{Z}}}}_{2}\in {{{\bf{R}}}}^{n\times {m}_{2}}$ are the two omics modal matrices,
- ${\Phi }_{1}({{\bf{X}}})$ and ${\Phi }_{2}({{\bf{X}}})$ are the functions of two modalities fitted via coordinates.

The constraint for training the INRs ${\Phi }_{1}$ and ${\Phi }_{2}$ are the misfit between the output and the omics model features.

Recent studies have demonstrated that neural networks utilizing periodic activation functions, known as Sine Representation Networks (SIRENs), excel at modeling complex real natural signals. Following this principle, SpatialCOC utilizes SIREM-based network for robust spatial encoding. Specifically, we take the spatial coordinates ${{\bf{X}}}\in {{{\bf{R}}}}^{n\times 2}$ as input through a linear transformation:

4 $$\begin{array}{c}{{{{\bf{e}}}}_{i1}}^{(1)}={{{{\bf{W}}}}_{11}{{\bf{X}}}}_{i}+{{{\bf{b}}}}_{11}\\ {{{{\bf{e}}}}_{i2}}^{(1)}={{{{\bf{W}}}}_{12}{{\bf{X}}}}_{i}+{{{\bf{b}}}}_{12}\end{array}$$

where:

- ${{{\bf{X}}}}_{i}$ are the $i-{\mbox{th}}$ spot’s spatial coordinates in ${{\bf{X}}}$,
- ${{{\bf{W}}}}_{11}$ and ${{{\bf{W}}}}_{12}$ are the trainable weight matrices,
- ${{{\bf{b}}}}_{11}$ and ${{{\bf{b}}}}_{12}$ are vectors of biases.

Then we pass it through three sine layers, where the $k-{\mbox{th}}$ ($k\in \{{\mathrm{1,2,3}}\}$) sine layer representation are as follows:

5 $$\begin{array}{c}{{{{\bf{e}}}}_{i1}}^{(k+1)}={{{\bf{W}}}}_{k1}\sin ({{{\boldsymbol{\omega }}}}_{0}\cdot ({{{{\bf{e}}}}_{i1}}^{(k)}\otimes {{{\bf{1}}}}_{3}))+{{{\bf{b}}}}_{k1}\\ {{{{\bf{e}}}}_{i2}}^{(k+1)}={{{\bf{W}}}}_{k2}\sin ({{{\boldsymbol{\omega }}}}_{0}\cdot ({{{{\bf{e}}}}_{i2}}^{(k)}\otimes {{{\bf{1}}}}_{3}))+{{{\bf{b}}}}_{k2}\end{array}$$

where:

- ${{{\boldsymbol{\omega }}}}_{0}$ is the frequency vector, and we set it to $[{\mathrm{1,2,3}}]$,
- $\otimes$ represents the out product,
- ${{{\boldsymbol{1}}}}_{3}$ donates an all-one 3D vector,
- ${{{\bf{b}}}}_{k1}$ and ${{{\bf{b}}}}_{k2}$ are vectors of biases.

The above formula can be interpreted as follows: By initializing with different ${{{\boldsymbol{\omega }}}}_{0}$ values, we construct three sub-networks for each modality to perform fitting. This enables the networks to capture signals of different frequencies, and enhances their fitting ability. Finally, through a linear layer, the spatially continuous reconstructions of the two modalities are obtained through:

6 $$\begin{array}{c}{\Phi }_{1}({{\bf{X}}})={{{\bf{W}}}}_{41}{{{{\bf{e}}}}_{i1}}^{(4)}+{{{\bf{b}}}}_{41}\\ {\Phi }_{2}({{\bf{X}}})={{{\bf{W}}}}_{42}{{{{\bf{e}}}}_{i2}}^{(4)}+{{{\bf{b}}}}_{42}\end{array}$$

where ${\Phi }_{1}({{\bf{X}}})$ and ${\Phi }_{2}({{\bf{X}}})$ are obtained by feeding the coordinates ${{\bf{X}}}$ into the INRs. The fitted functions are spatially continuous because the outputs of adjacent input coordinates tend to share similar expressions due to the smoothness of function representation. Moreover, this approach overcomes the limitations imposed by the resolution constraints of discrete spot detection. We denote the representations generated by ${\Phi }_{1}({{\bf{X}}})$ and ${\Phi }_{2}({{\bf{X}}})$ as ${\hat{{{\bf{Z}}}}}_{1}={ \{ {{\hat{{{\bf{z}}}}}_{i1}} \}}_{i=1}^{n}\in {{{\bf{R}}}}^{n\times {m}_{1}},{\hat{{{\bf{Z}}}}}_{2}={ \{ {{\hat{{{\bf{z}}}}}_{i2}} \} }_{i=1}^{n}\in {{{\bf{R}}}}^{n\times {m}_{2}}$.

#### Cross-omics correction (COC) module

The COC module is designed to learn nonlinear relationships across different omics modalities while preserving their individual structural integrity. This is crucial because multi-omics data often exhibit heterogeneous distributions, differing feature scales, and complex nonlinear correlations.

To address these challenges, the COC module consists of an encoder to capture latent representations for each modality, a decoder to reconstruct the original data from latent embeddings, and a linear canonical correlation analysis Layer to enhance inter-modality correlation by aligning their latent projects.

The encoder consists of $L$ layers ($L=3$), and we take the spatial continuous representations as input (${{{{\bf{h}}}}_{i1}}^{(0)}={\hat{{{\bf{z}}}}}_{i1},{{{{\bf{h}}}}_{i2}}^{(0)}={\hat{{{\bf{z}}}}}_{i2},i={\mathrm{1,2}},\cdots,n$). The first encoder layer representations of modalities are as follows:

7 $$\begin{array}{c}{{{{\bf{h}}}}_{i1}}^{(1)}={{\rm{\sigma }}}({{\rm{BN}}}({{{\bf{U}}}}_{11}{{{{\bf{h}}}}_{i1}}^{(0)}+{{{\bf{c}}}}_{11}))\\ {{{{\bf{h}}}}_{i2}}^{(1)}={{\rm{\sigma }}}({{\rm{BN}}}({{{\bf{U}}}}_{12}{{{{\bf{h}}}}_{i2}}^{(0)}+{{{\bf{c}}}}_{12}))\end{array}$$

where:

- ${{\rm{\sigma }}}(\cdot )$ is the nonlinear activation function,
- ${{\rm{BN}}}(\cdot )$ is the batch normalization layer at the first layer of the encoder,
- ${{{\bf{U}}}}_{11}$ and ${{{\bf{U}}}}_{12}$ are the trainable weight matrices,
- ${{{\bf{c}}}}_{11}$ and ${{{\bf{c}}}}_{12}$ are vectors of biases.

The $k-{\mbox{th}}$ ($k\in \{2,\cdots,L-1,L\}$) encoder layer representations of modalities are as follows:

8 $$\begin{array}{c}{{{{\bf{h}}}}_{i1}}^{(k)}={{\rm{\sigma }}}({{{\bf{U}}}}_{k1}{{{{\bf{h}}}}_{i1}}^{(k-1)}+{{{\bf{c}}}}_{k1})\\ {{{{\bf{h}}}}_{i2}}^{(k)}={{\rm{\sigma }}}({{{\bf{U}}}}_{k2}{{{{\bf{h}}}}_{i2}}^{(k-1)}+{{{\bf{c}}}}_{k2})\end{array}$$

The definitions of ${{\rm{\sigma }}}(\cdot )$, ${{{\bf{U}}}}_{k\cdot }$, and ${{{\bf{c}}}}_{k\cdot }$ follow the same formulation as in the first encoder layer, where the subscript $k$ indicates that these elements belong to the $k-{\mbox{th}}$ layer.

The decoder is designed to reconstruct the original data or generate new representations from the latent embeddings. Specifically, by taking the output of the encoder as input (i.e., ${{\hat{{{\bf{h}}}}}_{i1}}^{(L)}={{{{\bf{h}}}}_{i1}}^{(L)},{{\hat{{{\bf{h}}}}}_{i2}}^{(L)}={{{{\bf{h}}}}_{i2}}^{(L)}$), The $k-{\mbox{th}}$ ($k\in \{1,\cdots,L-1,L\}$) decoder layer representations of modalities are as follows:

9 $$\begin{array}{c}{{\hat{{{\bf{h}}}}}_{i1}}^{(k-1)}={{\rm{\sigma }}}({\hat{{{\bf{U}}}}}_{k1}{{\hat{{{\bf{h}}}}}_{i1}}^{(k)}+{\hat{{{\bf{c}}}}}_{k1})\\ {{\hat{{{\bf{h}}}}}_{i2}}^{\left(k-1\right)}={{\rm{\sigma }}}({\hat{{{\bf{U}}}}}_{k1}{{\hat{{{\bf{h}}}}}_{i2}}^{(k)}+{\hat{{{\bf{c}}}}}_{k2})\end{array}$$

The output of the auto-encoder is considered as the reconstructed normalized expressions or dimensionality reductions of the two modalities, represented as follows:

10 $$\begin{array}{c}{{{\bf{H}}}}_{1}={{{{\bf{h}}}}_{i1}}^{L}={{\rm{\sigma }}}({{{\bf{U}}}}_{k1}{{{{\bf{h}}}}_{i1}}^{L-1}+{{{\bf{c}}}}_{k1})\\ {{{\bf{H}}}}_{2}={{{{\bf{h}}}}_{i2}}^{L}={{\rm{\sigma }}}({{{\bf{U}}}}_{k2}{{{{\bf{h}}}}_{i2}}^{L-1}+{{{\bf{c}}}}_{k2})\end{array}$$

where:

- ${{{\bf{H}}}}_{1}={ \{ {{\hat{{{\bf{h}}}}}_{i1}} \}}_{i=1}^{n}\in {{{\bf{R}}}}^{n\times {m}_{1}}$ and ${{{\bf{H}}}}_{2}={ \{ {{\hat{{{\bf{h}}}}}_{i2}} \}}_{i=1}^{n}\in {{{\bf{R}}}}^{n\times {m}_{2}}$ are the output of the auto-encoder,
- The definitions of ${{\rm{\sigma }}}(\cdot )$, ${{{\bf{U}}}}_{k\cdot }$, and ${{{\bf{c}}}}_{k\cdot }$ are the same as those in the encoder layers.

Since the autoencoder can take either raw features or reduced-dimension embeddings (e.g., PCA or LSI embeddings) of arbitrary dimensions as inputs, the dimensionalities of the outputs are also flexible.

To further enhance the extraction of features exhibiting high correlation among modalities, we incorporate a linear canonical correlation analysis layer. It strengthens the inter-modality correlation while preserving the orthogonality among features within each modality. Specifically, for the outputs produced by the autoencoder, this layer finds pairs of linear projections of the two modalities that maximize their correlation:

11 $$\begin{array}{c}({w}_{1}^{*},{w}_{2}^{*})={{{\rm{argmax}}}}_{{w}_{1},{w}_{2}}{{\rm{corr}}}({w}_{1}^{{\prime} }{{{\bf{H}}}}_{1},{w}_{2}^{{\prime} }{{{\bf{H}}}}_{2})={{{\rm{argmax}}}}_{{w}_{1},{w}_{2}}\frac{{w}_{1}^{{\prime} }{\Sigma }_{12}{w}_{2}}{\sqrt{{w}_{1}^{{\prime} }{\Sigma }_{11}{w}_{1}{w}_{2}^{{\prime} }{\Sigma }_{22}{w}_{2}}}\\ s.t.{w}_{1}^{{\prime} }{\Sigma }_{11}{w}_{1}={w}_{2}^{{\prime} }{\Sigma }_{22}{w}_{2}=1\end{array}$$

where:

- $({{{\bf{H}}}}_{1},{{{\bf{H}}}}_{2})$ are the outputs produced by the autoencoder,
- $({w}_{1}^{{\prime} }{{{\bf{H}}}}_{1},{w}_{2}^{{\prime} }{{{\bf{H}}}}_{2})$ are the pairs of linear projections of $({{{\bf{H}}}}_{1},{{{\bf{H}}}}_{2})$,
- $({w}_{1}^{*},{w}_{2}^{*})$ are the optimal transformation vectors that maximize the correlation between modalities.

We constrain that the variances of the projections be normalized to ensure that the canonical variates are scaled appropriately, facilitating the identification of the most informative linear combinations within each modality.

To ensure that each newly identified pair of projection vectors captures information that is distinct and not previously contained, we constrain orthogonality of $({w}_{1}^{i},{w}_{2}^{j})$, that is ${w}_{1}^{i}{\Sigma }_{11}{w}_{1}^{j}={w}_{2}^{i}{\Sigma }_{22}{w}_{2}^{j}=0$ for $i < j$. We denote the top $k$ identified vectors as $\left({{{\bf{W}}}}_{1}^{{top}},\,{{{\bf{W}}}}_{2}^{{top}}\right)$, where ${{{\bf{W}}}}_{1}^{{top}}\in {{{\bf{R}}}}^{{n}_{1}\times k},{{{\bf{W}}}}_{2}^{{top}}\in {{{\bf{R}}}}^{{n}_{2}\times k}$. We identify the top $k$ projections as follows:

12 $$\begin{array}{c}{\mbox{maximize}}:\,{\mbox{tr}}({{{\bf{W}}}}_{1}^{{top}{\prime} }{{\Sigma }_{12}{{\bf{W}}}}_{2}^{{top}})\\ {subject}\,{to}:{{{\bf{W}}}}_{1}^{{top}{\prime} }{\Sigma }_{11}{{{\bf{W}}}}_{1}^{{top}}={{{\bf{W}}}}_{2}^{{top}{\prime} }{\Sigma }_{22}{{{\bf{W}}}}_{2}^{{top}}={{\rm{I}}}\end{array}$$

To solve the above optimization problem, we first obtain the decentralization matrix for the outputs of the two modalities: $({\bar{{{\bf{H}}}}}_{1},{\bar{{{\bf{H}}}}}_{2})$, and then calculate covariance matrices as follows^38^:

13 $$\begin{array}{c}{\Sigma }_{12}=\frac{1}{m-1}{\bar{{{\bf{H}}}}}_{1}^{{{\rm{T}}}}{\bar{{{\bf{H}}}}}_{2}\\ {\Sigma }_{11}=\frac{1}{m-1}{\bar{{{\bf{H}}}}}_{1}^{{{\rm{T}}}}{\bar{{{\bf{H}}}}}_{1}+{r}_{1}{{{\bf{I}}}}_{{o}_{1}}\\ {\Sigma }_{22}=\frac{1}{m-1}{\bar{{{\bf{H}}}}}_{2}^{{{\rm{T}}}}{\bar{{{\bf{H}}}}}_{2}+{r}_{2}{{{\bf{I}}}}_{{o}_{2}}\end{array}$$

where:

- ${\Sigma }_{11}$ and ${\Sigma }_{22}$ are covariances of ${\bar{{{\bf{H}}}}}_{1}$ and ${\bar{{{\bf{H}}}}}_{2}$,
- ${\Sigma }_{12}$ is the cross-covariance of ${\bar{{{\bf{H}}}}}_{1}$ and ${\bar{{{\bf{H}}}}}_{2}$,
- ${r}_{1},{r}_{2} > 0$ are regularization parameters, which makes the covariance matrix nonsingular and prevents overfitting,
- ${{{\bf{I}}}}_{{o}_{1}}$ and ${{{\bf{I}}}}_{{o}_{2}}$ are identity matrices of size ${o}_{1}$ and ${o}_{2}$.

We define $T$ as ${\Sigma }_{11}^{-1/2}{{\Sigma }_{12}\Sigma }_{22}^{-1/2}$ and let ${U}_{k}$ and ${V}_{k}$ be the matrices of the first $k$ left- and right-singular vectors of $T$. Then the optimal objective value is the sum of the top *k* singular values of $T$ and the optimum is as follows:

14 $$({{{\bf{W}}}}_{1}^{{top}*},{{{\bf{W}}}}_{2}^{{top}*})=({\Sigma }_{11}^{-1/2}{U}_{k},{\Sigma }_{22}^{-1/2}{V}_{k})$$

Then the final spot embeddings are obtained by:

15 $$\begin{array}{c}{{\boldsymbol{embeddin}}}{{{\boldsymbol{g}}}}_{1}={\bar{{{\bf{H}}}}}_{1}{w}_{1},{w}_{1}\in {{{\bf{W}}}}_{1}^{{top}*}\\ {{\boldsymbol{embeddin}}}{{{\boldsymbol{g}}}}_{2}={\bar{{{\bf{H}}}}}_{2}{w}_{2},{w}_{2}\in {{{\bf{W}}}}_{2}^{{top}*}\end{array}$$

In the above process, we obtained the final multimodal integrated representation $({{\boldsymbol{embeddin}}}{{{\boldsymbol{g}}}}_{1},{{\boldsymbol{embeddin}}}{{{\boldsymbol{g}}}}_{2})$, which strengthens the inter-modality correlation while preserving the orthogonality. This enables us to better address downstream analysis tasks, such as spatial domain identifications, and trajectory references.

### Model training of SpatialCOC

SpatialCOC employs an unsupervised modular learning paradigm. After preprocessing unlabeled data, we construct a deep learning integrative framework, define optimization objectives, and obtain integrated multi-modal representations, which are then applied to downstream analytical tasks for performance evaluation (Supplementary Fig. 16). Modular training enables the two modules to focus on their respective optimization goals without interference while allowing direct observation of each module’s outputs, ensuring effective and verifiable feature learning. This design also supports functional coordination: the spatially continuous representations learned by the SCM module serve as input to the COC module, enabling the corrections performed by the COC module to be based on both “omics expression” and “spatial information” dimensions. The detailed procedure is as follows:

#### Mono-modality representation

We introduce an optional spatial smoothness regularization, which aims to enhance local spatial smoothness by explicitly modeling the spatial correlation structure across tissue locations with a Gaussian kernel matrix, thereby propagating information between adjacent spots. The kernel function ${{\bf{K}}}$ is defined as:

16 $${{\bf{K}}}({{{\bf{X}}}}_{i},{{{\bf{X}}}}_{j})=\exp \left(-\frac{{{\Vert }}{{{\bf{X}}}}_{i}-{{{\bf{X}}}}_{j}{{{\Vert }}}_{2}}{2{\sigma }^{2}}\right)$$

where:

- ${{{\bf{X}}}}_{i},{{{\bf{X}}}}_{j}$ are the spatial coordinates of $i-{\mbox{th}}$ and $j-{\mbox{th}}$ spot,
- ${{\rm{\Vert }}}{{{\bf{X}}}}_{i}-{{{\bf{X}}}}_{j}{\Vert }_{2}$ represents the Euclidean distance between the two spots,
- $\sigma$ regulates the sensitivity to spatial distance and is consistently set to 0.002 across the four datasets utilized.

This consistent setting of $\sigma$ suggests that the chosen value aims to provide a balanced sensitivity to spatial distance, which is deemed appropriate for the characteristics of these datasets. As an optional parameter, we have discussed in detail its effect and selection in Supplementary Note 1.

According to the constraint defined by Eq. (3), we define the loss function for mono-modality learning as follows:

17 $$\begin{array}{c}{L}_{{\mbox{IN}}{{\mbox{R}}}_{1}}={{||}{\hat{{{\bf{Z}}}}}_{1}-{{\bf{K}}}{{{\bf{Z}}}}_{1}{||}}_{2}\\ {L}_{{\mbox{IN}}{{\mbox{R}}}_{2}}=\Vert {\hat{{{\bf{Z}}}}}_{2}-{{\bf{K}}}{{{\bf{Z}}}}_{2}{\Vert }_{2}\end{array}$$

where:

- ${{\bf{K}}}$ is the kernel function calculated by spatial coordinates,
- ${{{\bf{Z}}}}_{1}$ and ${{{\bf{Z}}}}_{2}$ are the inputs of the SCM module,
- ${\hat{{{\bf{Z}}}}}_{1}$ and ${\hat{{{\bf{Z}}}}}_{2}$ are the reconstructions of ${{{\bf{Z}}}}_{1}$ and ${{{\bf{Z}}}}_{2}$, respectively.

These loss functions are joint matrix formulations of n-independent fidelity losses, where each fidelity loss is the misfit between the output and the corresponding spot expression by giving an input spot coordinate.

#### Multimodal integration

In the second state, SpatialCOC integrates multiple omics modalities into a shared latent space while ensuring the preservation of individual modality structures and alignment of different modalities to enhance biological insights.

To achieve this, we jointly optimize two complementary objects: Reconstruction Loss to ensure that each modality retains key biological information and correction loss to encourage alignment between the latent spaces of different modalities.

The Reconstruction Loss ensures that the learned latent representation retain critical information from their respective omics modalities:

18 $${L}_{{recon}}=\frac{1}{2}{\sum }_{i=1}^{n}{{||}{\hat{{{\bf{z}}}}}_{i1}-{\hat{{{\bf{h}}}}}_{i1}{||}}_{2}+\frac{1}{2}{\sum }_{i=1}^{n}{{||}{\hat{{{\bf{z}}}}}_{i2}-{\hat{{{\bf{h}}}}}_{i2}{||}}_{2}$$

where:

- ${\hat{{{\bf{z}}}}}_{i1}$ and ${\hat{{{\bf{z}}}}}_{i2}$ are the spatial continuous reconstructions of two omics modalities employed by the SCM module,
- ${\hat{{{\bf{h}}}}}_{i1}$ and ${\hat{{{\bf{h}}}}}_{i2}$ are the features extracted by the COC module.

By minimizing this loss, SpatialCOC ensures that modality-specific biological signals are preserved while facilitating cross-modal integration.

Correction loss is incorporated to align the latent spaces of different modalities, maximizing the correlation between transformed embeddings:

19 $${L}_{{CCA}}=-\sqrt{{\mbox{tr}}({T}^{{{\rm{T}}}}T)}$$

where:

- $T$ is defined as ${\Sigma }_{11}^{-1/2}{{\Sigma }_{12}\Sigma }_{22}^{-1/2}$, ${\Sigma }_{11}$, ${\Sigma }_{12}$, and ${\Sigma }_{22}$ are the nonsingular covariance matrices, ${{\rm{tr}}}(\cdot )$ denotes the trace operation, summing up the highest correlations.

By maximizing cross-modal correlation, this loss function ensures that shared biological signals across different modalities are effectively captured and aligned.

#### Final training objective

The overall training objective for SpatialCOC combines both stages into a unified loss function:

20 $${L}_{{integrate}}={L}_{{recon}}+{L}_{{CCA}}$$

where modality-specific preservation and cross-modal alignment are balanced and this joint optimization ensures that the model effectively learns both spatially smooth mono-modality representations and well-aligned multimodal embeddings.

After processing spatial multi-omics data using the SpatialCOC framework, the spatially smoothed, noise-reduced, and orthogonal omics features are obtained. These processed features can be directly utilized for various downstream analyses, including but not limited to spatial domain identification and trajectory inference.

### Benchmarking and downstream analysis

#### Benchmarking

We compared SpatialCOC with seven state-of-the-art integrative methods, each benchmarking method was applied according to its official vignette (see Supplementary Note 3 for details).

SpatialGlue v1.5.5 (https://github.com/JinmiaoChenLab/SpatialGlue),

COSMOS v1.0.0 (https://github.com/Lin-Xu-lab/COSMOS),

Seurat WNN v4.0.0 (https://github.com/satijalab/seurat),

MultiVI v1.3.1 (https://github.com/scverse/scvi-tools),

MultiMAP v0.0.1 (https://github.com/Teichlab/MultiMAP),

SpaGCN v1.2.7 (https://github.com/jianhuupenn/SpaGCN),

STAGATE v1.0.1 (https://github.com/QIFEIDKN/STAGATE).

#### Spatial clustering

We applied our proposed framework to integrate latent representations from multimodal data and performed spatial clustering analysis. After evaluating clustering performance, we chose the “leiden” algorithm^39^ for the mouse brain dataset and the “mclust” algorithm^40^ for all other datasets. We conducted extensive experiments with different cluster numbers, comparing them against known biological structures or cell types to determine the most appropriate clustering parameters.

#### Multimodal biomarker analysis

Using SCANPY package^37^, we performed differential expression analysis based on the spatial domain identification results. Specifically, we first calculated a hierarchical cluster tree using the sc.tl.dendrogram() function, and then identified differentially expressed genes in each cluster using the sc.tl.rank_genes_groups() function. The same analytical pipeline was applied to analyze marker proteins. Detailed multimodal biomarker analyses for the three datasets are provided in Supplementary Note 5.

#### Trajectory inference using Partition-based Graph Abstraction^34^ (PAGA)

After obtaining the integrated multimodal embeddings, we performed the trajectory inference task using PAGA method. We first constructed the nearest neighbor graph using the sc.pp.neighbors() with n_neighbors=50 (a parameter chosen for this dataset to ensure a fair and consistent comparison across methods). Subsequently, we performed PAGA analysis using the sc.tl.paga() with default parameters.

#### Uniform manifold approximation and projection (UMAP) dimensionality reduction visualization

The cluster relationships of the mouse thymus dataset are visualized on the UMAP graph using the sc.tl.umap() with min_dist = 0.5, spread = 0.5. The centroids of each cluster were then calculated. PAGA results were overlaid on the UMAP plot. To ensure continuity and clarity in trajectory comparisons across different methods, we applied method-specific thresholds: SpatialCOC (0.45), SpatialGlue (0.2), Seurat WNN (0.1), and STAGATE (0.2).

### Reporting summary

Further information on research design is available in the Nature Portfolio Reporting Summary linked to this article.

## Supplementary information

Supplementary Information

Reporting Summary

Transparent Peer Review file

## Source data

Source data

## Data Availability

Raw files and the count matrix of data employed in this paper are available in raw form from their original authors. Specifically, the Mouse Brain Dataset is deposited in the Gene Expression Omnibus (GEO) with accession code GSE205055. The Mouse Spleen Dataset is available from the GEO repository (accession no. GSE198353). The Mouse Thymus Dataset can be accessed via the Zenodo repository (10.5281/zenodo.7879713). The Human Lymph Node Dataset is accessible from the GEO (accession no. GSE263617). All datasets used in this paper have been uploaded to Zenodo and are freely available at 10.5281/zenodo.17655345 (ref. ^41^). Source data are provided with this paper.

## Code availability

The code used to develop the model, perform the analyses and generate results in this study is publicly available and has been deposited in Github at https://github.com/xjtu-omics/SpatialCOC, under GPL-3.0 license. The specific version of the code associated with this publication is archived in Zenodo and is accessible via 10.5281/zenodo.18595579 (ref. ^42^).
