# Supplementary material for: SpatialCOC: an integrative framework for spatial continuous mapping and cross-omics correction in spatial multi-omics data
Source: Nat Commun. 2026 Apr 16;17:5268. doi: 10.1038/s41467-026-71882-2 (PMC13265925; doi:10.1038/s41467-026-71882-2)
Supplement: Supplementary file 1 — Supplementary Information [file 41467_2026_71882_MOESM1_ESM.pdf]

Supplementary Information for

SpatialCOC: an integrative framework for capturing spatial patterns and cross-omics correction in spatial multi-omics data

Mingxuan Li†, Peisen Sun†, Yisi Luo, Guancheng Zhou,

Xiaofei Yang, Deyu Meng, Kai Ye*

† These authors contributed equally to this work.

* Correspondence to Kai Ye (kaiye@xjtu.edu.cn).

This PDF file includes:

- Supplementary Notes 1-7
- Supplementary Figures 1-16
- Supplementary Tables 1-2
- Supplementary References 1-19

Contents

Supplementary Notes .... **- 3 -**

1. Supplementary details of the SCM module... - 3 -

2. Ablation studies .... - 5 -

3. Benchmarking methods .... - 6 -

4. Evaluation metrics .... - 7 -

5. Supplementary analyses of multimodal biomarkers .... - 11 -

6. Simulations .... - 16 -

7. The datasets used for evaluation.. - 18 -

Supplementary Figures .... **- 21 -**

Supplementary Tables.. **- 51 -**

Supplementary References... **- 53 -**

Supplementary Notes

1. Supplementary details of the SCM module

We are the pioneers in applying Implicit Neural Representations (INR) to the integration of spatial multi-omics. Here, we provide additional details about this module, including the foundational mathematics and experimental validations:

(1) Foundational mathematical principles

- The Universal Approximation Theorem[1] ensures that an Implicit Neural Representation (INR) equipped with non-polynomial activation functions—such as the sinusoidal units used in Module 1—can serve as a universal function approximator. Consequently, the network can represent arbitrarily complex distributions whose gradients exhibit sharp discontinuities precisely at domain boundaries.
- Complementing this guarantee, a Fourier-theoretic view[2] reveals that the deep composition of sinusoidal layers generates a richly parameterized spectrum. This spectrum can be tuned to match the high-frequency components present in raw multi-omics measurements, enabling the model to capture abrupt expression changes without imposing a priori smoothing.

We introduced two parameters in the SCM Module:

- ω_0: used in Equation (5) of the main text, it controls the frequency of the sinusoidal activation function and determines the ability to capture global spatial information and keeping continuity;
- σ: used in Equation (16) of the main text, it is the bandwidth parameter of the Gaussian kernel, which enhances local continuity.

(2) Experimental validations of ω_0

From the perspective of Fourier analysis, a multi-layer composite structure with sine activation functions of appropriate frequencies can effectively approximate

signals containing different frequency components (low frequencies: smooth regions; high frequencies: boundaries). Therefore, as long as ω_0 remains within a certain range, this structure can simultaneously capture both smooth regions and boundary features in the signal.

Taking the mouse brain ATAC-seq dataset as an example, we conducted a comparative analysis to evaluate the reconstruction performance of sinusoidal activation functions across different frequency settings. The linear ReLU activation function served as the benchmark in this assessment.

As shown in the Supplementary Fig. 1a, b:

- The sinusoidal activation function effectively captures both smooth regions and boundary information, outperforming the linear ReLU activation.
- An excessively low value of ω_0 leads to failure in capturing edge signals, while an overly high value causes overfitting to boundary details.
- The performance remains highly stable within the range of [0.1, 10], making it straightforward to select an appropriate parameter.

(3) Experimental validations of σ

This parameter serves as a smoothness regularizer in our model. A larger value enhances the spatial smoothing effect, while a smaller value reduces it. Accordingly, we have set its default to a relatively small value of 0.002, which provides only limited local smoothing. We have tested this setting across all datasets and confirmed its consistently strong performance.

We supplemented the comparative experiments (removing Kernel and adjusting ω_0). After removing the kernel, the overall results remain stable, with only a reduction in local smoothness (Supplementary Fig. 2a, b). Based on your comment, we have made it an optional parameter. Since there is an inherent

trade-off between smoothness and boundary definition that cannot be perfectly balanced, users can now choose whether to use it based on their specific needs.

(4) The logical coherence between modules

By capturing the globally continuous spatial distribution of omics data, the SCM module enables the COC module to better model nonlinear correlation across both expression and spatial contexts.

We conducted the following comparative experiment on the mouse brain dataset: changing the order of the two modules and comparing the resulting spatial domain assignments (Supplementary Fig. 4a, b). With normal order (SCM followed by COC): Smooth and biologically meaningful results were obtained. In contrast, with the reverse order (COC followed by SCM), similar spatial smoothness was achieved, but a loss of biological significance was observed. These experimental results directly demonstrate the close logical relationship and interdependence between modules. The SCM module facilitates the COC module in uncovering non-linear associations within both expression and spatial contexts.

2. Ablation studies

To evaluate the contribution of different components in SpatialCOC, we performed four ablation experiments, with each variant tested across four simulated spatial patterns and its spatial domain identification accuracy compared against the full model. The results indicate that removing the SCM module significantly reduces spatial coherence and smoothness, while excluding the COC module completely diminishes cross-modal integration. Eliminating correction loss weakens modality correlation, and omitting reconstruction loss disrupts the preservation of modality-specific features (Supplementary Fig. 3b).

3. Benchmarking methods

To evaluate the performance of SpatialCOC, we compare it against seven state-of-the-art methods, including two spatial multi-omics data integration methods (SpatialGlue[3] and COSMOS[4]), three single-cell multi-omics data integration methods (Seurat WNN[5], MultiVI[6], and MultiMAP[7]), and two spatial transcriptome methods (STAGATE[8] and SpaGCN[9]).

After completing the unified preprocessing steps, we proceeded to evaluate each benchmarking method following the instructions provided in their respective vignettes. Below, we describe the details of each method.

SpatialGlue. Feature graphs and spatial graphs are constructed using the construct_neighbor_graph() function based on the preprocessed feature and spatial information. Subsequently, the model is trained on the neighborhood graph with default parameters to obtain an integrated latent representation.

COSMOS. An integration object is first created using the cosmos.Cosmos() function. Then, the kneighbors_graph() function from sklearn.neighbors package is used to construct the spatial graph based on spatial coordinates. Finally, the cosmos_comb.train() method is executed to obtain the integrated latent representation, cosmos_comb.embedding.

Seurat WNN. The FindMultiModalNeighbors() function is used to identify the nearest neighbors of each cell based on a weighted combination of the two modalities. The method then constructs a shared nearest neighbor (SNN) graph and performs clustering, incorporating the computed modality weights for each spot.

MultiVI. We first align and order the multi-omics data using organize_multiome_anndatas() function, consolidating different modalities into a single AnnData object. We then set batch_key="modality" when initializing the dataset with scvi.model.MULTIVI.setup_anndata() function. The model is trained using default parameters, and the integrated latent space

representation is obtained.

MultiMAP. The integration is performed using MultiMAP.Integration() function, which generates the integrated latent representation under the default parameter settings.

SpaGCN. We construct an adjacency matrix from spatial coordinates using the SpaGCN.calculate_adj_matrix() function to calculate. The number of clusters is specified, and the optimal resolution is determined using the spg.search_res() function. Finally, the model is built and spatial clustering is performed.

STAGATE. We first merge the data from the two omics modalities. Then a spatial graph is computed using STAGATE.Cal_Spatial_Net() function with rad_cutoff=10. The model is trained using STAGATE.train_STAGATE() with alpha=0, generating integrated feature representations.

4. Evaluation metrics

Noise level quantification. To quantify the impact of added noise, we calculate the signal-to-noise Ratio (SNR):

$$\mathrm{SNR}(\mathrm{dB}) = 10\log_{10}\left(\frac{P_{expression}}{P_{\mathrm{noise}}}\right) = 10\log_{10}\left(\frac{\frac{1}{n}\sum_{i,j}^{n}(x_{ij})^2}{\frac{1}{n}\sum_{i,j}^{n}(x_{ij}^{noised} - x_{ij})^2}\right) \quad (1)$$

where:

- $P_{expression}$ represents the power of the original expression data,
- P_{noise} represents the power of the data added noise,
- x_{ij} represents the original expression data of the location (i, j),
- x_{ij}^{noised} represents the noisy data of the location (i, j).

The SNR measures the relative strength of the signal compared to noise and we use it to quantify the amount of noise added.

Adjusted rand index (ARI). ARI evaluates clustering performance by quantifying the similarity between predicted and true labels, adjusting for randomness:

$$ARI = \frac{RI - E(RI)}{max(RI) - E(RI)}$$
$$RI = \frac{a+b}{n(n-1)/2} \tag{2}$$

where:

- n denotes the total number of samples,
- a is the number of clusters that belong to the same cluster as the clustering result,
- b is the number of different categories clustered as different clusters.

The ARI ranges from -1 to 1, where 1 indicates perfect clustering, 0 suggests random clustering, and -1 represents the worst case.

Adjusted mutual information (AMI). AMI measures the amount of shared information between clustering results and true labels, adjusting for randomness:

$$\mathrm{AMI} = \frac{I(X;Y) - E[I(X;Y)]}{max(H(X),H(Y)) - E[I(X;Y)]}$$
$$H(X) = -\sum_i p(x_i)logp(x_i), H(Y) = -\sum_i p(y_i)logp(y_i) \tag{3}$$

where:

- $I(X;Y)$ denotes the mutual information of the clustering results and the true labels,
- $\mathrm{H}(X), H(Y)$ are the entropy values for predicted and true labels.

The AMI ranges from -1 to 1, where 1 indicates perfect agreement, 0 suggests random, and -1 represents the worst case.

Normalized mutual information (NMI). Similar to AMI but simpler, NMI for easy comparison across clustering methods. The NMI is described as follows:

$$NMI(X;Y) = 2\frac{I(X;Y)}{H(X)+H(Y)} \tag{4}$$

the definitions of $I(X;Y), \mathrm{H}(X)$ and $H(Y)$ are the same as those defined in equation (7).

Moran's I score. It measures spatial autocorrelation, assessing the degree to which a given label or feature is significantly associated with the clustering of its spatial distribution[10]. Specifically, Moran's I score is described as follows:

$$Moran's\ I\ score = \frac{N}{W}\frac{\sum_{i=1}^{N}\sum_{j=1}^{N} w_{ij}(x_i-\overline{x})(x_j-\overline{x})}{\sum_{i=1}^{N}(x_i-\overline{x})^2} \tag{5}$$

where:

- N is the total number of spatial coordinates,
- w_{ij} are the elements of a matrix of spatial weights and $W=\sum_{i=1}^{N}\sum_{j=1}^{N} w_{ij}$,
- x are the given labels or features.

We calculated the Moran's I score using squidpy package[10] with the function squidpy.gr.spatial_autocorr().

CHAOS score. It measures the spatial continuity of given labels[11]. Specifically, for each categorical label encompassing a dataset of more than two samples, we initially calculated the Euclidean distances among all intra-class spots, then constructed a one-nearest neighbor (1NN) graph and its corresponding adjacency matrix *A*. We denote a_{ij} is element of *A*, which is calculated as:

$$a_{ij} = \begin{cases} dist(i,j) & if\ spot\ i\ is\ spot\ j's\ nearest\ neighbor \\ 0 & otherwise \end{cases} \tag{6}$$

where $dist(i,j)$ is the Euclidean distance between spot *i* and spot *j*.

Finally, for each classification category, the mean of all non-zero entries within the corresponding adjacency matrix is taken as the CHAOS score for that

particular category. For labels that consist of a solitary sample, the CHAOS score is systematically set to zero:

$$CHAOS\ score_k = \begin{cases} \dfrac{\sum_{ij}^{n_k} a_{ij}}{n_k} & if\ n_k > 1 \\ 0 & otherwise \end{cases} \tag{7}$$

where n_k is the spots total number of cluster *k*. A smaller $CHAOS\ score_k$ indicates better spatial continuity of cluster *k*.

Batch effect quantification. To quantify batch effects, we computed the cosine similarity between node-aligned PAGA graphs from different biological replicates. For two graphs represented by adjacency matrices $\boldsymbol{A}$ and $\boldsymbol{B}$ of size $n \times n$, the graph distance under a permutation π is defined as:

$$D_\pi(\boldsymbol{A}, \boldsymbol{B}) = \| \boldsymbol{A} - \boldsymbol{P}_\pi^T \boldsymbol{B} \boldsymbol{P}_\pi \|_F \tag{8}$$

where:

- $\boldsymbol{P}_\pi$ is the permutation matrix corresponding to permutation π;
- $\| \cdot \|_F$ denotes the Frobenius norm.

Then we searched all possible node permutations to find the best alignment between graphs from different replicates:

$$D_{min}(\boldsymbol{A}, \boldsymbol{B}) = \min_{\pi \in S_n} D_\pi(\boldsymbol{A}, \boldsymbol{B}) \tag{9}$$

where S_n is the symmetric group of all permutations on n elements. This optimal matching accounts for potential label switching between replicates.

Unfold the upper triangular parts of the aligned matrices $\boldsymbol{A}$ and $\boldsymbol{B}$ into two one-dimensional vectors $\boldsymbol{a}$ and $\boldsymbol{b}$, and then calculate their cosine similarity: $similarity = \dfrac{\boldsymbol{a} \cdot \boldsymbol{b}}{\| \boldsymbol{a} \| \cdot \| \boldsymbol{b} \|}$ to quantify the batch effects. This approach enables direct comparison of how well each method maintains consistent spatial neighborhood structures across biological replicates, with lower

distances indicating better reproducibility and reduced batch effects.

5. Supplementary analyses of multimodal biomarkers

To validate the spatial domains identified in the three real-world datasets, we characterized the multimodal biomarkers for each domain. These supplementary analyses provide strong molecular evidence for our conclusions.

(1) Mouse brain dataset

Based on the anatomical classification provided by the Allen Mouse Brain Atlas[12], the mouse brain can be divided into three main regions: grey (basic cell groups and regions), fiber tracts, VS (ventricular systems):

- Grey: The annotations primarily focus on the cerebrum and brain stem. The cerebrum consists of the cerebral cortex (CTX) and cerebral nuclei (CNU), while the brain stem includes the Lateral preoptic area (LPO). The CTX includes structures such as the isocortex (layers L1–L6), piriform area (PIR), and anterior cingulate area (ACA), among others. The CNU comprises the caudoputamen (CP), nucleus accumbens (ACB), and lateral septal nucleus (LS), along with other nuclei.
- Fiber tracts: Key structures include the anterior commissure (olfactory limb, aco), genu of the corpus callosum (ccg), among others.
- VS: This primarily includes components related to the ventricular systems: lateral ventricle (VL), along with other ventricles.

We annotated the aforementioned structures on the anatomical map and labeled the spatial domain segmentation results from SpatialCOC by comparing their shapes, positions, and sizes. The color annotation abbreviations for the reference atlas and clustering results are consistent with the structure abbreviations mentioned above (Supplementary Fig. 3; 4; 9a, d; 10a, d). Furthermore, we conducted an analysis of its multimodal biomarkers

(using marker genes from four slices as illustrative examples).

- Maker gene for Grey region: We identified *Pde10a* and *Mef2c* as marker genes of gray matter regions. *Pde10a* is highly enriched in the striatum—including the caudoputamen (CP) and nucleus accumbens (ACB)—where it regulates dopamine and cAMP-mediated signaling, neuronal excitability, and motor control, thereby defining the core striatal circuitry. In contrast, *Mef2c* is predominantly expressed across all layers (L1–L6) of the isocortex, where it plays a critical role in cortical neuron differentiation, synaptic development, and activity-dependent gene transcription, reflecting the layered organization of the cerebral cortex. The identification of these two genes highlights two principal components of gray matter: the striatal circuitry (*Pde10a*) and the layered architecture of the cortex (*Mef2c*).
- Maker gene for Fiber tracts region: We identified *Mobp* and *Plp1* as marker genes of the major fiber tracts. *Mobp* is a key structural component of the myelin sheath, enriched in mature oligodendrocytes and crucial for the long-term stability and compaction of myelin in the central nervous system, while *Plp1* is the most abundant protein in CNS myelin, essential for forming the functional multi-lamellar myelin structure that insulates axons and facilitates rapid saltatory conduction. The identification of these two genes defines the core molecular architecture of myelin (*Plp1*) and its mature, stable maintenance system (*Mobp*), which together ensure the structural integrity and high-fidelity signal transmission throughout the white matter tracts, such as the anterior commissure (olfactory limb, aco) and the genu of the corpus callosum (ccg).
- Maker gene for VS region: We identified *Sox4* and *Myh11* as marker genes of the lateral ventricle (VL). Sox4 drives neural progenitor

proliferation, fate specification and survival, while *Myh11* selectively labels the contractile apparatus of vascular smooth-muscle cells and pericytes that line the ventricular walls and choroid plexus. The identification of these two genes defines the core neurogenic niche (*Sox4*) and its essential vascular support system (*Myh11*) within the ventricular system.

(2) Mouse spleen dataset

As previously described (Nir Ben-Chetrit, 2023)[13], the mouse spleen can be functionally divided into the following major regions:

- Germinal Centers (GCs): Primarily composed of T cells and specific B cells.
- Marginal Zones (MZs): Located at the periphery of the germinal centers, containing another class of B cells and specialized macrophages.
- Red Pulp / Cortex: Contains fibroblasts, red pulp macrophages (scavenging macrophages), endothelial cells, and other myeloid cells such as granulocytes.

Based on the spatial domain identification results of SpatialCOC, we analyzed each category of marker gene and marker protein:

- Multimodal biomarkers of GCs: Marker proteins: CD3, CD19, IgD; marker gene: *mt-Co2*. The protein CD3 specifically identifies T helper cells that are critical for activating B cells, whereas CD19 and IgD mark naïve and germinal center B cells. These markers collectively represent the essential T cell–B cell collaboration that drives antibody production in the GC. Complementing this protein signature, the elevated expression of the *mt-Co2* gene in both T and B cells reflects the heightened mitochondrial activity necessary to support the rapid proliferation and differentiation of lymphocytes during the germinal

center reaction.

- Multimodal biomarkers of MZs: We identified CD169 (Siglec-1) as a key protein biomarker for the marginal zones. CD169 serves as a definitive marker for a specialized subset of antigen-presenting macrophages residing in the MZ. These CD169^{+} macrophages are strategically positioned to capture blood-borne antigens and directly interact with both B cells and CD4^{+} helper T cells, playing an indispensable role in the initiation of early immune responses. The specific localization of CD169 to this compartment highlights the functional specialization of the marginal zone as a critical interface between circulation and the adaptive immune system.
- Multimodal biomarkers of Red Pulp: We identified distinct macrophage populations marked by CD68, F4–80, and CD163 as core protein biomarkers. CD68 serves as a pan-macrophage lysosomal marker, while CD163 and F4–80 collectively define red pulp macrophages specialized in hemoglobin scavenging and iron recycling. Complementing this protein signature, the high expression of the *Hbb-bt* gene in CD163^{+} and F4–80^{+} macrophages highlights their erythrophagocytic activity and intimate functional relationship with red blood cells, reflecting the essential role of red pulp macrophages in systemic iron homeostasis and the clearance of senescent erythrocytes.

(3) Mouse thymus dataset

The mouse thymus can divide into three main compartments: the Cortex, the Medulla, and the Connective Tissue[14]:

- Cortex: This is the outer region of the thymus, characterized by a dense packing of immature thymocytes and stromal cells.
- Medulla: The inner region of the thymus, with a lower cell density, is

where more mature thymocytes and stromal cells reside.

- Connective Tissue: It constitutes the structural scaffold of the thymus, comprising an outer fibrous capsule and inward trabeculae that subdivide the parenchyma into incomplete lobules.

Based on the spatial domain identification results of SpatialCOC, we analyzed each category of marker gene and marker protein:

- Multimodal Biomarkers of Cortex: Marker proteins: CD44, CD11c; Marker gene: *H2-K1*, *Rplp1*. CD44 is widely expressed on immature thymocytes and participates in their migration and proliferation, while CD11c identifies dendritic cells involved in antigen presentation. These proteins collectively delineate the cortical region where T cell development and positive selection occur. Complementing this, *H2-K1* encodes an MHC class I molecule essential for CD8+ T cell selection, and *Rplp1* demonstrates enhanced ribosomal activity required to support the rapid proliferation of thymocytes in this region.
- Multimodal Biomarkers of Medulla: Marker proteins: IgG2a (Isotype Control), CD27. CD27 serves as a key marker of mature single-positive T cells in the medulla, indicating the final stage of thymocyte maturation. Together with the IgG2a control that helps assess background signal, these markers characterize the medullary environment where negative selection and T cell emigration take place.
- Multimodal Biomarkers of Connective Tissue: Marker protein: CD68. CD68 is strongly expressed by macrophages residing in the connective tissue framework, particularly within the capsular and trabecular areas. Its presence underscores the role of connective tissue not only in structural support but also in maintaining thymic homeostasis through clearance of apoptotic thymocytes and immune regulation.

6. Simulations

(1) Simulated data for different spatial patterns

To evaluate the performance of individual models in SpatialCOC, we generated simulated datasets representing four spatial patterns with varying complexity. These spatial structures reflect common biological tissue organizations, either individually or as combinations. The four spatial patterns include quadrant structure, stripe structure, arc structure, and layered structure.

We first generate coordinate values using the numpy.linspace() function with parameters start=0, stop=60 and num=20:

$$x_i = y_i = a + i \cdot \frac{b-a}{n-1}, \; for \; i = 0,1,\ldots,19 \tag{10}$$

where:

- a is the starting value of the sequence and set to 0,
- b is the ending value of the sequence and set to 60,
- n is the number of elements in the sequence and set to 20.

This process generates 400 coordinates, corresponding to the number of spots in a single batch. We store these values in a 400x2 coordinate matrix. Since the samples are sorted based on their categories, we can simply rearrange the coordinate matrix to allocate spots from different categories into distinct spatial regions.

Quadrant pattern. Firstly, we determine the center point of the coordinate matrix at $(30,30)$, and divide the entire space into four quadrants based on this center point. By sorting and arranging coordinates according to their quadrant number, we assign spots of different cell types to distinct quadrants to form a clear regional segmentation.

Stripe pattern. We sort the Y-axis coordinates in ascending order and divide them into four stripe regions along the Y-axis. This results in a vertically striped spatial pattern, where different cell types are arranged in sequential horizontal layers.

Arc pattern. To construct an arc-shaped spatial pattern, we calculate the Euclidean distance of each coordinate from the origin and sort the coordinates accordingly. This process groups the space into multiple concentric arcs, where each level represents a distinct distance interval from the origin.

Layered pattern. Similar to the Arc Structure, we calculate the Euclidean distance of each coordinate from the geometric center $\left(30,30\right)$ and sort these distances by their distances. This approach forms a nested layered organization where cells are distributed in spatial hierarchies based on their proximity to the center.

(2) Simulated data for different noise combinations and levels

Spatial multi-omics technologies enable the simultaneous capture of multiple molecule modalities within a single tissue slice, offering a comprehensive molecular perspective on tissue structure and composition. However, the process of capturing one modality can sometimes compromise the molecular integrity required of another, reducing its sensitivity in subsequent rounds of molecular sequencing or imaging[15]. Moreover, some modalities may experience “dropout” events[16].

To simulate the real-world adverse effect on molecular integrity and assess the robustness of SpatialCOC under noisy conditions, we introduce three types of noise to the real datasets: Gaussian noise, pepper noise (dropout events), and mixed noise (combination of Gaussian and pepper noise).

Gaussian noise simulation. To simulate measurement errors and random fluctuations in real biological signals, we introduce Gaussian noise into the spatial multi-omics data. For a dataset with modalities, let

- $\mathrm{X}_1 \in R^{n\times m_1}$ and $\mathrm{X}_2 \in R^{n\times m_2}$, representing the original matrices for the two modalities,
- n be the number of measured spots,

- m_1 and m_2 be the number of features in each modality.

We add Gaussian noise as follows:

$$\begin{aligned} x_{ij}^{noised} &= x_{ij} + \mathcal{N}(\mu_1, \sigma_1^2),\ x_{ij} \in X_1 \\ x_{ij}^{noised} &= x_{ij} + \mathcal{N}(\mu_2, \sigma_2^2),\ x_{ij} \in X_2 \end{aligned} \tag{11}$$

where:

- $\mathcal{N}(\mu_1, \sigma_1^2)$ and $\mathcal{N}(\mu_2, \sigma_2^2)$ represent Gaussian distributions with given mean and variance,
- x_{ij} represents the original expression data of the location (i, j),
- x_{ij}^{noised} represents the noisy data of the location (i, j),
- To ensure the non-negativity of the data, we set $x_{ij}^{noised} = 1e-10, if\ x_{ij}^{noised} < 0$.

Pepper noise simulation ("dropout" events). To simulate "dropout" events, we introduce pepper noise using a Boolean mask:

$$x_{ij}^{noised} = x_{ij} \times mask_{ij}^{dropout} \tag{12}$$

where $mask_{ij}^{dropout}$ is a binary dropout mask, defined as:

$$mask_{ij}^{dropout} = \begin{cases} 0 & with\ probability\ \alpha \\ 1 & with\ probability\ 1-\alpha \end{cases} \tag{13}$$

where the dropout rate α controls the proportion of missing values in the dataset.

(3) Mixed noise simulation

To assess SpatialCOC's resilience under realistic noise conditions, we combine Gaussian noise and pepper noise to simulate the compound effects of measurement errors and dropout events in biological data.

7. The datasets used for evaluation

A robust evaluation of SpatialCOC must concern the inherent spatial structural

heterogeneity and the authentic technical noise found in real tissues.

(1) Spatial characteristics analyses

We have categorized and summarized the complex spatial patterns in spatial multi-omics. Our simulated spatial patterns and the selection of real-tissue samples are highly diverse, comprehensively covering major patterns such as smooth gradients, sharp boundaries, hierarchical structures, and complex regions (Supplementary Fig. 15).

- Smooth gradients: Cell types or gene expression can form smooth continuous gradients within tissues, for instance, the gradual changes in gene expression observed across the cerebral cortex[17].
- Sharp boundaries: At the interfaces between tissue domains, abrupt transitions in cellular composition or gene expression often occur, forming sharp anatomical boundaries, such as the demarcation between the hippocampus and the cortex[12].
- Hierarchical structures: Within tissues and organs, such as pancreatic germinal centers[13] or thymic lobules, the spatial arrangement of cells often exhibits clear hierarchical or zonal organization.
- Complex regions: Examples include organ primordia during mouse embryonic development, such as the developing liver and heart in the embryo[18].

In the simulated dataset, the spatial structures of various real tissues have been simulated except for the complex regions. Moreover, the selected real datasets all cover the following types of spatial structures:

- Mouse brain dataset with mixed smooth and boundary regions. The spatial structure of this dataset demonstrates two typical characteristics - it contains both continuous regions with smooth transitions in cell states (e.g., in cortical areas) and regions with clear tissue boundaries (e.g., the hippocampal-cortical junction).

- Mouse spleen dataset with a central–peripheral pattern. Through combined histopathological image analysis, we found that this dataset exhibits a typical central-peripheral distribution pattern, containing both central cell aggregation areas (showing non-smooth distribution) and peripheral smooth transition regions.
- Mouse thymus dataset with a hierarchical structure. This dataset displays a distinct spatial hierarchy, enabling clear differentiation between the central medullary area and peripheral cortical region. Particularly noteworthy is that SpatialCOC successfully identified the connective tissue transition zone in the central area, demonstrating its capability to capture subtle structural variations.

(2) Noise interference analysis

The spatial multi-omics data often suffer from the following noise interference (Supplementary Fig. 15):

- Measurement errors introduced by limitations in spatial resolution.
- Molecular integrity due to fluctuations in molecular capture efficiency (e.g., gene dropout events[16]).
- Batch effects[19] that are unavoidable across different experimental batches or sample preparation procedures.

In our simulated data, we modeled measurement errors by introducing Gaussian noise, and represented molecular integrity degradation using pepper noise. Furthermore, we assessed the method's performance under real-world noise scenarios—including background noise and batch effects—by analyzing three slices from the mouse thymus dataset (Supplementary Fig. 15).

Supplementary Figures

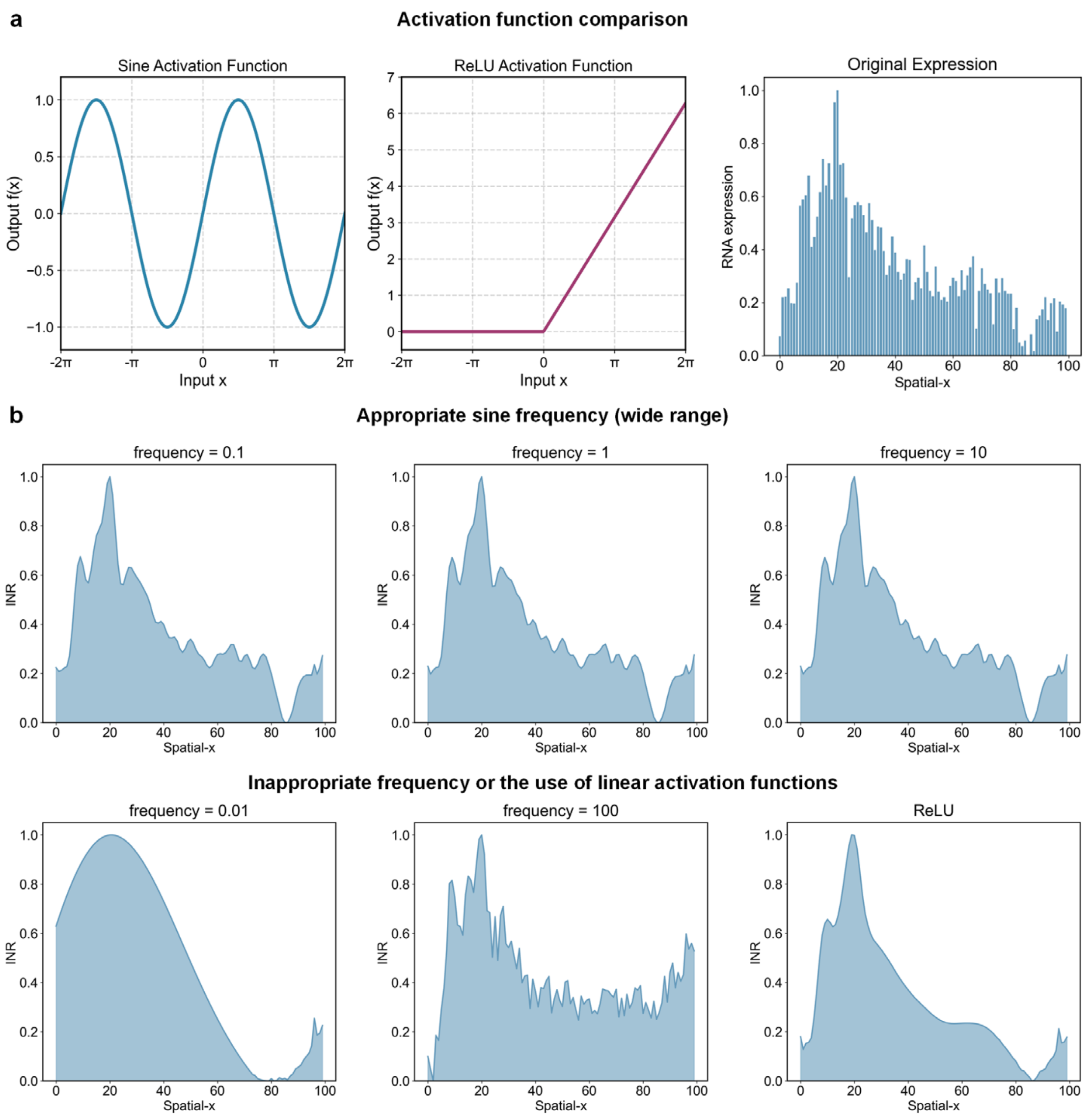

Supplementary Fig. 1 | Comparison of different activation functions used in SCM module. a Reconstruction performance was compared between sinusoidal activation functions (across frequencies) and ReLU (linear-type), benchmarked on RNA modality distributions along the x-axis of the mouse brain ATAC slice. **b** The reconstruction performance of different activation functions. An appropriate frequency (ranging from 0.1 to 10, a wide range) can robustly reconstruct the continuous distribution of omics. In contrast, extreme frequencies or the use of a purely linear activation function all fail to reconstruct the original signal accurately. Source data are provided as a Source Data file.

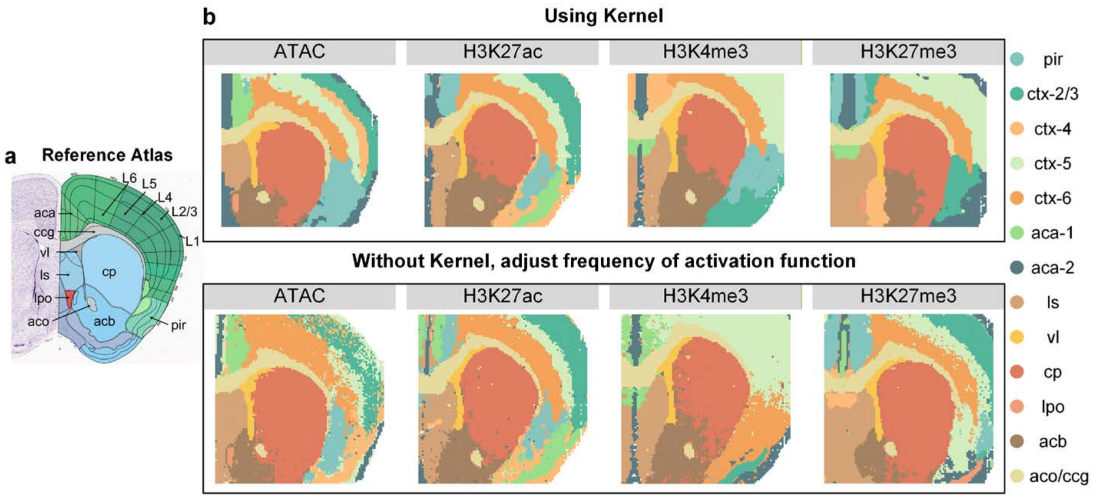

Supplementary Fig. 2 | Effect of kernel removal. a Reference atlas. The annotation of P56 mouse brain coronal sections from the Allen Mouse Brain Atlas (mouse.brain-map.org) was used as the reference atlas. **b** Comparison of SpatialCOC with and without the kernel. The kernel function is provided as an optional parameter, and its effect was evaluated across four slices of the mouse brain dataset. Applying the kernel enhances local smoothness; after removing it and suitably adjusting the sine frequency, the results remain stable, with only local smoothness being reduced. Source data are provided as a Source Data file.

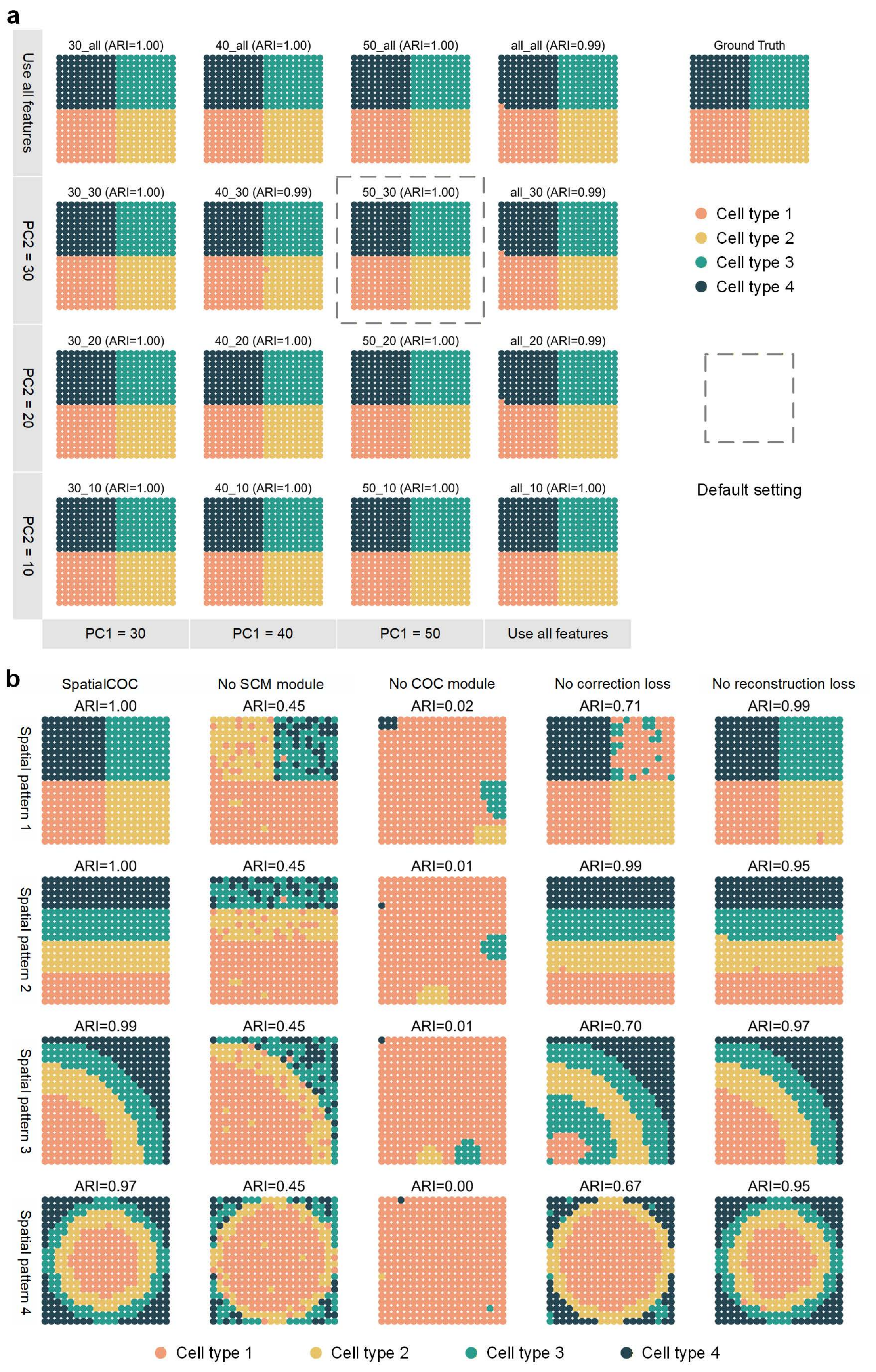

Supplementary Fig. 3 | Parameter sensitivity analyses and ablation experiments on HLN-augmented datasets. a Sensitivity analyses of input

dimensionality. To evaluate the robustness of SpatialCOC to varying input dimensions, we tested its performance using different numbers of principal components (10, 20, 30) and the original data. Performance across all conditions was quantified using the adjusted rand index (ARI), which highlighted its ability to maintain consistent results across different dimensions ($n = 400$ spots for each condition). **b** Ablation experiments. To assess the contribution of each component in SpatialCOC, we conducted ablation experiments on HLN-augmented datasets across spatial patterns. We independently removed the following components: the Spatial Continuous Mapping (SCM) module, the Cross-Omics Correction (COC) module, and the individual loss functions (CCA Loss and Reconstruction Loss). We quantified the performance of each condition using the ARI, demonstrating the necessity of all components for achieving effective multimodal fusion and accurate spatial domain identification. Source data are provided as a Source Data file.

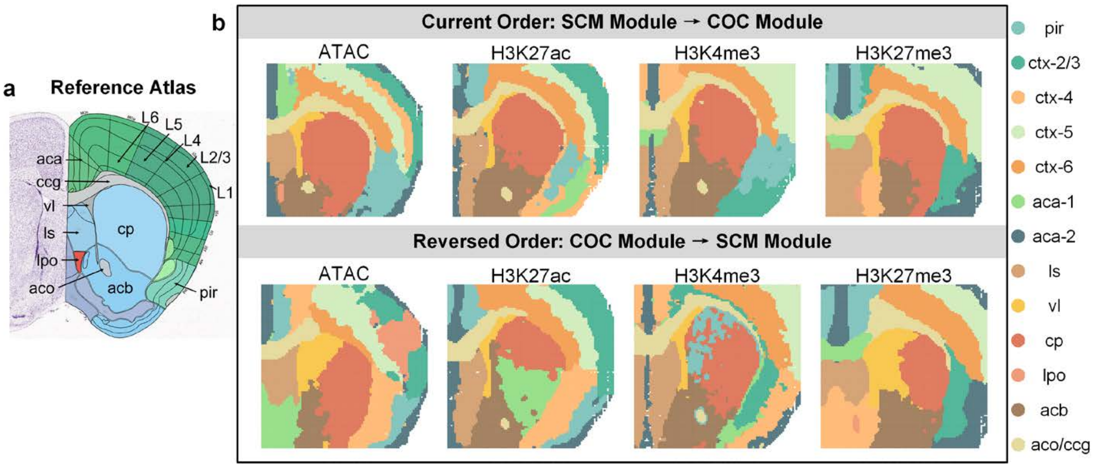

Supplementary Fig. 4 | Inter-module logic verification. a Reference atlas. The annotation of P56 mouse brain coronal sections from the Allen Mouse Brain Atlas (mouse.brain-map.org) was used as the reference atlas. **b** Logic verification for the two modules used in SpatialCOC. The logical connection between the spatial continuous mapping (SCM) module and the cross-omics correction (COC) module was evaluated across four slices of the mouse brain dataset. Smooth and biologically meaningful results were obtained with the current order (SCM followed by COC). In contrast, reversing the order (COC followed by SCM) yielded comparable smoothness but resulted in a loss of biological meaning. Source data are provided as a Source Data file.

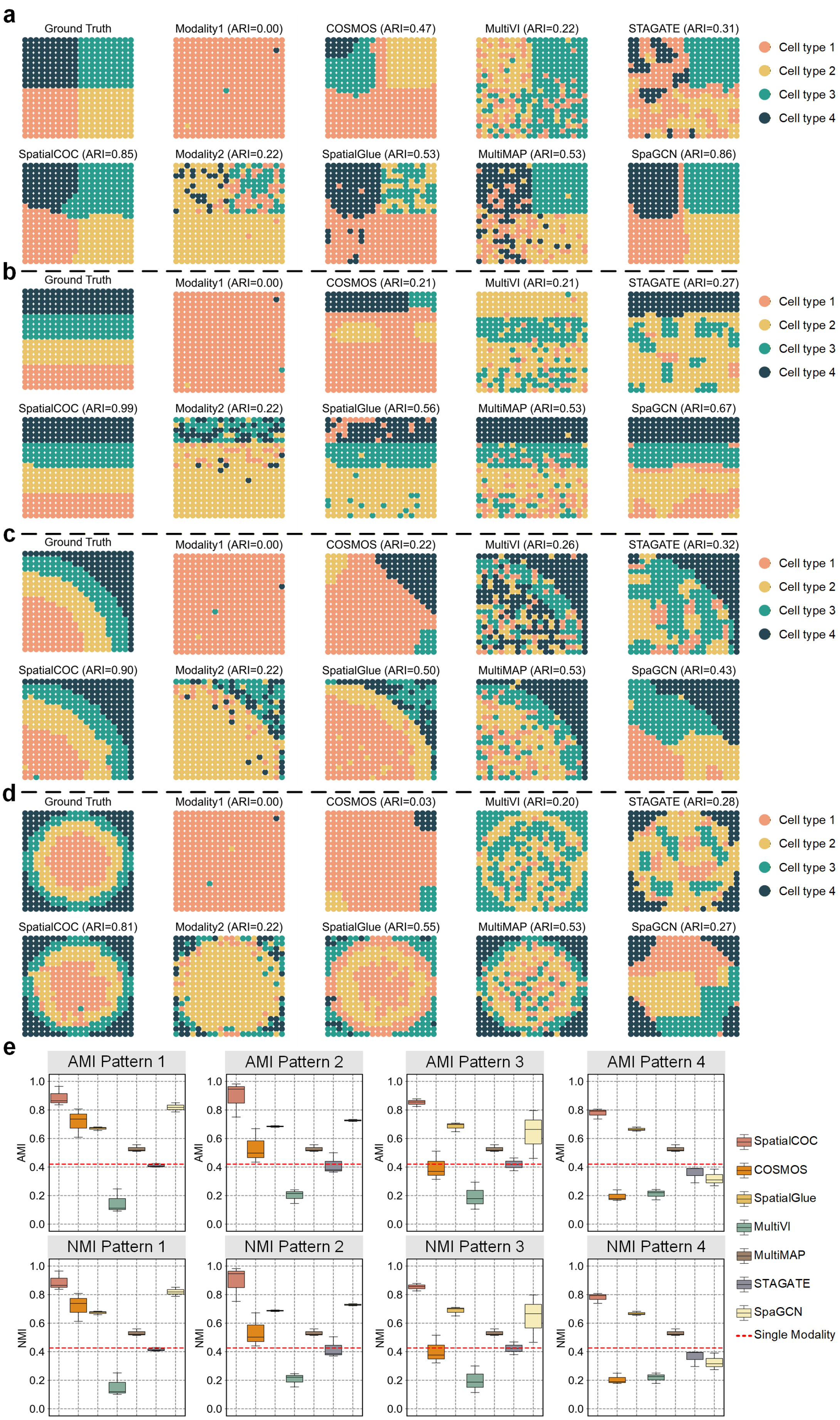
a
Ground Truth
Modality1 (ARI=0.00)
COSMOS (ARI=0.47)
MultiVI (ARI=0.22)
STAGATE (ARI=0.31)
SpatialCOC (ARI=0.85)
Modality2 (ARI=0.22)
SpatialGlue (ARI=0.53)
MultiMAP (ARI=0.53)
SpaGCN (ARI=0.86)
Cell type 1
Cell type 2
Cell type 3
Cell type 4
b
Ground Truth
Modality1 (ARI=0.00)
COSMOS (ARI=0.21)
MultiVI (ARI=0.21)
STAGATE (ARI=0.27)
SpatialCOC (ARI=0.99)
Modality2 (ARI=0.22)
SpatialGlue (ARI=0.56)
MultiMAP (ARI=0.53)
SpaGCN (ARI=0.67)
c
Ground Truth
Modality1 (ARI=0.00)
COSMOS (ARI=0.22)
MultiVI (ARI=0.26)
STAGATE (ARI=0.32)
SpatialCOC (ARI=0.90)
Modality2 (ARI=0.22)
SpatialGlue (ARI=0.50)
MultiMAP (ARI=0.53)
SpaGCN (ARI=0.43)
d
Ground Truth
Modality1 (ARI=0.00)
COSMOS (ARI=0.03)
MultiVI (ARI=0.20)
STAGATE (ARI=0.28)
SpatialCOC (ARI=0.81)
Modality2 (ARI=0.22)
SpatialGlue (ARI=0.55)
MultiMAP (ARI=0.53)
SpaGCN (ARI=0.27)
e
AMI Pattern 1
AMI Pattern 2
AMI Pattern 3
AMI Pattern 4
NMI Pattern 1
NMI Pattern 2
NMI Pattern 3
NMI Pattern 4
AMI
NMI
0.0
0.2
0.4
0.6
0.8
1.0
SpatialCOC
COSMOS
SpatialGlue
MultiVI
MultiMAP
STAGATE
SpaGCN
Single Modality

Supplementary Fig. 5 | Benchmarking on HLN-augmented datasets across four spatial patterns. a-d Clustering performance across four spatial patterns of the first replicated experiment. For each of the four spatial patterns, the figure presents ground truth labels, clustering results using mono-omics, and the performance of various integration methods. These patterns represent different levels of spatial complexity, modeled after real biological structures. The clustering outcomes illustrate the ability of each method to preserve spatial organization and accurately identify spatial domains, as quantified by the adjusted rand index (ARI) ($n = 400$ spots). **e** Quantitative evaluation using the adjusted mutual information (AMI) and the normalized mutual information (NMI). To quantitatively assess clustering performance, AMI and NMI were computed across the four spatial patterns. Box plots depict the distributions of AMI and NMI scores for seven methods, comparing their performance ($n = 3$ replicate groups for each method). In the boxplot, the center line, box limits, and whiskers denote the median, upper, and lower quartiles, and 1.5× interquartile range, respectively. The red dashed line denotes the best median ARI achieved by mono-omics clustering and serves as the reference baseline. The results demonstrate that SpatialCOC consistently outperforms other methods, achieving higher AMI and NMI scores across all spatial patterns. This highlights its effectiveness in integrating spatial multi-omics data while maintaining biologically relevant spatial patterns. Source data are provided as a Source Data file.

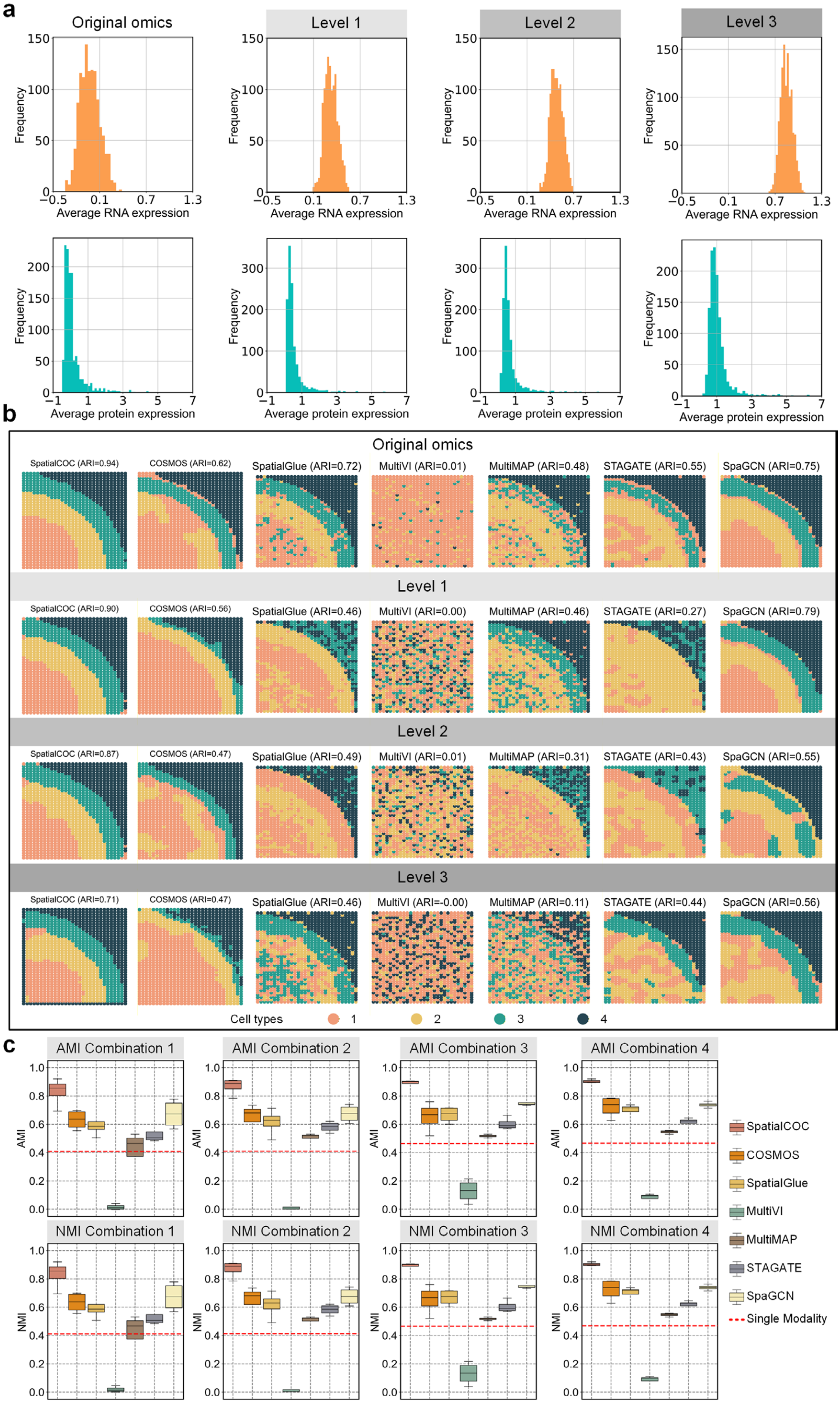

a
Original omics
Level 1
Level 2
Level 3
Frequency
Average RNA expression
Average protein expression
b
Original omics
SpatialCOC (ARI=0.94)
COSMOS (ARI=0.62)
SpatialGlue (ARI=0.72)
MultiVI (ARI=0.01)
MultiMAP (ARI=0.48)
STAGATE (ARI=0.55)
SpaGCN (ARI=0.75)
Level 1
SpatialCOC (ARI=0.90)
COSMOS (ARI=0.56)
SpatialGlue (ARI=0.46)
MultiVI (ARI=0.00)
MultiMAP (ARI=0.46)
STAGATE (ARI=0.27)
SpaGCN (ARI=0.79)
Level 2
SpatialCOC (ARI=0.87)
COSMOS (ARI=0.47)
SpatialGlue (ARI=0.49)
MultiVI (ARI=0.01)
MultiMAP (ARI=0.31)
STAGATE (ARI=0.43)
SpaGCN (ARI=0.55)
Level 3
SpatialCOC (ARI=0.71)
COSMOS (ARI=0.47)
SpatialGlue (ARI=0.46)
MultiVI (ARI=-0.00)
MultiMAP (ARI=0.11)
STAGATE (ARI=0.44)
SpaGCN (ARI=0.56)
Cell types 1 2 3 4
c
AMI Combination 1
AMI Combination 2
AMI Combination 3
AMI Combination 4
NMI Combination 1
NMI Combination 2
NMI Combination 3
NMI Combination 4
AMI
NMI
SpatialCOC
COSMOS
SpatialGlue
MultiVI
MultiMAP
STAGATE
SpaGCN
Single Modality

Supplementary Fig. 6 | Benchmarking on HLN-augmented datasets with Gaussian noise added to two modalities at different levels. a Effects of Gaussian noise on RNA and protein modalities. This panel presents histograms depicting the frequency distributions of original expression for RNA (using 20 bins) and protein (using 40 bins) modalities, both before and after the addition of Gaussian noise at three different levels. The noise introduces varying degrees of distortion, allowing us to assess the robustness of different spatial domain identification methods under noisy conditions. **b** Spatial domain identification performance across noise levels. The performance of seven different methods in identifying spatial domains is compared using original modality data without noise and with three increasing levels of Gaussian noise, quantified by adjusted rand index (ARI) ($n = 1200$ spots). The results illustrate how well each method retains spatial structure despite increasing noise, highlighting the robustness of SpatialCOC compared to alternative approaches. **c** Quantitative evaluation using the adjusted mutual information (AMI) and the normalized mutual information (NMI). To quantify performance, AMI and NMI were computed for each method under three levels of added noise and a no-noise baseline condition ($n = 4$ combinations). Boxplots display the AMI and NMI distributions across all seven methods. In the boxplot, the center line, box limits, and whiskers denote the median, upper, and lower quartiles, and 1.5× interquartile range, respectively. The red dashed line denotes the best median ARI achieved by mono-omics clustering and serves as the reference baseline. The results demonstrate that SpatialCOC consistently achieves superior performance, maintaining high AMI and NMI values even under significant noise. This underscores its robustness in handling noisy spatial multi-omics data while preserving biologically meaningful spatial patterns. Source data are provided as a Source Data file.

Replicated Experiment 2 of Different Spatial Patterns

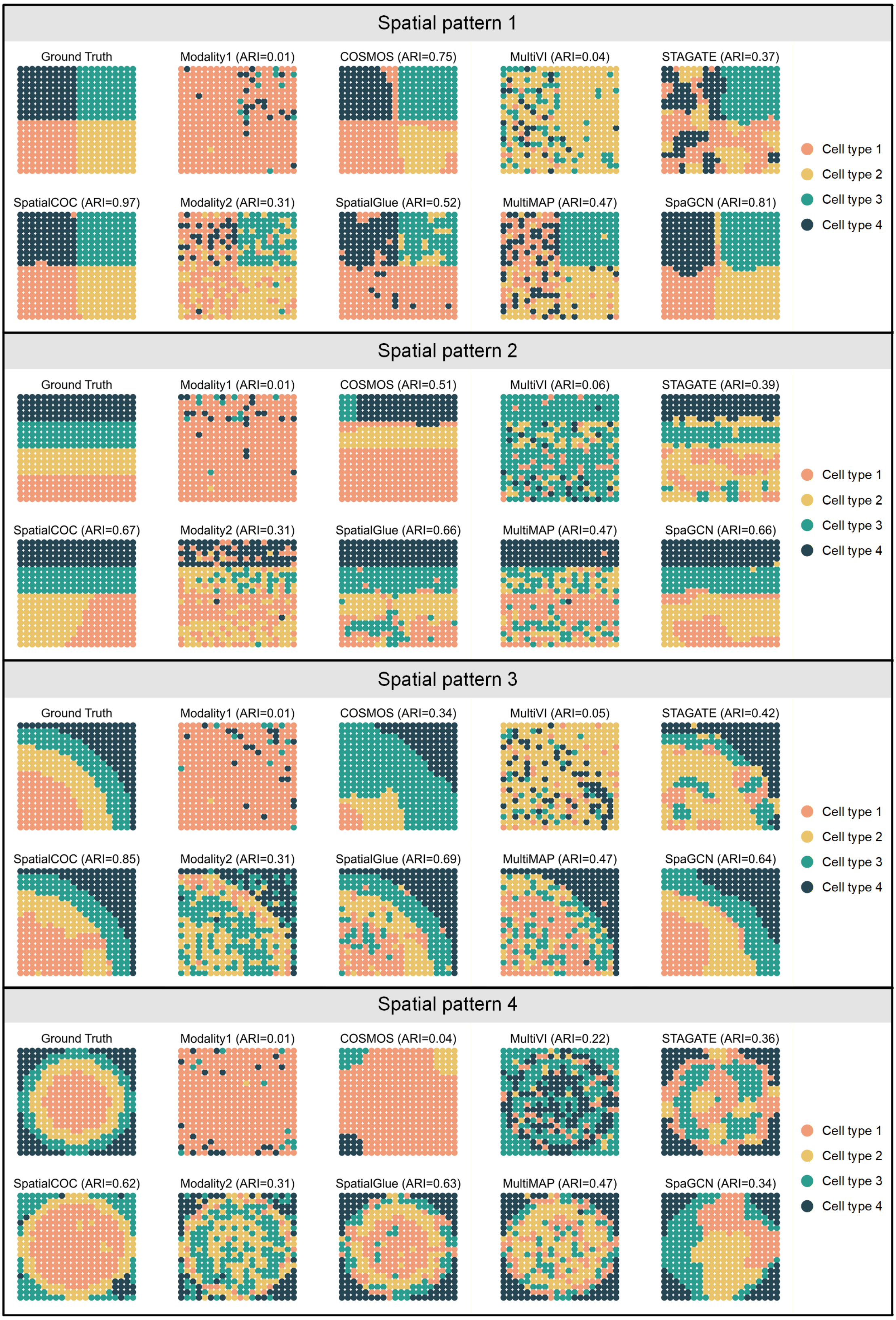

See next page

Replicated Experiment 3 of Different Spatial Patterns

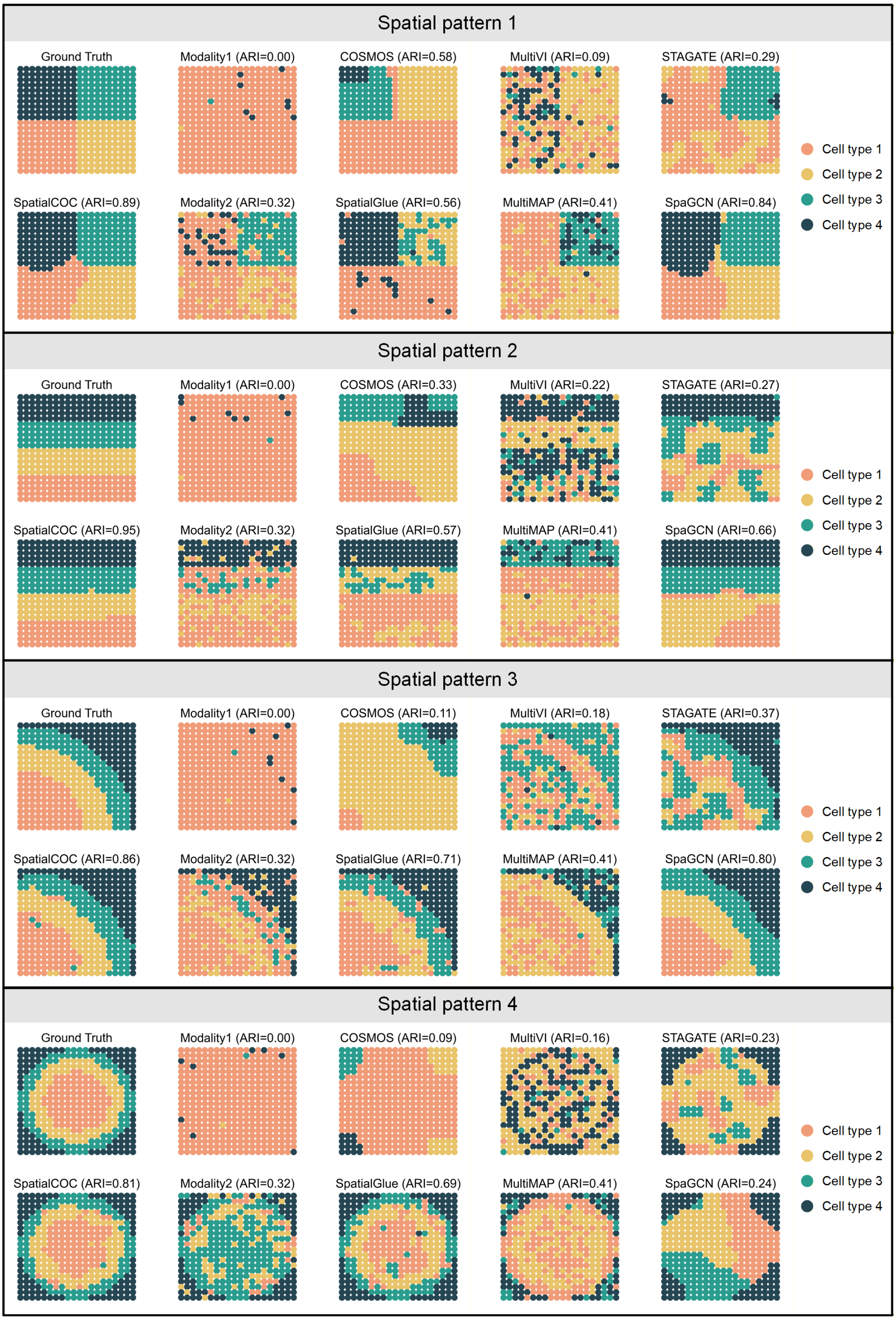

Supplementary Fig. 7 | Spatial clustering performance comparison among seven methods across four spatial patterns of replicated experiments 2

and 3. For each of the four spatial patterns, the figure presents ground truth labels, clustering results using mono-omics, and the performance of various integration methods. These patterns represent different levels of spatial complexity, modeled after real biological structures. The clustering outcomes illustrate the ability of each method to preserve spatial organization and accurately identify spatial domains, quantified by adjusted rand index (ARI) ($n = 400$ spots). Spatial Pattern 1: a simple spatial distribution of quadrant structure with well-separated regions; Spatial Pattern 2: a moderate spatial complexity of strip structure with gradual transitions between regions, forming stripe-like patterns; Spatial Pattern 3: a more intricate spatial pattern of art structure with gradual transitions between regions, exhibiting curved patterns; Spatial Pattern 4: the most complex spatial layered structure, characterized by concentric, progressively changing regions. All replicate experiments across four spatial patterns quantitively evaluated the adjusted mutual information (AMI) and the normalized mutual information (NMI) are presented in Supplementary Fig. 5e. Source data are provided as a Source Data file.

Noise Combination 2 across Different Levels

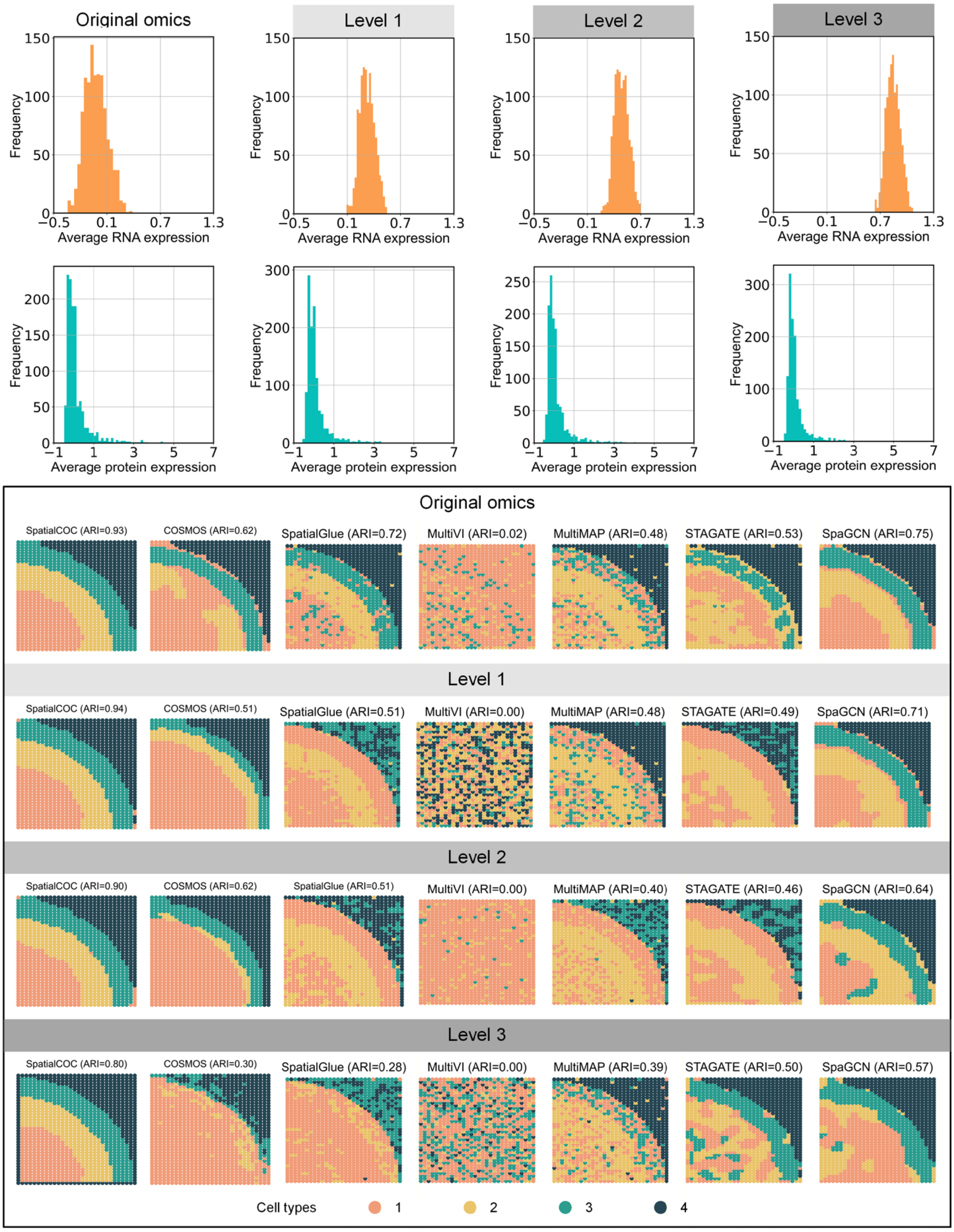

See next page

Noise Combination 3 across Different Levels

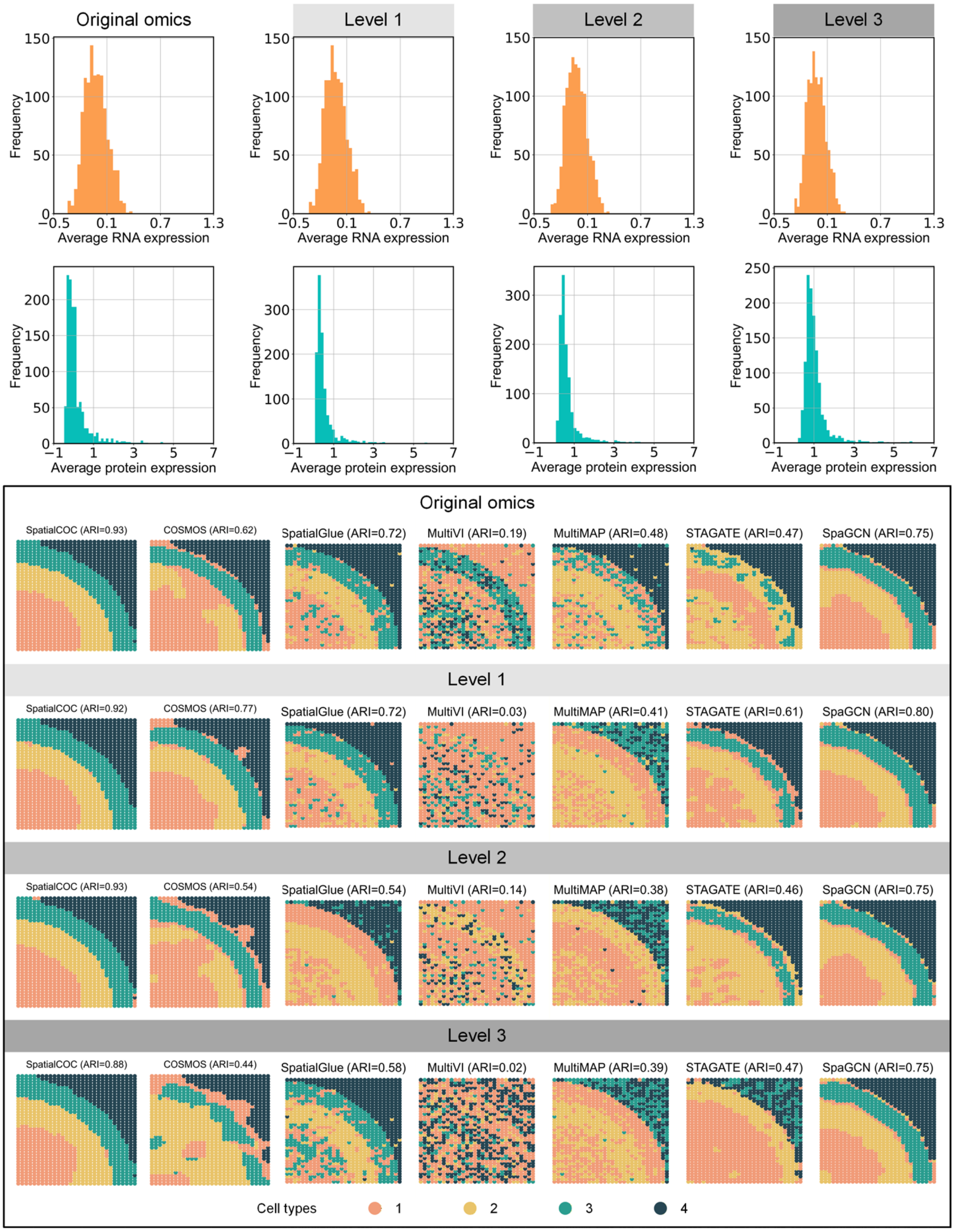

See next page

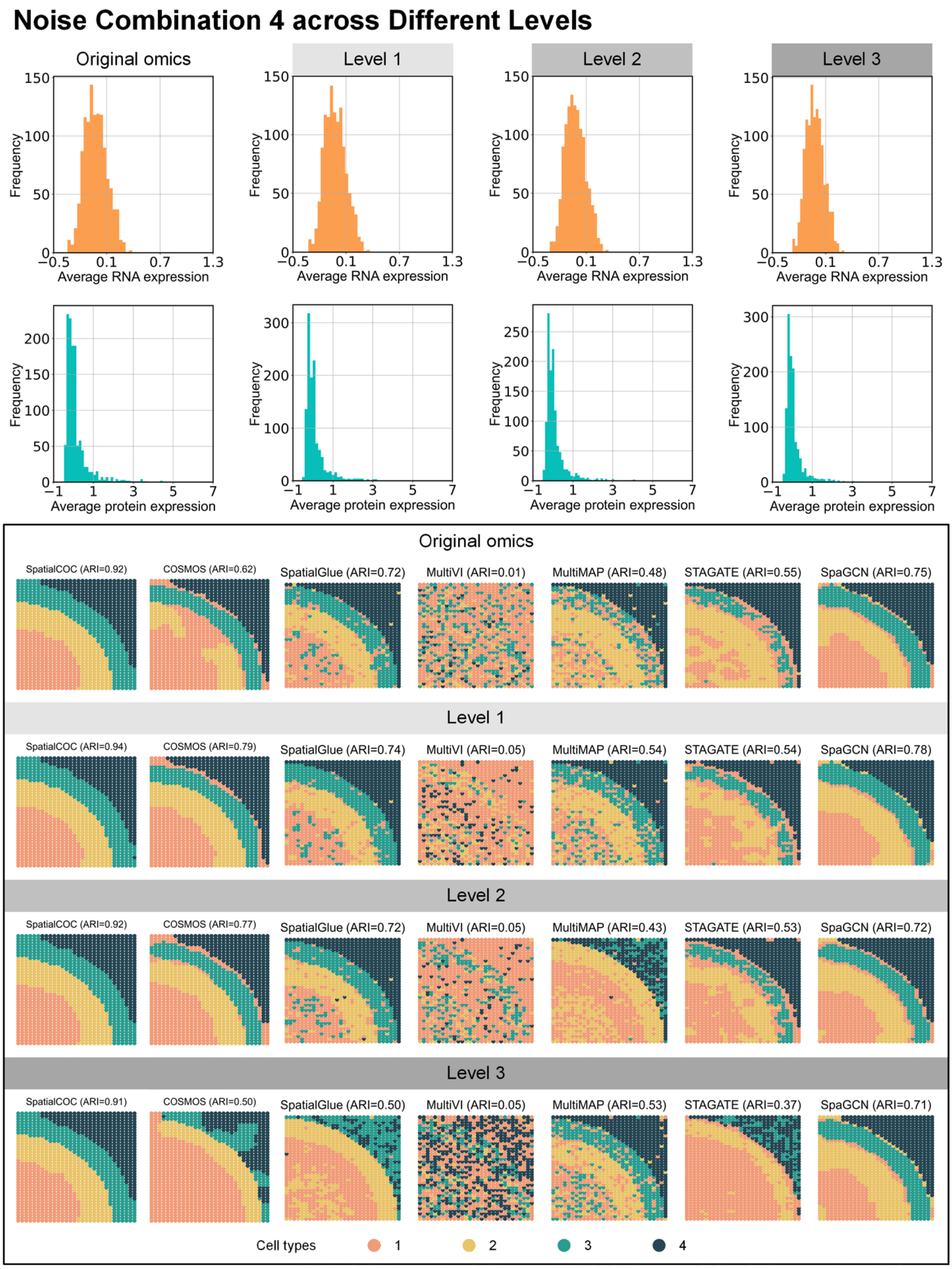

Supplementary Fig. 8 | Spatial clustering performance comparison among seven methods across four levels of noise combinations 2-4. A comparative visualization of the frequency distributions of original expression levels for both RNA (with 20 bins) and protein (with 40 bins) modalities, shown both before and after the addition of noise at three different levels. These noises reflect the different levels of noise interference of real spatial multi-omics

technologies. Noise combination 2: Gaussian noise added to RNA modality and pepper noise added to protein modality; Noise combination 3: pepper noise added to RNA modality and Gaussian noise added to protein modality; Noise combination 4: pepper noise added to both RNA and protein modalities. Furthermore, the spatial domain identification results of various integration methods were provided, quantified by adjusted rand index (ARI) ($n = 1200$ spots). All noise combinations across different noise levels quantitively evaluated using the adjusted mutual information (AMI) and the normalized mutual information (NMI) are presented in Supplementary Fig. 6c. Source data are provided as a Source Data file.

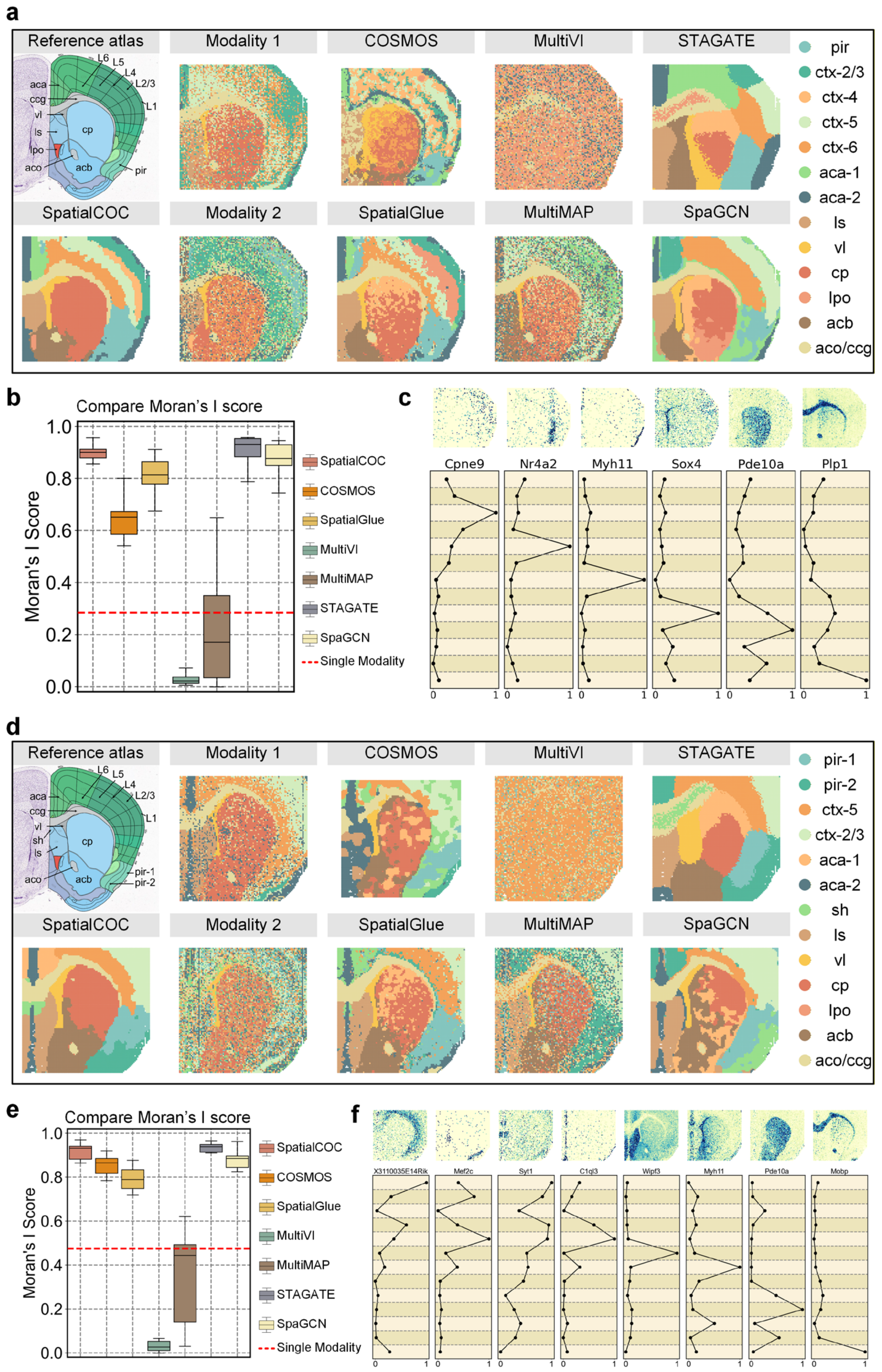

Supplementary Fig. 9 | Spatial domain identification in ATAC and H3K4me3 slices of mouse brain dataset. a Spatial domain identification in

ATAC slice. This panel presents the annotation of P56 mouse brain coronal sections (Allen Mouse Brain Atlas[28], mouse.brain-map.org), clustering results using mono-omics, and the performance of various integration methods in the ATAC slice. **b** Quantitative evaluation using Moran's I score in the ATAC slice. To quantify performance, Moran's I score was computed for each method. Boxplot displays the Moran's I score distributions across all methods ($n = 13$ clusters). In the boxplot, the center line, box limits, and whiskers denote the median, upper, and lower quartiles, and 1.5× interquartile range, respectively. The red dashed line marks the best median performance of mono-omics methods, serving as a baseline reference. **c** Marker genes identification in the ATAC slice. Based on the accurate spatial domains identified by SpatialCOC, marker genes for each cell type were calculated using scanpy package in the ATAC slice ($n = 9196$ spots). **d** Spatial domain identification in the H3K4me3 slice. This panel compares the annotation (Allen Mouse Brain Atlas, mouse.brain-map.org), mono-omics clustering results and the performance of different methods in the H3K4me3 slice. **e** Quantitative evaluation using Moran's I score in the H3K4me3 slice ($n = 12$ clusters). The interpretation of the boxplot and baseline follows the same scheme as in panel **b**. **f** Marker genes identification in the H3K4me3 slice. Scanpy package was applied to the H3K4me3 slice to extract cell-type-specific marker genes from the spatial domains accurately deciphered by SpatialCOC ($n = 9513$ spots). Source data are provided as a Source Data file.

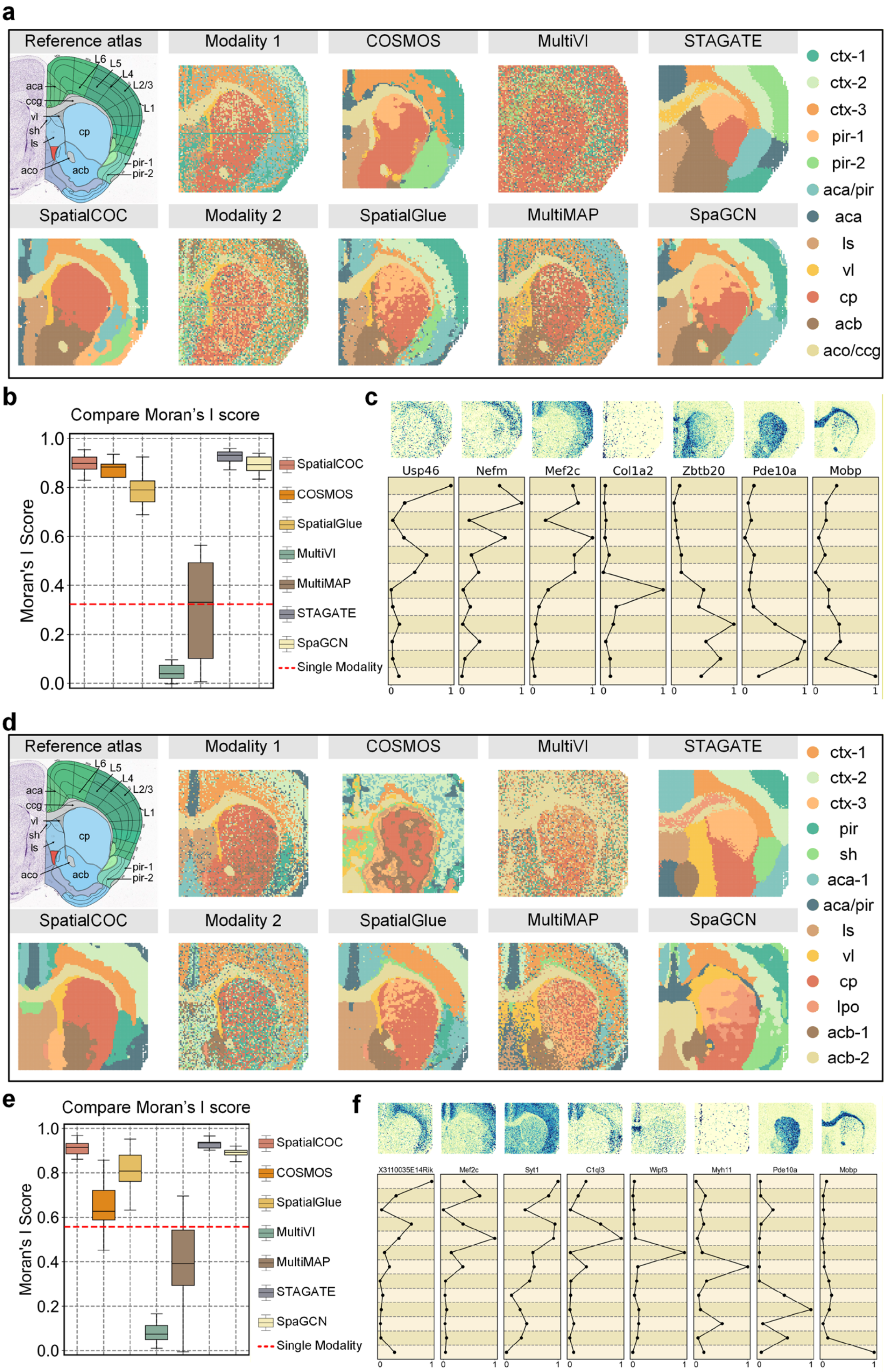

Supplementary Fig. 10 | Spatial domain identification in H3K27ac and H3K27me3 slices of mouse brain dataset. a Spatial domain identification in

H3K27ac slice. This panel presents the annotation of P56 mouse brain coronal sections (Allen Mouse Brain Atlas[28], mouse.brain-map.org), clustering results using mono-omics, and the performance of various integration methods in the H3K27ac slice. **b** Quantitively evaluation using Moran's I score in the H3K27ac slice. To quantify performance, Moran's I score was computed for each method ($n = 12$ clusters). Boxplot displays the Moran's I score distributions across all seven methods. In the boxplot, the center line, box limits, and whiskers denote the median, upper, and lower quartiles, and 1.5× interquartile range, respectively. The red dashed line marks the best median performance of mono-omics methods, serving as a baseline reference. **c** Marker genes identification in the H3K27ac slice. Based on the accurate spatial domains identified by SpatialCOC, marker genes for each cell type were calculated using scanpy package in the H3K27ac slice ($n = 9323$ spots). **d** Spatial domain identification in the H3K27me3 slice. This panel presents the annotation of P56 mouse brain coronal sections (Allen Mouse Brain Atlas, mouse.brain-map.org), clustering results using mono-omics, and the performance of various integration methods in the H3K27me3 slice. **e** Quantitively evaluation using Moran's I score in the H3K27me3 slice ($n = 13$ clusters). The interpretation of the boxplot and baseline follows the same scheme as in panel **b**. **f** Marker genes identification in H3K27me3 slice. Scanpy package was applied to the H3K27me3 slice to extract cell-type-specific marker genes from the spatial domains accurately deciphered by SpatialCOC ($n = 9732$ spots). Source data are provided as a Source Data file.

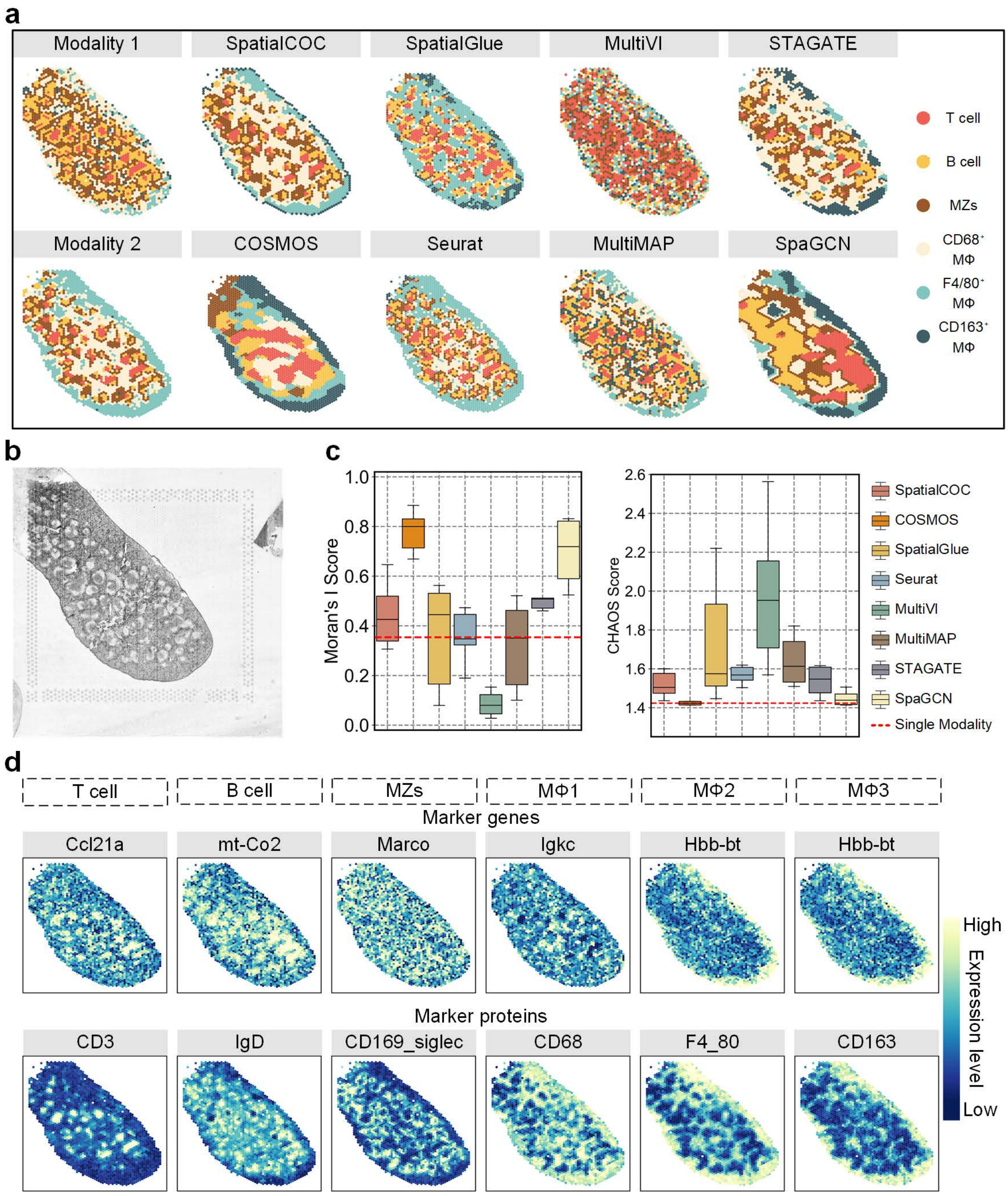

Supplementary Fig. 11 | Spatial domain identification in mouse spleen dataset replicate 1. a Spatial domain identification in replicate 1. This panel compares the mono-omics representation with the clustering performance of different methods on replicate 1 of the mouse spleen dataset. **b** Histological image of replicate 1. A histological reference image of replicate 1 is provided to validate whether the identified spatial domains correspond to known anatomical structures in the spleen. **c** Quantification of spatial structure using Moran's I and CHAOS scores for replicate 1. Moran's I score quantifies the spatial

autocorrelation, with higher values indicating better spatial structure preservation. CHAOS score assesses clustering consistency by measuring the entropy of spatial domain assignments. Box plots illustrate the distribution of these scores for each of the methods ($n = 6$ clusters). The center line, box limits, and whiskers denote the median, upper, and lower quartiles, and 1.5× interquartile range, respectively. The red dashed line represents the median value of mono-omics as a baseline reference. **d** Markers of each cluster in replicate 1. Marker genes and marker proteins are provided for further labeling each cluster. Source data are provided as a Source Data file.

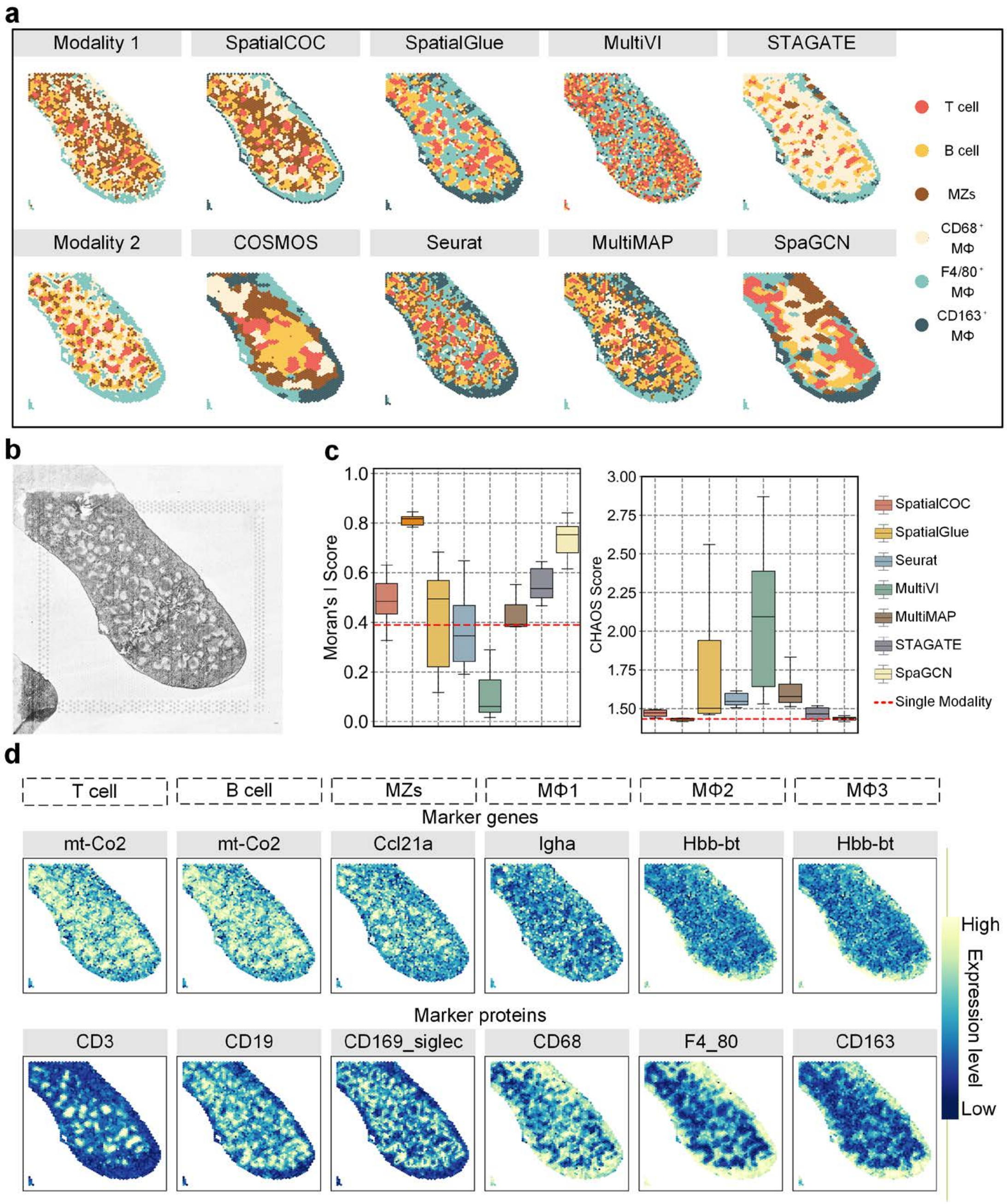

Supplementary Fig. 12 | Spatial domain identification in mouse spleen dataset replicate 2. a Spatial domain identification in replicate 2. This panel compares the mono-omics representation with the clustering performance of different methods on replicate 2 of the mouse spleen dataset. **b** Histological image of replicate 2. A histological reference image of replicate 2 is provided to validate whether the identified spatial domains correspond to known anatomical structures in the spleen. **c** Quantification of spatial structure using Moran's I and CHAOS scores for replicate 2. Moran's I score quantifies the spatial

autocorrelation, with higher values indicating better spatial structure preservation. CHAOS score assesses clustering consistency by measuring the entropy of spatial domain assignments. Box plots illustrate the distribution of these scores for each of the methods ($n = 6$ clusters). The center line, box limits, and whiskers denote the median, upper, and lower quartiles, and 1.5× interquartile range, respectively. The red dashed line represents the median value of mono-omics as a baseline reference. **d** Markers of each cluster in replicate 2. Marker genes and marker proteins are provided for further labeling each cluster. Source data are provided as a Source Data file.

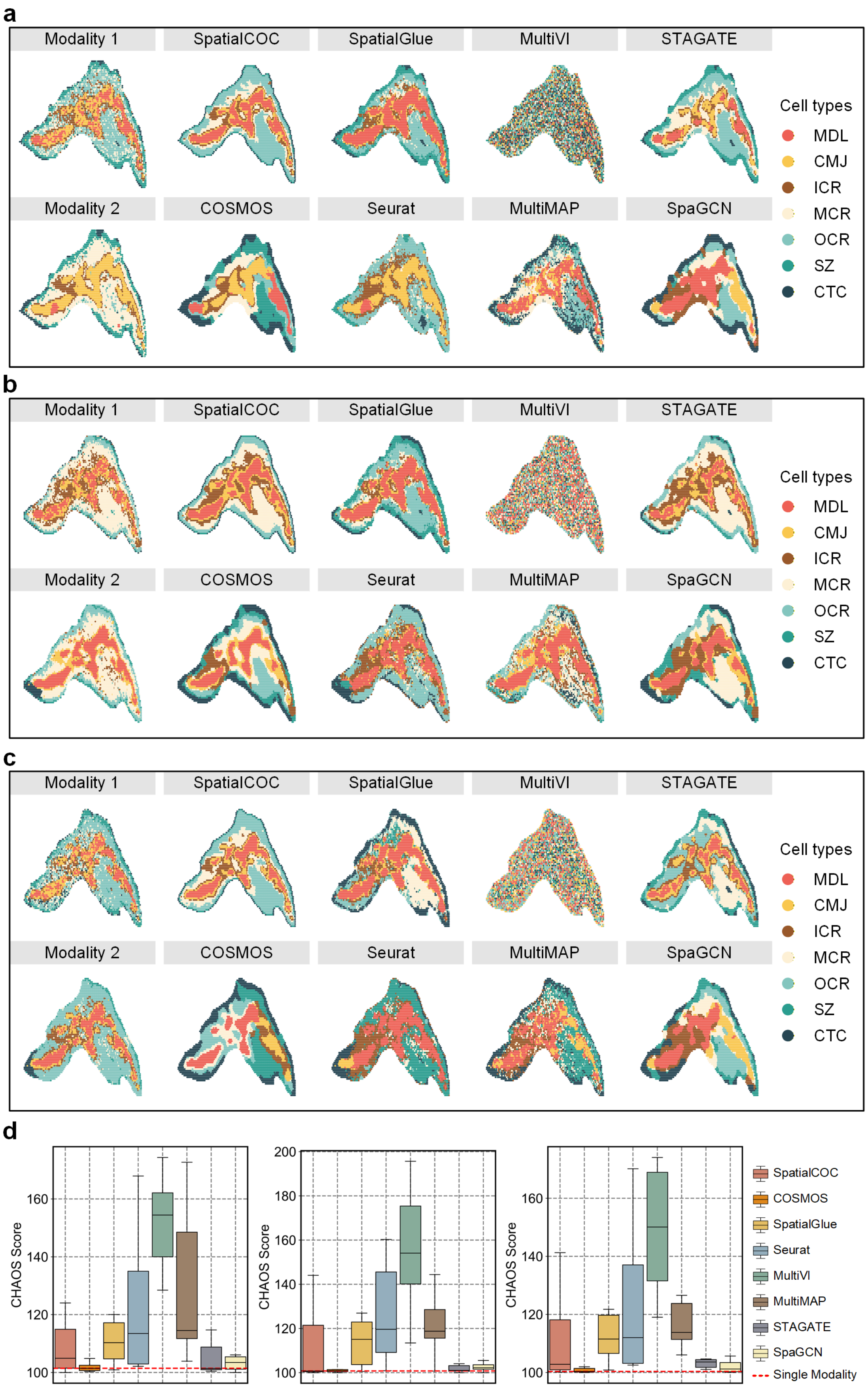

Supplementary Fig. 13 | Spatial domain identification across three

replicates of mouse thymus dataset. a-c Comparative analysis of mono-omics representation and clustering performance across three replicates of the mouse thymus dataset. Comparison of the three sections enables observation of omics data variations across different sections, as well as the consistency and robustness of spatial domain identification by different integration methods. **d** CHAOS score comparison across three replicates. The CHAOS score measures the spatial smoothness and consistency of clustering assignments, with lower values indicating more spatially coherent domain identification. Boxplots summarize the CHAOS score distributions for each of seven methods across replicates 1, 2, and 3 ($n = 7$ clusters). The center line, box limits, and whiskers represent the median, interquartile range, and 1.5× interquartile range, respectively. The red dashed line marks the median CHAOS score for mono-omics, serving as a reference baseline. Source data are provided as a Source Data file.

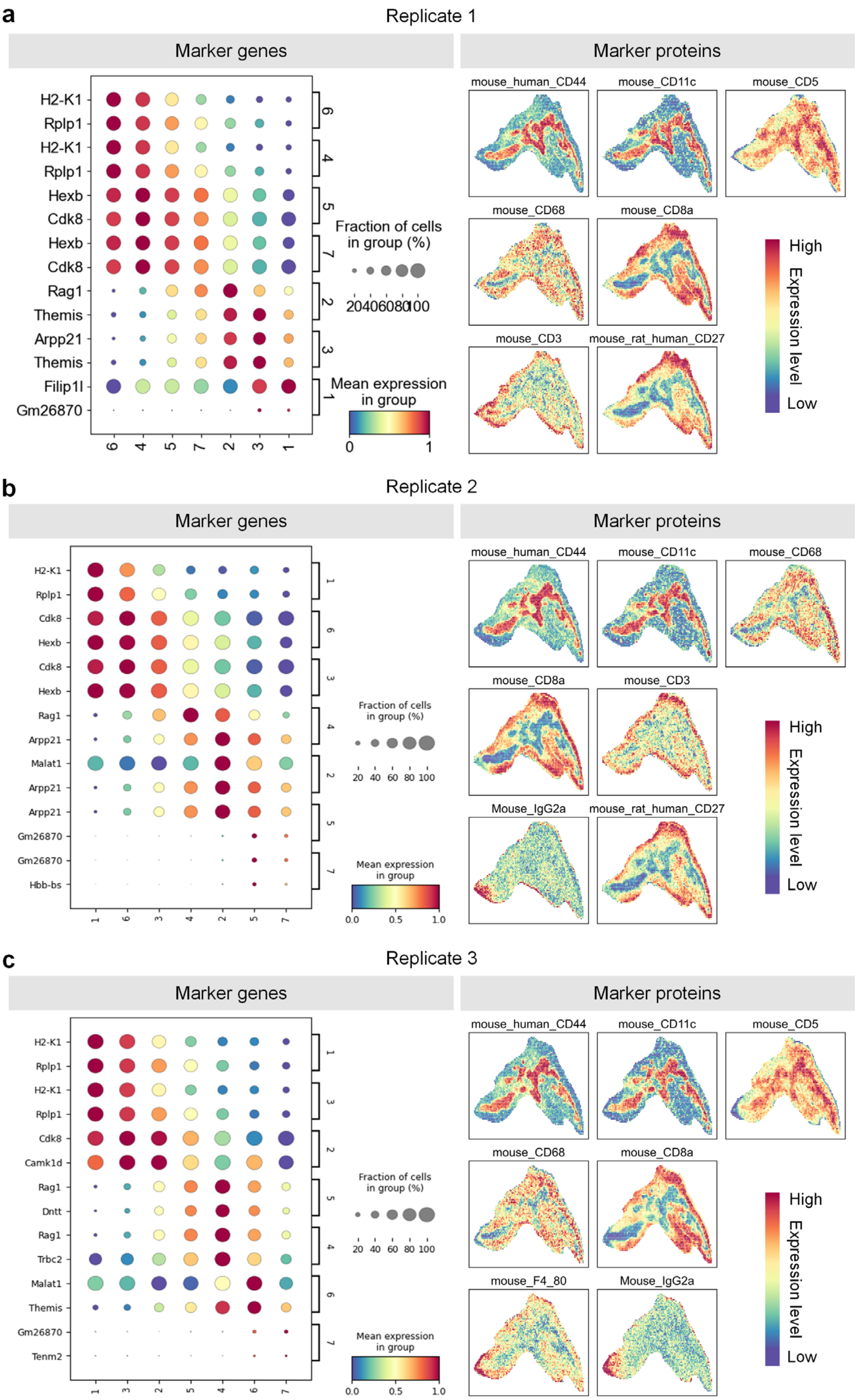
a
Replicate 1
Marker genes
Marker proteins
H2-K1
Rplp1
Hexb
Cdk8
Rag1
Themis
Arpp21
Filip1l
Gm26870
Fraction of cells in group (%)
20406080100
Mean expression in group
0
1
mouse_human_CD44
mouse_CD11c
mouse_CD5
mouse_CD68
mouse_CD8a
mouse_CD3
mouse_rat_human_CD27
High
Expression level
Low
b
Replicate 2
Marker genes
Marker proteins
H2-K1
Rplp1
Cdk8
Hexb
Rag1
Arpp21
Malat1
Gm26870
Hbb-bs
Fraction of cells in group (%)
20 40 60 80 100
Mean expression in group
0.0 0.5 1.0
mouse_human_CD44
mouse_CD11c
mouse_CD68
mouse_CD8a
mouse_CD3
Mouse_IgG2a
mouse_rat_human_CD27
High
Expression level
Low
c
Replicate 3
Marker genes
Marker proteins
H2-K1
Rplp1
Cdk8
Camk1d
Rag1
Dntt
Trbc2
Malat1
Themis
Gm26870
Tenm2
Fraction of cells in group (%)
20 40 60 80 100
Mean expression in group
0.0 0.5 1.0
mouse_human_CD44
mouse_CD11c
mouse_CD5
mouse_CD68
mouse_CD8a
mouse_F4_80
Mouse_IgG2a
High
Expression level
Low

Supplementary Fig. 14 | Validation of marker genes and proteins across three replicates of the mouse thymus dataset. In panels **a-c**, marker genes and proteins are identified to delineate each cluster across three biological replicates of the mouse thymus dataset. The marker genes (left side of each panel) reveal a clear bipartition of the thymus into two distinct regions: the central medulla and the outer cortex. Building upon this, SpatialCOC's integration strategy further resolves subpopulations within the medulla and cortex, as well as the intervening connective tissues, which are specifically annotated by their respective marker proteins. Source data are provided as a Source Data file.

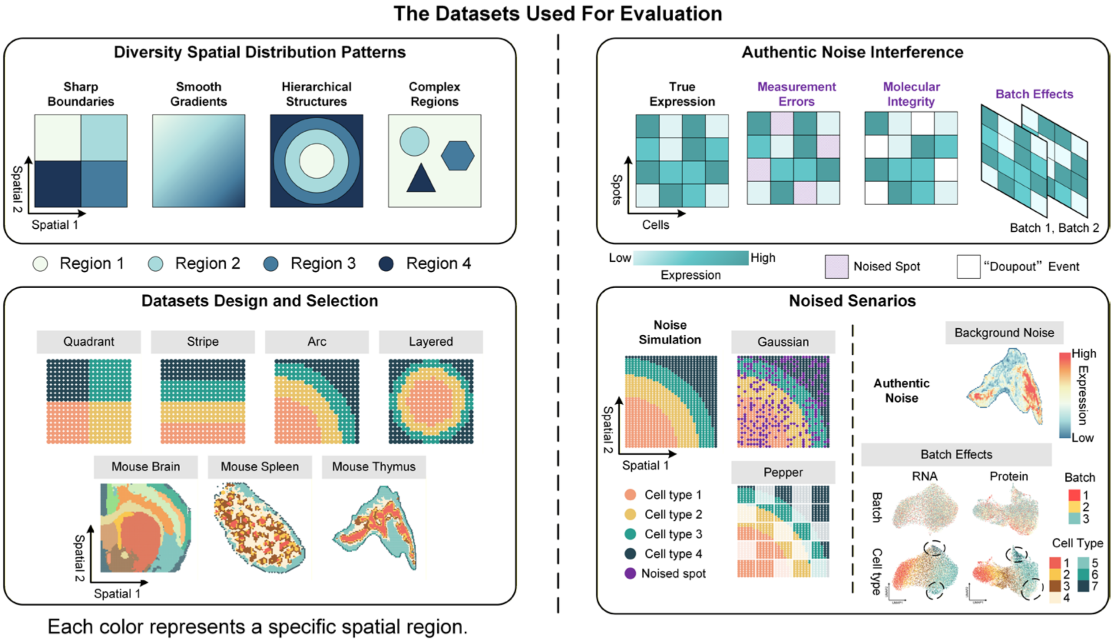

Supplementary Fig. 15 | The datasets used for evaluation. For both virtual data simulation and real-world data selection, we took into account the following diverse spatial distribution characteristics: smooth gradients, sharp boundaries, hierarchical structures, and complex regions. We also considered the following real-world noise factors: measurement errors, molecular integrity, and batch effects.

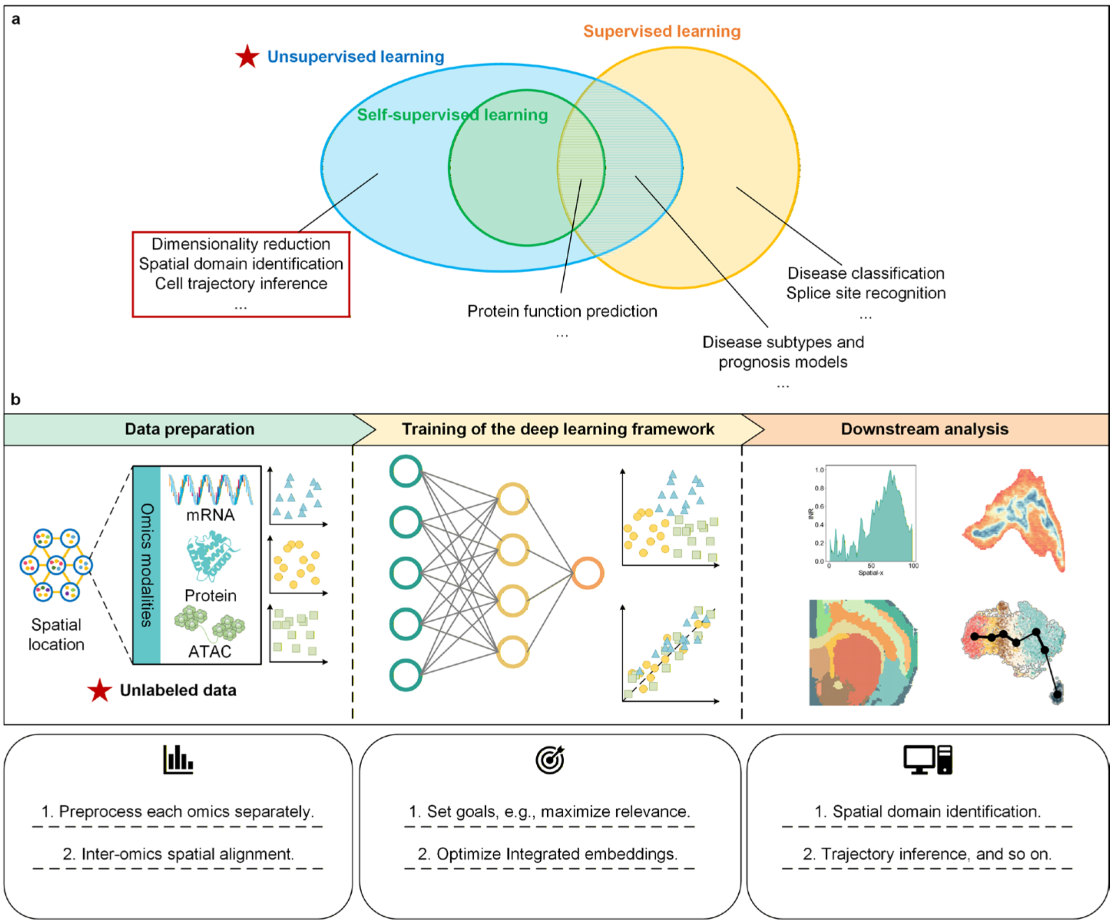

Supplementary Fig. 16 | Framework for unsupervised learning integration in SpatialCOC. a Description of the training strategy. Explanation of the relationships among unsupervised, supervised, and self-supervised learning. An unsupervised analytical framework is adopted, upon which downstream tasks—including spatial domain identification and trajectory inference—are performed. **b** SpatialCOC employs an unsupervised training strategy on observed multi-omics data through the following steps: data preparation, training of the deep learning framework, and downstream analysis. Icons used in this panel is derived from the built-in shape library of Microsoft Visio Professional 2021 (Microsoft Corporation).

Supplementary Tables

Supplementary Table 1. Description of noise added to the simulated datasets of different noise conditions. Each noise combination was applied with three levels of intensity, which increased sequentially. μ_1, μ_2 denote the mean values of two modalities, respectively.

Dataset	Noise level	omics 1			SNR (dB)	omics 2			SNR (dB)
		Mean value	Standard deviation	Dropout rate		Mean value	Standard deviation	Dropout rate	
Noise condition 1	1	μ_1	0.5	N/A	9.62	μ_2	0.5	N/A	7.42
	2	μ_1	1		4.47	μ_2	1		4.42
	3	μ_1	2		-1.51	μ_2	2		-0.36
Noise condition 2	1	μ_1	0.5	N/A	9.63	N/A		0.05	12.62
	2	μ_1	1		4.46			0.1	10.65
	3	μ_1	2		-1.5			0.2	7.08
Noise condition 3	1	N/A		0.05	13.03	μ_2	0.5	N/A	7.39
	2			0.1	10.01	μ_2	1		4.38
	3			0.2	7.01	μ_2	2		-0.37
Noise condition 4	1	N/A		0.05	13	N/A		0.05	13.69
	2			0.1	10			0.1	10.14
	3			0.2	7.01			0.2	7.38

Supplementary Table 2. Description of experimental datasets. For all the datasets used, the included omics as well as the average sparsity, the number of slices and labels, the data dimensions, and the evaluation metrics employed have been specified.

Name	Omics and sparsity	Replicate	Feature size	Evaluation metrics
HLN-augmented (diversity spatial patterns)	transcriptomics: 89.17%, proteomics: 2.34%	1	400x3,000<br>400x31	Adjusted rand index (ARI), adjusted mutual information (AMI), normalized mutual information (NMI).
		2	400x3,000<br>400x31	
		3	400x3,000<br>400x31	
		4	400x3,000<br>400x31	
HLN-augmented (graded noise combinations)	transcriptomics: 89.17%, proteomics: 2.34%	1	1,200x3,000<br>1,200x31	Adjusted rand index (ARI), adjusted mutual information (AMI), normalized mutual information (NMI).
		2	1,200x3,000<br>1,200x31	
		3	1,200x3,000<br>1,200x31	
		4	1,200x3,000<br>1,200x31	
Mouse brain	transcriptomics: 93.81%, epigenomics: 97.18%	ATAC P22	9,215x22,914<br>9,215x121,068	Moran's I score
		H3K4me3	9,548x22,731<br>9,548x35,270	
		H3K27ac	9,370x23,415<br>9,370x104,162	
		H3K27me3	9,752x25,881<br>9,752x70,470	
Mouse spleen	transcriptomics: 89.50%, proteomics: 0.41%	1	2,568x32,285<br>2,568x21	Moran's I score<br>CHAOS score
		2	2,768x32,285<br>2,768x21	
Mouse thymus	transcriptomics: 96.48%, proteomics: 0.50%	1	4,253x23,529<br>4,253x19	Adjusted rand index (ARI)<br>CHAOS score
		2	4,646x23,960<br>4,646x19	
		3	4,228x23,221<br>4,228x19	

Supplementary References

1. Moshe Leshno, Vladimir Ya. Lin, Allan Pinkus, and Shimon Schocken. Multilayer feedforward networks with a nonpolynomial activation function can approximate any function. Neural Networks, 6(6):861–867 (1993).
2. Stade, E. Fourier Coefficients and Fourier Series. In Fourier Analysis, E. Stade (Ed.) (2005).
3. Long, Y. *et al.* Deciphering spatial domains from spatial multi-omics with SpatialGlue. *Nat. Methods* **21**, 1658–1667 (2024).
4. Zhou, Y. *et al.* Cooperative integration of spatially resolved multi-omics data with COSMOS. *Nat Commun* **16**, 27 (2025).
5. Hao, Y. *et al.* Integrated analysis of multimodal single-cell data. *Cell* **184**, 3573–3587.e29 (2021).
6. Ashuach, T. *et al.* MultiVI: deep generative model for the integration of multimodal data. *Nat. Methods* **20**, 1222–1231 (2023).
7. Jain, M.S. *et al.* MultiMAP: dimensionality reduction and integration of multimodal data. *Genome Biol* **22**, 346 (2021).
8. Dong, K. & Zhang, S. Deciphering spatial domains from spatially resolved transcriptomics with an adaptive graph attention auto-encoder. *Nat Commun* **13**, 1739 (2022).
9. Hu, J. *et al.* SpaGCN: Integrating gene expression, spatial location and histology to identify spatial domains and spatially variable genes by graph convolutional network. *Nat Methods* **18**, 1342–1351 (2021).
10. Palla, G. *et al.* Squidpy: a scalable framework for spatial omics analysis. *Nat Methods* **19**, 171–178 (2022).
11. Shang, L. & Zhou, X. Spatially aware dimension reduction for spatial transcriptomics. *Nat Commun* **13**, 7203 (2022).
12. Allen Institute for Brain Science (2004). Allen Mouse Brain Atlas [dataset]. Available from mouse.brain-map.org. Allen Institute for Brain Science

(2011).

13. Ben-Chetrit *et al.* Integration of whole transcriptome spatial profiling with protein markers. *Nat Biotechnol* **41**, 788–793 (2023).
14. Liao, S., *et al.* Integrated Spatial Transcriptomic and Proteomic Analysis of Fresh Frozen Tissue Based on Stereo-seq. Preprint at https://www.biorxiv.org/content/10.1101/2023.04.28.538364v1 (2023).
15. Coleman, K., Schroeder, A. & Li, M. Unlocking the power of spatial omics with AI. Nat Methods 21, 1378–1381 (2024).
16. Qiu, P. Embracing the dropouts in single-cell RNA-seq analysis. Nat Commun 11, 1169 (2020).
17. Maynard, K.R. *et al.* Transcriptome-scale spatial gene expression in the human dorsolateral prefrontal cortex. *Nat Neurosci* **24**, 425–436 (2021).
18. Chen, A. *et al.* Spatiotemporal transcriptomic atlas of mouse organogenesis using DNA nanoball-patterned arrays. Cell, 185(10), 1777-1792 (2022).
19. Korsunsky, I. *et al.* Fast, sensitive and accurate integration of single-cell data with Harmony. *Nat Methods* **16**, 1289–1296 (2019).
