# Supplementary material for: SpatialCOC: an integrative framework for spatial continuous mapping and cross-omics correction in spatial multi-omics data
Source: Nat Commun. 2026 Apr 16;17:5268. doi: 10.1038/s41467-026-71882-2 (PMC13265925; doi:10.1038/s41467-026-71882-2)
Supplement: Supplementary file 2 — Reporting Summary [file 41467_2026_71882_MOESM2_ESM.pdf]

nature portfolio

Corresponding author(s): Kai Ye

Last updated by author(s): Feb 9, 2026

Reporting Summary

Nature Portfolio wishes to improve the reproducibility of the work that we publish. This form provides structure for consistency and transparency in reporting. For further information on Nature Portfolio policies, see our Editorial Policies and the Editorial Policy Checklist.

Statistics

For all statistical analyses, confirm that the following items are present in the figure legend, table legend, main text, or Methods section.

n/a	Confirmed	
☐	☒	The exact sample size (*n*) for each experimental group/condition, given as a discrete number and unit of measurement
☐	☒	A statement on whether measurements were taken from distinct samples or whether the same sample was measured repeatedly
☐	☒	The statistical test(s) used AND whether they are one- or two-sided<br>*Only common tests should be described solely by name; describe more complex techniques in the Methods section.*
☒	☐	A description of all covariates tested
☐	☒	A description of any assumptions or corrections, such as tests of normality and adjustment for multiple comparisons
☐	☒	A full description of the statistical parameters including central tendency (e.g. means) or other basic estimates (e.g. regression coefficient) AND variation (e.g. standard deviation) or associated estimates of uncertainty (e.g. confidence intervals)
☐	☒	For null hypothesis testing, the test statistic (e.g. *F*, *t*, *r*) with confidence intervals, effect sizes, degrees of freedom and *P* value noted<br>*Give P values as exact values whenever suitable.*
☒	☐	For Bayesian analysis, information on the choice of priors and Markov chain Monte Carlo settings
☐	☒	For hierarchical and complex designs, identification of the appropriate level for tests and full reporting of outcomes
☒	☐	Estimates of effect sizes (e.g. Cohen's *d*, Pearson's *r*), indicating how they were calculated

Our web collection on statistics for biologists contains articles on many of the points above.

Software and code

Policy information about availability of computer code

Data collection	No software was used for data collection.
Data analysis	For preprocessing and visualizing spatial multi-omics data:<br>scanpy v1.9.5, (https://github.com/scverse/scanpy),<br>matplotlib v3.8.0, (https://github.com/matplotlib/matplotlib).<br>For integrating spatial multi-omics data:<br>SpatialCOC v1.0.0, (https://github.com/xjtu-omics/SpatialCOC),<br>SpatialGlue v1.5.5, (https://github.com/JinmiaoChenLab/SpatialGlue),<br>COSMOS v1.0.0, (https://github.com/Lin-Xu-lab/COSMOS),<br>Seurat WNN v4.0.0, (https://github.com/satijalab/seurat),<br>MultiVI v1.3.1, (https://github.com/scverse/scvi-tools),<br>MultiMAP v0.0.1, (https://github.com/Teichlab/MultiMAP),<br>SpaGCN v1.2.7, (https://github.com/jianhuupenn/SpaGCN),<br>STAGATE v1.0.1, (https://github.com/QIFEIDKN/STAGATE).<br>For downstream analyses:<br>anndata v0.11.1, (https://github.com/scverse/anndata),<br>rpy2 v3.5.14, (https://github.com/rpy2/rpy2).

For manuscripts utilizing custom algorithms or software that are central to the research but not yet described in published literature, software must be made available to editors and reviewers. We strongly encourage code deposition in a community repository (e.g. GitHub). See the Nature Portfolio guidelines for submitting code & software for further information.

Data

Policy information about availability of data

All manuscripts must include a data availability statement. This statement should provide the following information, where applicable:

- Accession codes, unique identifiers, or web links for publicly available datasets
- A description of any restrictions on data availability
- For clinical datasets or third party data, please ensure that the statement adheres to our policy

Raw files and counts matrix of data employed in this paper are available in raw form from their original authors. Specifically, the Mouse Brain Dataset is deposited in the Gene Expression Omnibus (GEO) with accession code GSE205055. The Mouse Spleen Dataset is available from the GEO repository (accession no. GSE198353). The Mouse Thymus Dataset can be accessed via the Zenodo repository (https://doi.org/10.5281/zenodo.7879713). The Human Lymph Node Dataset is accessible from the GEO (accession no. GSE263617). All datasets used in this paper have been uploaded to Zenodo and are freely available at https://doi.org/10.5281/zenodo.17655345.

Research involving human participants, their data, or biological material

Policy information about studies with human participants or human data. See also policy information about sex, gender (identity/presentation), and sexual orientation and race, ethnicity and racism.

Reporting on sex and gender	N.A.
Reporting on race, ethnicity, or other socially relevant groupings	N.A.
Population characteristics	N.A.
Recruitment	N.A.
Ethics oversight	N.A.

Note that full information on the approval of the study protocol must also be provided in the manuscript.

Field-specific reporting

Please select the one below that is the best fit for your research. If you are not sure, read the appropriate sections before making your selection.

- [x] Life sciences
- [ ] Behavioural & social sciences
- [ ] Ecological, evolutionary & environmental sciences

For a reference copy of the document with all sections, see nature.com/documents/nr-reporting-summary-flat.pdf

Life sciences study design

All studies must disclose on these points even when the disclosure is negative.

Sample size	We used 10 samples of real data: one sample (Human Lymph Node Dataset) was used to create simulation datasets, and the remaining nine were analyzed directly. There was no sample-size calculation. The datasets used include:<br>four samples of the Mouse Brain Dataset;<br>two samples of the Mouse Spleen Dataset;<br>three slices of the Mouse Thymus Dataset;<br>one sample of the Human Lymph Node Dataset.<br>SpatialCOC is an integrative framework that takes all spots from a single slice (sample) as input, requiring only one slice (sample) per scenario.
Data exclusions	All filtering thresholds were pre-established based on standard spatial omics preprocessing practices to remove low-quality data while preserving biological signals.<br>The gene filtering threshold was applied consistently across the Mouse Brain, Mouse Spleen, and Mouse Thymus datasets to ensure methodological uniformity and exclude genes with sporadic expression likely representing technical noise.<br>Spot-level thresholds were adapted to each technology's characteristics, reflecting technology-specific requirements rather than post-hoc adjustments.
Replication	Not applicable. Our experiments did not aim to uncover any mechanistic or intervention effects. Instead, we benchmarked our proposed methodology against competing methods using different datasets acquired with different technologies.
Randomization	Not applicable. Our experiments did not aim to uncover any mechanistic or intervention effects and hence did not require any controls.
Blinding	This study relies on spatial multi-omics data that are acquired through automated sequencing and imaging pipelines without subjective interpretation, eliminating the need for allocation concealment. Furthermore, all downstream analyses were performed using pre-established algorithms with fixed parameters, thereby removing bias in result interpretation.

Reporting for specific materials, systems and methods

We require information from authors about some types of materials, experimental systems and methods used in many studies. Here, indicate whether each material, system or method listed is relevant to your study. If you are not sure if a list item applies to your research, read the appropriate section before selecting a response.

Materials & experimental systems

n/a	Involved in the study
☒	☐ Antibodies
☒	☐ Eukaryotic cell lines
☒	☐ Palaeontology and archaeology
☒	☐ Animals and other organisms
☒	☐ Clinical data
☒	☐ Dual use research of concern
☒	☐ Plants

Methods

n/a	Involved in the study
☒	☐ ChIP-seq
☒	☐ Flow cytometry
☒	☐ MRI-based neuroimaging

Plants

Seed stocks	I have ticked "n/a" for the "Plants" section, but this part of the form did not get hidden.
Novel plant genotypes	I have ticked "n/a" for the "Plants" section, but this part of the form did not get hidden.
Authentication	I have ticked "n/a" for the "Plants" section, but this part of the form did not get hidden.
