# Supplementary material for: SpatialCOC: an integrative framework for spatial continuous mapping and cross-omics correction in spatial multi-omics data
Source: Nat Commun. 2026 Apr 16;17:5268. doi: 10.1038/s41467-026-71882-2 (PMC13265925; doi:10.1038/s41467-026-71882-2)
Supplement: Supplementary file 3 — Transparent Peer Review file [file 41467_2026_71882_MOESM3_ESM.pdf]

nature portfolio

Peer Review File

SpatialCOC: an integrative framework for spatial continuous mapping and cross-omics correction in spatial multi-omics data

Corresponding Author: Professor Kai Ye

Parts of this Peer Review File have been redacted as indicated to remove third-party material.

This file contains all reviewer reports in order by version, followed by all author rebuttals in order by version.

Version 0:

Reviewer comments:

Reviewer #1

(Remarks to the Author)
Li et al. proposed SpaKnit, a computational framework that models multi-omics data as continuous functions mapped to spatial coordinates, thereby facilitating the discovery of nonlinear correlations across modalities. By integrating spatial location with multi-omics information, SpaKnit aims to capture complex spatial patterns, enforce smoothness, and eliminate modality-specific noise. The authors demonstrated the effectiveness of their method on several simulated and public datasets, concluding that SpaKnit outperforms existing approaches. However, I have several major concerns regarding the conceptual novelty, validation strategy, and overall clarity of the manuscript.

Major issues:
1. Although the authors assert conceptual innovation, the overall methodological novelty of SpaKnit remains limited. The framework primarily comprises two modules: an Implicit Neural Representation (INR) module and a Deep Canonically Correlated Autoencoder (DCCAE) module. While the INR module has been slightly modified to suit the research goals, the DCCAE module is directly adopted from previous studies without significant innovation, which substantially weakens the manuscript's originality. Furthermore, the supposedly novel idea of modeling multi-omics data as continuous functions over spatial coordinates is used merely for modality reconstruction and, with independently trained SpaKnit components, contributes minimally during practical data analysis.
2. Despite some conceptual framework, the manuscript falls short in addressing key practical challenges, undermining the method's credibility in real-world scenarios. For instance, due to inherent differences in data formats among modalities, it is unclear how the proposed neural network is capable of reconstructing all modalities from spatial coordinates. Moreover, the framework reconstructs smoothed, rather than raw, data. The smoothing strategy, however, risks obscuring sharp transitions at spatial domain boundaries, potentially masking biologically meaningful expression changes.
3. The evaluation of the method is overly simplistic. It relies primarily on simulated data and uses spatial smoothness as the sole quantitative metric. The manuscript does not offer sufficient evaluation using real, complex datasets or more comprehensive performance metrics. Importantly, the authors' justification that their method achieves a balance—even when smoothness scores are inferior to other approaches—fails to compensate for the lack of rigorous metric design.
4. The manuscript exhibits a troubling degree of arbitrariness in its biological interpretations. The authors designate certain clusters as biologically meaningful without providing supporting evidence, such as marker gene expression or known anatomical structures. This undermines the credibility of any biological insight claimed.
5. Misalignment of methodological direction. The manuscript frames SpaKnit as a multi-omics integration tool, but it lacks comparison with state-of-the-art methods for multi-source data integration. In the context of multi-slice integration, no meaningful demonstration of batch effect removal is provided. The authors should incorporate benchmark comparisons in both domains.
6. Insufficient multi-Omics analysis. Given that SpaKnit is positioned as a multi-modal integration method, focusing solely on spatial domain identification is insufficient. The authors should provide detailed analyses across different modalities to substantiate the integrative capabilities of the method.
7. The case studies presented involve only two datasets, which is insufficient to support the method's general applicability. Additional spatial omics datasets with biologically relevant contexts should be incorporated to demonstrate the utility and

versatility of SpaKnit.

Minor issues:
1. In Figure 1, the reconstruction error of the autoencoder should be computed on data with consistent dimensions. The discrepancy between the figure and the description in the Methods section is confusing.
2. There is inconsistent notation throughout the Methods section—for example, the symbol x is used ambiguously to represent different concepts.
3. Appendix Figure 4c lacks an adequate explanation of the displayed gene expression patterns.
4. The Methods section should be expanded to describe downstream analyses, including spatial domain identification and trajectory inference strategies.
5. The English writing should be polished to improve readability.

(Remarks on code availability)
The code provides an explanatory document that elaborately introduces the presentation of each figure in the article

Reviewer #2

(Remarks to the Author)
- Summary

SpaKnit proposes a novel framework for spatial multi-omics integration by modeling modalities as continuous spatial functions and aligning them via a deep canonical correlation autoencoder. It clearly addresses some known limitations of prior methods like reliance on local graphs or linear summation. However, it is not convincing that SpaKnit really solves the core integration challenges in a way that's broadly useful or generalizable. For example, what happens when we don't have perfectly paired multi-modal data? Can it do anything in low-sample or few-shot settings? These are real bottlenecks in spatial omics today. Also, the lack of a clear training/validation/test split makes it hard to assess generalization at all.

- Strengths

1. SpaKnit treats each modality as a continuous function of space via Implicit Neural Representation (INR), enabling spatially smooth, resolution-independent reconstruction.

2. DCCAE models nonlinear correlations between modalities while explicitly removing modality-specific noise.

3. The benchmarking across multiple synthetic spatial patterns and real datasets is thorough and generally supports the method's robustness, especially under noise perturbation.

- Major concerns

1. SpaKnit is trained on paired modalities with shared spatial coordinates. But in reality, spatial transcriptomics and proteomics (e.g., Slide-seq and CODEX) are often collected on different slices. There's no mechanism here for handling unpaired or weakly paired data—so what can SpaKnit do in those cases?

2. The paper claims robustness across datasets, but the model is trained separately on each dataset. There's no attempt at cross-tissue or cross-modality generalization. No few-shot or transfer learning experiment is conducted. So how would this work if I have only limited labeled multimodal data in a new tissue?

3. The manuscript never says how the data was partitioned into training, validation, or test sets. Was there a holdout? Was generalization assessed across slices? Without this, it's really hard to know whether the results reflect general patterns or overfitting.

4. Is L_{INR} optimized together with the joint Loss of stage 2? If not, how do you select the model eventually? How early stopping or best model checkpoints are chosen in stage 1,whether INR and DCCAE are cross-validated together or independently,whether downstream performance (e.g., ARI or Moran's I) is used for model selection.
The Human Lymph Node dataset is said to be "spatially allocated based on true labels" (p.16), but how exactly is this mapping done? Is there spatial autocorrelation in the raw data, or was it imposed by the simulation?

5. Gaussian and pepper noise are used to simulate technical variability (p.16), but are these really representative of spatial omics noise? Dropouts, autofluorescence, segmentation artifacts—those aren't discussed. Also, there's no detail on how noise levels were adjusted. Were there SNR thresholds?

6. Fig. 3a The reciprocal gradient method used here is unconventional. Though later Moran score makes more sense, the current choice feels a bit ad hoc.

7. Fig. 3c The dual-metric plot is great, but without error bars across slices, it's hard to tell how reproducible these metrics are.

8. Fig 4 The PAGA trajectories look compelling, but the parameters used for inference aren't reported. PAGA is sensitive to

clustering resolution and graph construction—how were these chosen?

9. Fig. 4a Claims about identifying cortex, medulla, and connective tissue aren't supported by marker gene expression or histological overlays. A simple validation would make these claims stronger.

10. The batch effects in Fig. 4d are shown visually, but no quantitative metric is used. Could the authors add something like batch entropy or LISI to objectively assess batch mixing?

11. The Gaussian kernel used in INR has σ fixed to 0.02—but why? Was this tuned? Did they test sensitivity? The spatial kernel is critical to performance and really should be better justified.

12. The benchmark mostly focuses on datasets with prominent tissue structures. What about rare cell types or smooth transitions between states?

(Remarks on code availability)

Reviewer #3

(Remarks to the Author)
I co-reviewed this manuscript with one of the reviewers who provided the listed reports. This is part of the Nature Communications initiative to facilitate training in peer review and to provide appropriate recognition for Early Career Researchers who co-review manuscripts.

(Remarks on code availability)
The code is well structured.

Version 1:

Reviewer comments:

Reviewer #1

(Remarks to the Author)
The author has addressed all my concerns.

(Remarks on code availability)
The code is well structured

Reviewer #2

(Remarks to the Author)
The revision has addressed major concerns.

(Remarks on code availability)

Reviewer #3

(Remarks to the Author)
I co-reviewed this manuscript with one of the reviewers who provided the listed reports. This is part of the Nature Communications initiative to facilitate training in peer review and to provide appropriate recognition for Early Career Researchers who co-review manuscripts.

(Remarks on code availability)

Open Access This Peer Review File is licensed under a Creative Commons Attribution 4.0 International License, which permits use, sharing, adaptation, distribution and reproduction in any medium or format, as long as you give appropriate credit to the original author(s) and the source, provide a link to the Creative Commons license, and indicate if changes were made.
In cases where reviewers are anonymous, credit should be given to 'Anonymous Referee' and the source.
The images or other third party material in this Peer Review File are included in the article's Creative Commons license, unless indicated otherwise in a credit line to the material. If material is not included in the article's Creative Commons license and your intended use is not permitted by statutory regulation or exceeds the permitted use, you will need to obtain permission directly from the copyright holder.
To view a copy of this license, visit https://creativecommons.org/licenses/by/4.0/

Reviewer #1

Li et al. proposed SpaKnit, a computational framework that models multi-omics data as continuous functions mapped to spatial coordinates, thereby facilitating the discovery of nonlinear correlations across modalities. By integrating spatial location with multi-omics information, SpaKnit aims to capture complex spatial patterns, enforce smoothness, and eliminate modality-specific noise. The authors demonstrated the effectiveness of their method on several simulated and public datasets, concluding that SpaKnit outperforms existing approaches. However, I have several major concerns regarding the conceptual novelty, validation strategy, and overall clarity of the manuscript.

Response: Thank you for your thoughtful review and constructive comments on our manuscript. We sincerely appreciate your positive recognition of our method's effectiveness in integrating spatial multi-omics data. Your comments regarding the conceptual foundation, performance evaluation, and manuscript clarity are highly valuable.

In response, we have revised the conceptual foundation, systematically supplemented the experiments, and further demonstrated the utility of our method in more challenging biological contexts.

We have thoroughly revised the manuscript to enhance its logical flow and overall clarity. The refined method is renamed **SpatialCOC**, standing for the first **cross-omics correction** method for spatial multi-omics data. We hope the revised version fully addresses your concerns and provides clearer articulation of our contributions.

1. Although the authors assert conceptual innovation, the overall methodological novelty of SpaKnit remains limited. The framework primarily comprises two modules: an Implicit Neural Representation (INR) module and a Deep Canonically Correlated Autoencoder (DCCAE) module. While the INR module has been slightly modified to suit the research goals, the DCCAE module is directly adopted from previous studies without significant innovation, which substantially weakens the manuscript's originality. Furthermore, the supposedly novel idea of modeling multi-omics data as continuous functions over spatial coordinates is used merely for modality reconstruction and, with independently trained SpaKnit components, contributes minimally during practical data analysis.

Response: Thank you for your valuable comment regarding the methodological novelty of our work. In response, we would like to clarify our design philosophy and provide additional evidence to highlight the originality and practical effectiveness of SpatialCOC.

Overall, compared with existing approaches that separately model spatial information and expression profiles and then concatenate them, SpatialCOC focuses on two core characteristics of spatial multi-omics data: spatial colocalization and complementarity among omics layers. By learning continuous omics functions from shared spatial coordinates, SpatialCOC builds a unified feature space that jointly embeds spatial and expression information, enabling it to model "corrected" cross-omics relationships. We have systematically designed experiments to validate its effectiveness, coordination, and expandability.

(1) Strategy Comparison: Bottom-up (Existing) vs. Top-down (SpatialCOC).

- Existing integrative methods employ a **"bottom-up"** strategy: these methods first model spatial information and various types of omics data separately, followed by a systematic, bottom-up fusion process.
- SpatialCOC adopts a **"top-down"** strategy: based on the spatial coordinates shared across multi-omics data, it learns joint representations of spatial

information and molecular expression in a top-down manner, thereby uncovering nonlinear and complementary relationships between different omics.

The “bottom-up” integrative approach provides a unified paradigm for spatial multi-omics integration, serving as a classic and effective framework for achieving multimodal unified representation. The core objective of SpatialCOC is to further leverage the two key characteristics of spatial multi-omics data: **spatial colocalization and complementarity among omics layers** (**Rebuttal Fig. 1**).

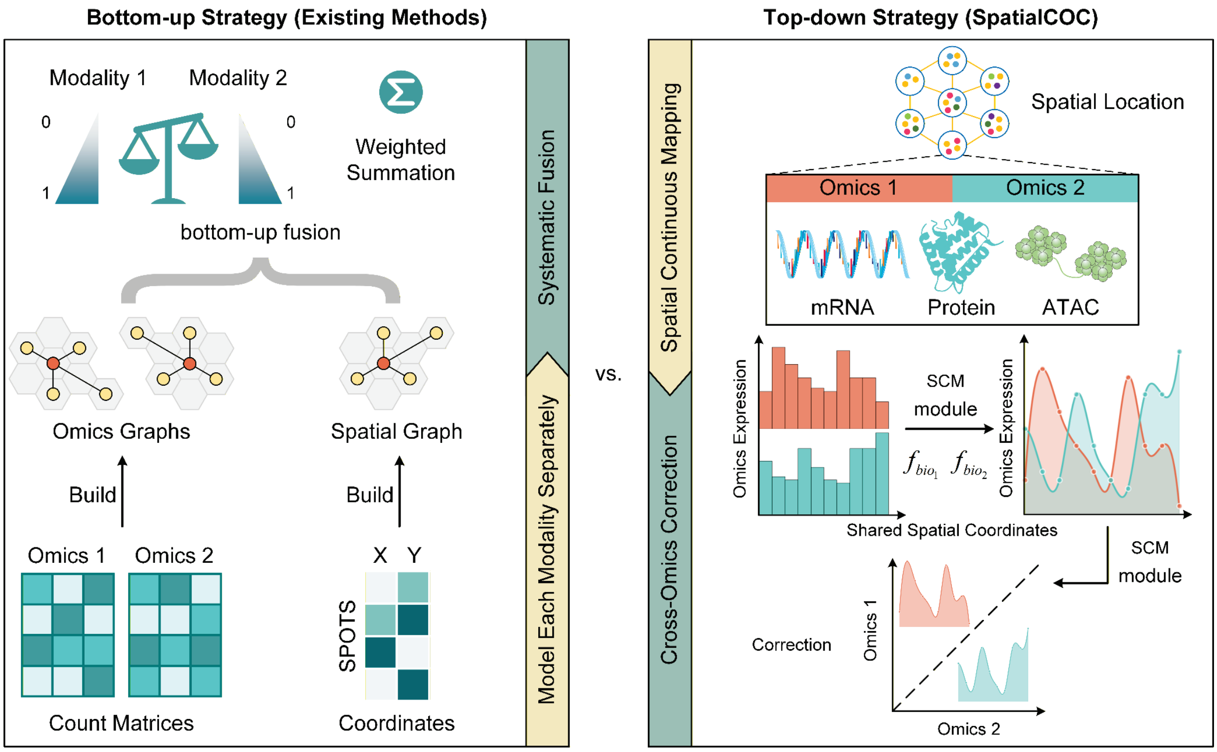

Rebuttal Fig. 1 | Comparison of Existing and Our Integration Strategies. Traditional integration strategies first project all modalities (omics and spatial coordinates) into a unified representation (e.g., neighborhood graphs) and then perform “weighted summation.” In contrast, our approach starts from spatial coordinates to learn a spatially continuous representation of each omics layer, followed by cross-omics correction.

(2) Strategy Implementation: The Spatial Continuous Mapping (SCM) module and the Cross-Omics Correction (COC) module.

- **SCM module**: Each omics modality was modeled as a shared-coordinate

continuous distribution reconstructed from observations. 1) we employ a **stepwise sinusoidal activation function** to fully accommodate distributional differences across omics data; and 2) we introduce **kernel functions** to enhance the local smoothness of representations. This design more effectively captures the heterogeneous spatial distribution across omics layers.

- **COC module**: This module enhances cross-omics integration through the following designs: 1) employing **a correction loss** function into the autoencoder to learn discriminative association signals among omics; 2) introducing **a linear canonical correlation analysis transformation** in the final layer, which more effectively achieves complementary alignment of features across diverse omics. These designs collectively contribute to obtaining complementary and nonlinear inter-omics relationships.

(3) Modular Training and Functional Coordination in SpatialCOC.

These two coupled modules—**rather than a simple concatenation**—to uncover global spatial patterns of omics and enable the joint discovery of nonlinear relationships **across both spatial and expression dimensions**.

- **Modular Training**: This allows the two modules of SpatialCOC to focus on their respective optimization objectives without interference. It also enables direct observation of each module's outputs to ensure effective and verifiable feature learning.
- **Functional Coordination**: The spatially continuous representations learned by the SCM module serve as input to the COC module. This design enables the corrections performed by the COC module to be conducted based on both "omics expression" and "spatial information" dimensions.

(4) More Evidences of Two Modules' Effectiveness

1. Ablation Experiments for Each Component

We conducted systematic ablation experiments on the two modules and the loss function used, which clearly demonstrated that each component was indispensable (**Rebuttal Fig. 2**).

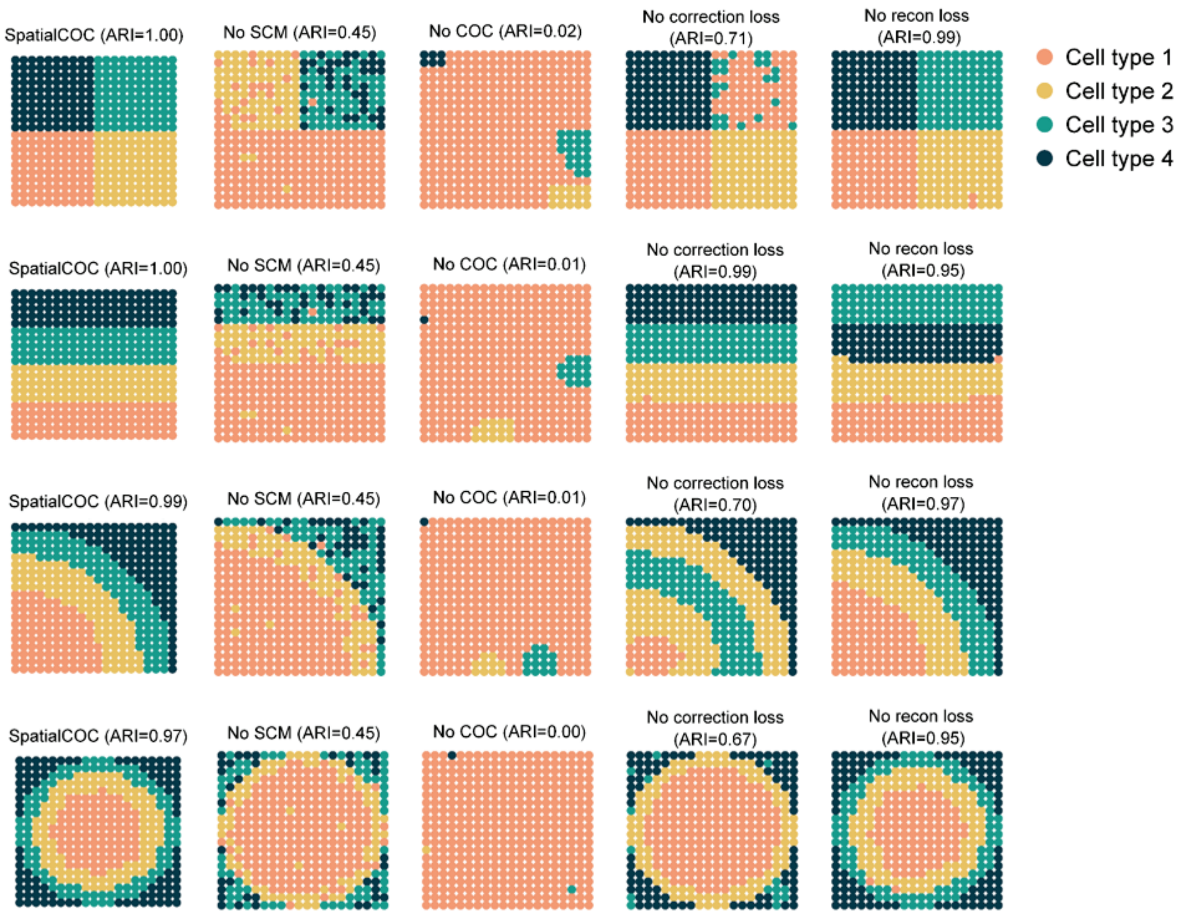

Rebuttal Fig. 2 | Systematic Ablation Experiments. Two modules were removed separately, as well as two loss functions used in the integrated training. The final spatial domain identification results were compared.

As shown in **Rebuttal Fig. 2**:

- Removing SCM module significantly reduced spatial coherence, while excluding COC module diminishes cross-modal integration.
- Removing the correction loss weakened inter-modality correlation, while eliminating the reconstruction loss disrupts the preservation of modality-specific features.

2. The Logical Coherence Between Modules

By capturing the globally continuous spatial distribution of omics data, the SCM module enables the COC module to better model nonlinear correlation across both expression and spatial contexts.

We conducted the following comparative experiment on the mouse brain dataset: changing the order of the two modules and comparing the resulting spatial domain assignments.

REDACTED

Rebuttal Fig. 3 | Inter-Module Logic Verification. The logical connection between the two modules was evaluated on the Mouse Brain dataset to demonstrate their logical interdependence. Using the annotation of P56 mouse brain coronal sections (Allen Mouse Brain Atlas[1], mouse.brain-map.org) as a reference.

As shown in **Rebuttal Fig. 3**:

- With current order (SCM followed by COC): Smooth and biologically meaningful results were obtained.
- In contrast, with the reverse order (COC followed by SCM), similar spatial smoothness was achieved, but a loss of biological significance was observed.

These experimental results demonstrate the close logical relationship and interdependence between modules. The SCM module facilitates the COC module in uncovering non-linear associations within both expression and spatial contexts.

(5) Expansion Potential of SpatialCOC

After conducting downstream analyses using multiple spatial multi-omics data, we added two extended scenarios: Cross-Slice and Few-Shot, to demonstrate the potential of SpatialCOC (**Rebuttal Fig. 4**):

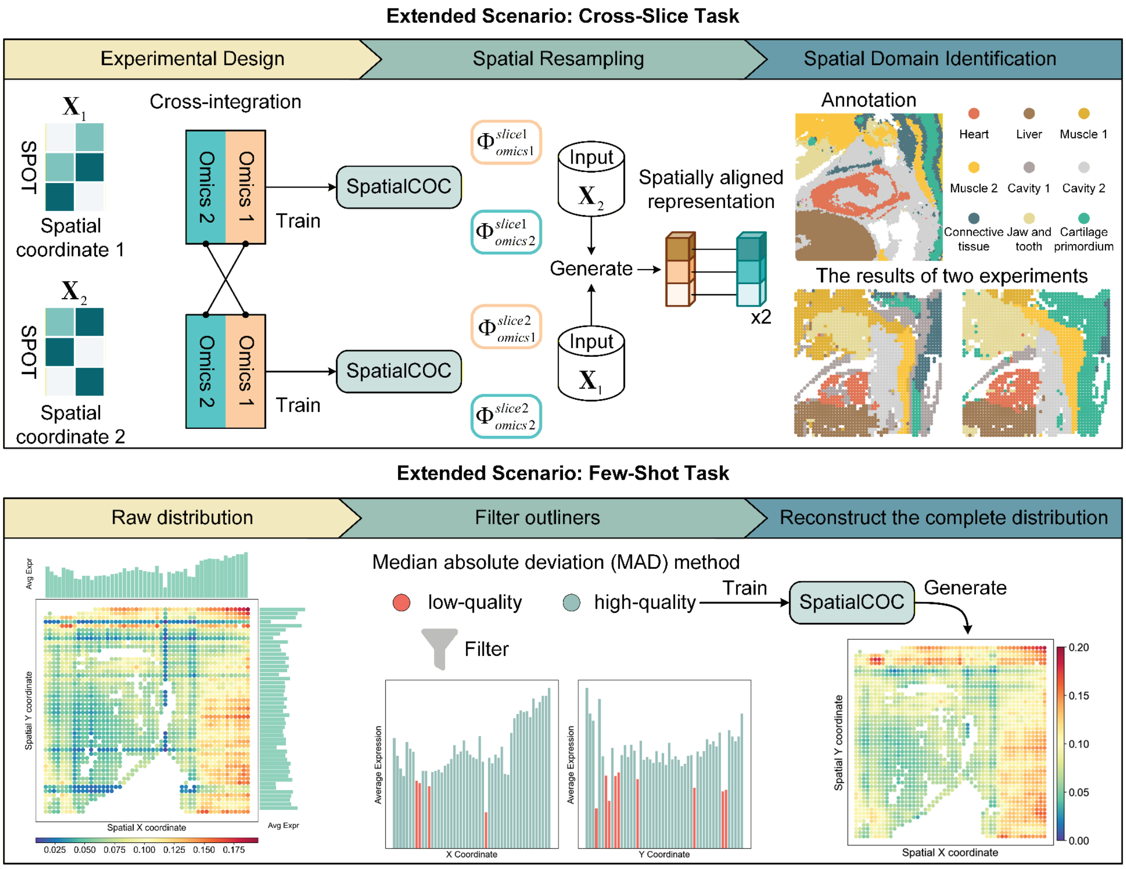

Rebuttal Fig. 4 | Extended Scenarios of SpatialCOC. Two extended scenarios were presented to illustrate the potential of SpatialCOC: Cross-Slice—where different omics data were collected from distinct tissue sections; and Few-Shot—where only a subset of high-quality sample points was available.

As shown in **Rebuttal Fig. 4**:

- In the Cross-Slice scenario, SpatialCOC leverages its inherent spatial continuity to integrate and model multi-omics data acquired across different tissue sections.
- In the Few-Shot scenario, SpatialCOC can generate a robust, globally continuous representation from only sparse, high-fidelity observations.

We emphasized the Spatial COC Expansion Potential in the discussion section of the main text.

In summary, SpatialCOC pioneers the concepts of spatially continuous representation and cross-omics correction in spatial multi-omics integration via two logically interconnected modules. Systematic ablation studies and logical verification between modules demonstrate the necessity and coherence of our design. Further

analysis of two extended scenarios underscores its potential. Collectively, these results highlight the conceptual contribution and practical effectiveness of SpatialCOC in spatial multi-omics integration tasks.

The relevant content is now stated in the following sections of the manuscript:

(1) SpatialCOC Framework Design

- Section “Results”, subsection “SpatialCOC Framework” (Lines 105-159).
- Section “Methods”, subsection “SpatialCOC Framework” (Lines 515-785).

(2) Evidences of Two Modules’ Effectiveness

- Extended Data Fig. 1b. Ablation Experiments (Line 954).
- Supplementary Note. 1, Subsection “The Logical Coherence Between Modules” (Lines 76-89).
- Supplementary Figure S3. Inter-Module Logic Verification (Line 510).

(3) Expansion Potential of SpatialCOC

- Section “Discussion” (Lines 393-400).

2. Despite some conceptual framework, the manuscript falls short in addressing key practical challenges, undermining the method's credibility in real-world scenarios. For instance, due to inherent differences in data formats among modalities, it is unclear how the proposed neural network is capable of reconstructing all modalities from spatial coordinates. Moreover, the framework reconstructs smoothed, rather than raw, data. The smoothing strategy, however, risks obscuring sharp transitions at spatial domain boundaries, potentially masking biologically meaningful expression changes.

Response: Thank you for your valuable comment regarding the real-world applicability of our method. We fully agree that an effective spatial integration strategy must explicitly model cross-modal heterogeneity while faithfully preserving biologically meaningful variation.

We have supplemented Module 1's effectiveness in capturing spatial information from three perspectives: foundational mathematics, experimental validations, and real-world scenario analyses.

(1) Foundational Mathematical Principles

- The Universal Approximation Theorem[2] ensures that an Implicit Neural Representation (INR) equipped with non-polynomial activation functions—such as the sinusoidal units used in Module 1—can approximate any continuous function. Therefore, the network can represent complex distributions with sharp gradient discontinuities precisely at domain boundaries.
- Complementing this theoretical guarantee, a Fourier perspective[3] reveals that the deep composition of sinusoidal layers generates a richly parameterized frequency spectrum. This spectrum can be tuned to match the high-frequency components present in raw multi-omics measurements, enabling the model to capture abrupt expression changes without imposing a priori smoothing.

Two parameters are critical for capturing the spatial patterns of the omics data:

- ω_0: Used in Equation (5) of the main text, it controls the frequency of the

sinusoidal activation function and determines the ability to capture global spatial information and keeping continuity;

- σ: Used in Equation (16) of the main text, it is the bandwidth parameter of the Gaussian kernel, which enhances local continuity.

(2) Experimental Validations of SCM Module's Capabilities

1. Validating ω_0 for Capturing Diverse Spatial Patterns

We have conducted additional experiments to further validate the method's ability to handle cross-modal heterogeneity and to accurately resolve spatial domain boundaries when using the sine activation function of appropriate frequency (**Rebuttal Fig. 5**).

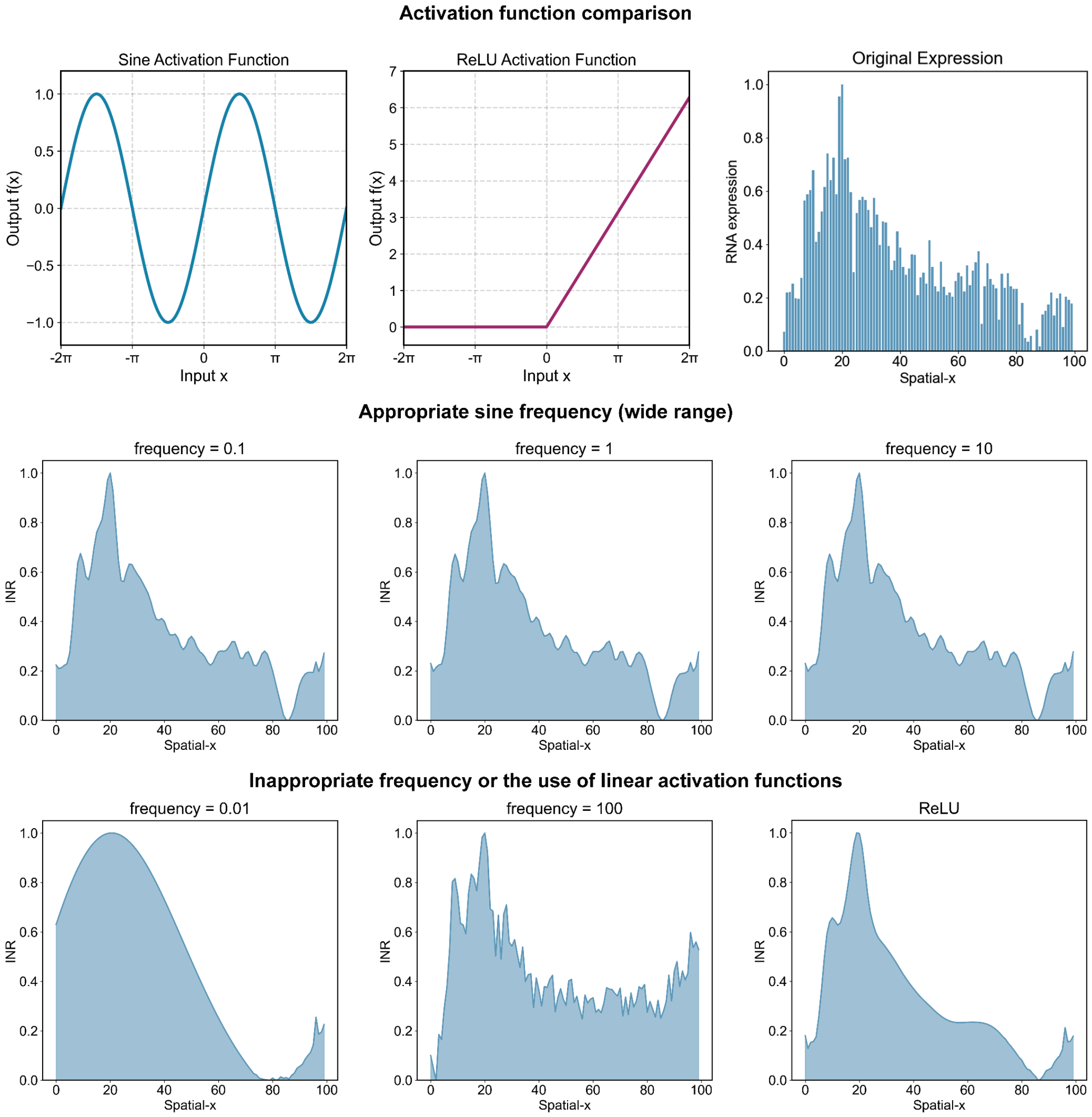

Rebuttal Fig. 5 | Comparison of Activation Functions. Performance of different activation functions in the SCM module was tested on the ATAC slice from the mouse

brain dataset. Sinusoidal functions at varying frequencies were assessed for data reconstruction and benchmarked against a linear activation function (ReLU).

As shown in **Rebuttal Fig. 5**:

- A sinusoidal activation function with an appropriate frequency reliably captures the continuous data distribution, including boundary signals. Moreover, the effective frequency range is broad, making parameter selection straightforward.
- Frequencies that are either too high or too low, or the use of a linear activation function, result in failure to capture boundary signals accurately.

2. Validating σ for Enhancing Local Smoothness

This parameter serves as a smoothness regularizer in SpatialCOC. A larger value enhances the spatial smoothing effect, while a smaller value reduces it. Accordingly, we have set its default to a relatively small value of 0.02, which provides only limited local smoothing. We have tested this setting across all datasets and confirmed its consistently strong performance.

We supplemented the comparative experiments to demonstrate the influence of Kernel (**Rebuttal Fig. 6**).

REDACTED

Rebuttal Fig. 6 | Ablation Study of the Kernel Function. The kernel function was ablated and the activation frequency was appropriately adjusted. Spatial domain identification was then performed on the mouse brain dataset for comparison. Using the annotation of P56 mouse brain coronal sections (Allen Mouse Brain Atlas[1],

mouse.brain-map.org) as a reference.

As shown in **Rebuttal Fig. 6**, After removing the kernel, the overall results are largely preserved, with only a reduction in local smoothness. Based on your comment, we have made it an optional parameter. Since there is an inherent trade-off between smoothness and boundary definition that cannot be perfectly balanced, users can now choose whether to use it based on their specific needs.

(3) Real-World Scenario Analyses

Real-world spatial multi-omics data typically exhibit the following characteristics: high dimensionality, multi-level biological meanings, and cross-scale mismatch. Our strategy is capable of adapting to omics modalities with significant differences across these aspects:

- **High Dimensionality.** While some omics data types, such as epigenomic and transcriptomic data, are high-dimensional, others like targeted proteomics are lower-dimensional. Our method demonstrates robust performance across this spectrum of data dimensionalities, including on their PCA-reduced embeddings (**Rebuttal Fig. 7**).

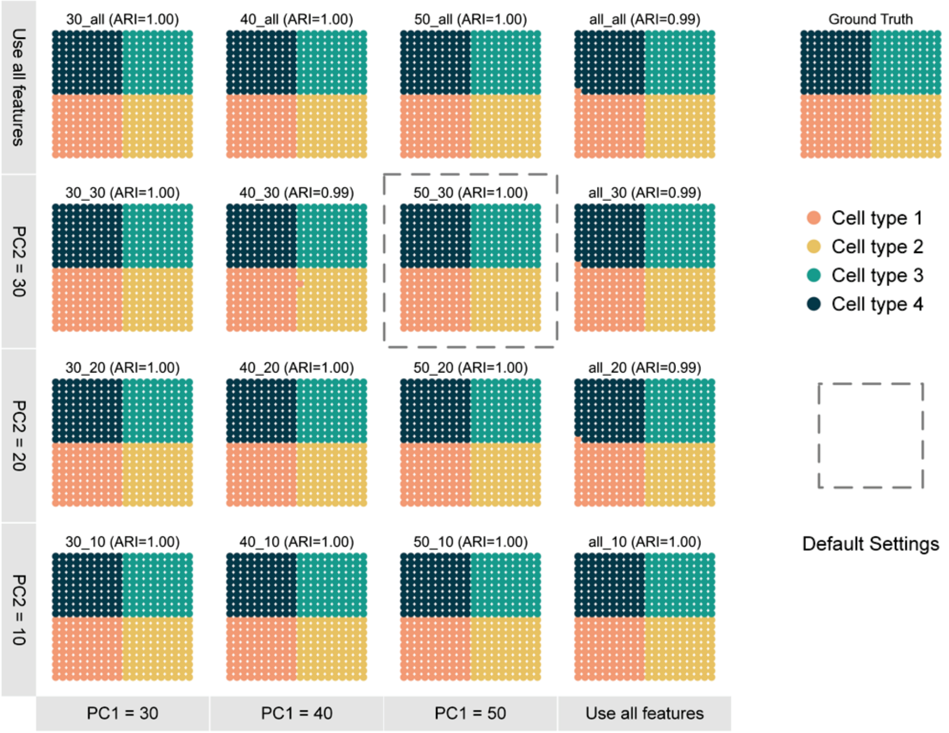

Rebuttal Fig. 7 | Sensitivity Analyses of Input Dimensionality. The performance was assessed using a range of principal components from PCA dimensionality

reduction, as well as the original data.

- **Multi-Level Biological Meanings.** We have expanded our real-world scenario analyses to include a diverse set of omics modalities, such as epigenomics (ATAC, H3K4me3, H3K27ac, H3K27me3), transcriptomics, and proteomics.
- **Cross-Scale Mismatch.** Multi-omics data may be measured on different tissue sections and some measurements may suffer from sparse feature representations. We conducted supplementary experiments that validate the method's robust alignment capability in addressing the cross-scale mismatch problem (**Rebuttal Fig. 4, in response to your Comment #1**).

We presented the characteristics of the datasets used, including the omics modalities, the number of features, and the sparsity (**Rebuttal Table 1**).

Rebuttal Table 1. Omics Characteristics of Experimental Datasets. A summary of the included omics as well as the average sparsity, feature dimensionality, and sparsity for all experimental datasets, demonstrating the diverse testing conditions for SpatialCOC.

Name	Omics and Sparsity	Feature Size
HLN-Augmented Datasets (Spatial Patterns of Varying Complexity)	transcriptomics: 89.17%, proteomics: 2.34%	400x3,000<br>400x31
HLN-Augmented Datasets (Different Noise Levels and Combinations)	transcriptomics: 89.17%, proteomics: 2.34%	1,200x3,000<br>1,200x31
Mouse Brain	transcriptomics: 93.81%, epigenomics: 97.18%	9,215x22,914 9,215x121,068<br>9,548x22,731 9,548x35,270<br>9,370x23,415 9,370x104,162<br>9,752x25,881 9,752x70,470
Mouse Spleen	transcriptomics: 89.50%, proteomics: 0.41%	2,568x32,285 2,568x21<br>2,768x32,285 2,768x21
Mouse Thymus	transcriptomics: 96.48%, proteomics: 0.50%	4,253x23,529 4,253x19<br>4,646x23,960 4,646x19<br>4,228x23,221 4,228x19

Overall, through solid mathematical theoretical foundations and comprehensive experimental validation, SpatialCOC has demonstrated the excellent capability of its proposed modules in capturing multi-omics features, including critical boundary signals.

Meanwhile, we conducted a systematic analysis of the real-world datasets used, covering a wide range of vastly different modality types—from high-dimensional epigenomics to low-dimensional proteomics, and from sparse to dense data—ensuring the broad applicability of the method. Collectively, these efforts substantiate SpatialCOC's effective and robust comprehensive capability in addressing key practical challenges.

The relevant content is now stated in the following sections of the manuscript:

(1) Foundational Mathematical Principles

- Supplementary Note.1, Subsection "Foundational Mathematical Principles" (Lines 24-36).

(1) Experimental Validations of SCM Module

- Supplementary Note.1, Subsection "Experimental Validations of ω_0" and "Experimental Validations of σ"(Lines 37-75).
- Supplementary Figure S1. Comparison of Different Activation Functions Used in SCM Module (Line 492).
- Supplementary Figure S2. Effect of Kernel Removal (Line 501).

(2) Real-World Scenario Analyses

- Extended Data Fig. 1b. Sensitivity Analyses of Input Dimensionality (Line 954).
- Supplementary Table S2. Description of Experimental Datasets (Line 585).

3. The evaluation of the method is overly simplistic. It relies primarily on simulated data and uses spatial smoothness as the sole quantitative metric. The manuscript does not offer sufficient evaluation using real, complex datasets or more comprehensive performance metrics. Importantly, the authors' justification that their method achieves a balance—even when smoothness scores are inferior to other approaches—fails to compensate for the lack of rigorous metric design.

Response: Thank you for your valuable comment regarding the evaluation rigor. In our revised manuscript, we have significantly strengthened the evaluation to address this point:

(1) Evaluation on Real, Complex Datasets.

A robust evaluation of integrative methods must address the inherent spatial structural heterogeneity and the authentic technical noise found in real tissues:

1. Diversity Spatial Distribution Patterns.

- **Smooth Gradients:** Cell types or gene expression can form smooth continuous gradients within tissues, for instance, the gradual changes in gene expression observed across the cerebral cortex[4].
- **Sharp Boundaries:** At the interfaces between tissue domains, abrupt transitions in cellular composition or gene expression often occur, forming sharp anatomical boundaries, such as the demarcation between the hippocampus and the cortex[1].
- **Hierarchical Structures:** Within tissues and organs, such as pancreatic germinal centers[5] or thymic lobules, the spatial arrangement of cells often exhibits clear hierarchical or zonal organization.
- **Complex Regions:** Examples include organ primordia during mouse embryonic development, such as the developing liver and heart in the embryo[6].

2. Authentic Noise Interference.

- **Measurement errors** introduced by limitations in spatial resolution.

- **Molecular integrity** due to fluctuations in molecular capture efficiency (e.g., gene dropout events[7]).
- **Batch effects**[8] that are unavoidable across different experimental batches or sample preparation procedures.

These complex spatial patterns and noise Interference present a significant challenge to the robustness and generalizability of SpatialCOC. Therefore, we conducted a systematic evaluation across multiple simulated scenarios and several real biological contexts (**Rebuttal Fig. 8**).

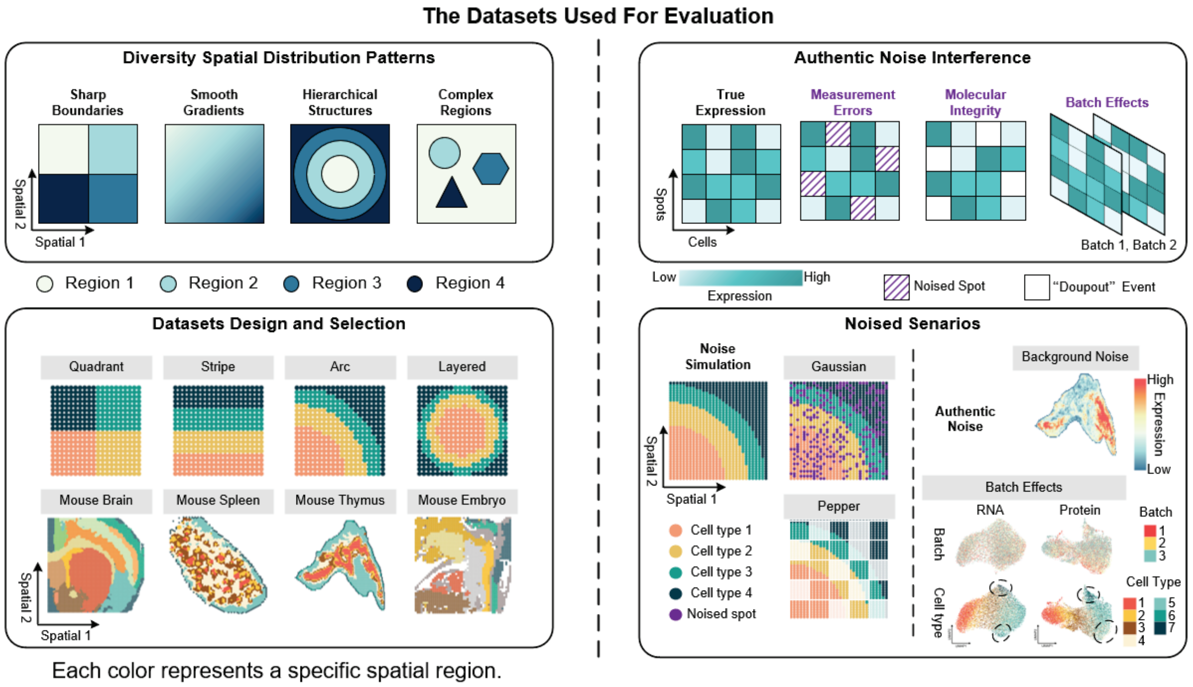

Rebuttal Fig. 8 | The Datasets Used For Evaluation. For both virtual data simulation and real-world data selection, we took into account the following diverse spatial distribution characteristics: smooth gradients, sharp boundaries, hierarchical structures, and complex regions. We also considered the following real-world noise factors: measurement errors, molecular integrity, and batch effects.

As shown in **Rebuttal Fig. 8**:

1. In the simulated dataset, the spatial structures of various real tissues have been simulated except for the complex regions. Moreover, the selected real datasets all cover the following types of spatial structures:

- Mouse Brain Dataset: Mixed Smooth Gradient and Clear Boundary Regions.

- Mouse Spleen Dataset: Central–Peripheral Pattern with Non-smooth and Smooth Regions.
- Mouse Thymus Dataset: Hierarchical Structure.
- Mouse Embryo Dataset: Complex Tissue Architecture.

In Supplementary Note. 7, we conducted a detailed analysis of the spatial characteristics of each dataset.

2. In our simulated data, we modeled measurement errors by introducing Gaussian noise, and represented molecular integrity degradation using pepper noise. Furthermore, we assessed the method's performance under real-world noise scenarios—including background noise and batch effects—by analyzing three slices from the mouse thymus dataset.

(2) Comprehensive Performance Metrics: We evaluated the effectiveness of our method from multiple perspectives, including clustering quantitative accuracy, spatial information capture, and downstream analysis accuracy.

- For Spatial Clustering: The Adjusted Rand Index (ARI), Adjusted Mutual Information (AMI), and Normalized Mutual Information (NMI).
- For Spatial Pattern Capture: The Moran's Index score and CHAOS score.
- For Trajectory Inference Accuracy: The evaluation system proposed by Wouter Saelens[9], augmented with a graph-based batch effect metric.

All datasets and the corresponding metrics applied are comprehensively summarized in the **Rebuttal Table 2**. This expanded evaluation robustly demonstrates our method's performance across various realistic and challenging scenarios.

Rebuttal Table 2. Evaluation Metrics and Corresponding Figures.

Name	Evaluation metrics	Related figures
HLN-Augmented Datasets (Spatial Patterns of Varying Complexity)	Adjusted Rand Index (ARI)<br>Adjusted Mutual Information (AMI)<br>Normalized Mutual Information (NMI)	Fig. 2a-c,<br>Extended Data Fig. 1a,b,<br>Extended Data Fig. 2 a-e,<br>Fig. S4
HLN-Augmented Datasets (Different Noise Levels and Combinations)	Adjusted Rand Index (ARI)<br>Adjusted Mutual Information (AMI)<br>Normalized Mutual Information (NMI)	Fig.2 d-f,<br>Extended Data Fig. 3a-c,<br>Fig. S5

Mouse Brain	Moran's I score	Fig. 3a-c,<br>Extended Data Fig. 4a-f, 5a-f
Mouse Spleen	Moran's I score<br>CHAOS score	Fig. 3d-f,<br>Extended Data Fig. 6a-d, 7a-d
Mouse Thymus	Adjusted Rand Index (ARI)<br>CHAOS score<br>a graph-based batch effect measurement method	Fig. 4a-f,<br>Extended Data Fig. 8a-d, 9a-c

Note: The graph-based batch effect measurement method will be elaborated in response to your **comment #5**.

In summary, SpatialCOC was evaluated on multiple real-world datasets (including human lymph node, mouse brain, spleen, etc.). A comprehensive set of quantitative metrics—covering clustering accuracy, spatial information capture, and downstream analysis accuracy—was employed to ensure the rigor and validity of our evaluation framework.

The relevant content is now stated in the following sections of the manuscript:

(1) Evaluation on Real, Complex Datasets

- "Methods" Section, Subsection "Data" (Lines 477-481).
- Supplementary Note. 7 The Datasets Used For Evaluation (Lines 435-489).
- Supplementary Figure S6. The Datasets Used For Evaluation (Line 561).
- Supplementary Table S2. Description of Experimental Datasets (Line 585).

(2) Comprehensive Performance Metrics

- Supplementary Note 4. Evaluation Metrics (Lines 141-221).

4. The manuscript exhibits a troubling degree of arbitrariness in its biological interpretations. The authors designate certain clusters as biologically meaningful without providing supporting evidence, such as marker gene expression or known anatomical structures. This undermines the credibility of any biological insight claimed.

Response: Thank you for this valuable comment regarding the biological interpretations. We agree that robust supporting evidence is crucial.

In the revised manuscript, we have strengthened our biological interpretations by providing new supporting evidence—including anatomical references, multimodal biomarkers, and histological images—to enhance the credibility of our findings.

(1) Human Lymph Node (HLN)-Augmented Dataset

- **Ground Truth Label:** We evaluated our method against authoritative omics data and labels[10](**Rebuttal Fig. 9**), enabling a systematic and objective performance assessment.

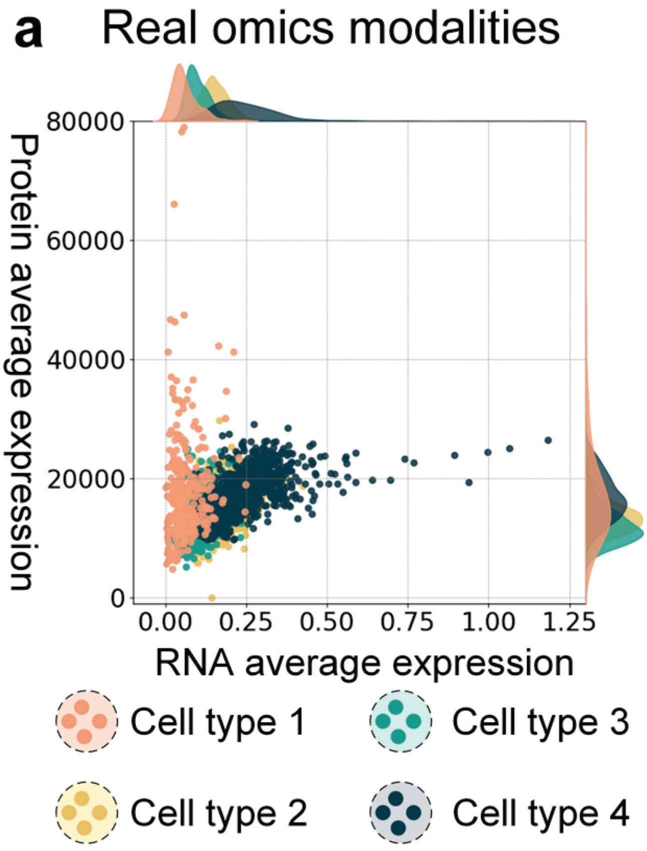

Rebuttal Fig. 9 | Real Omics Expressions and Labels from the Human Lymph Node Dataset.

(2) Mouse Brain Dataset

- **Known Anatomical Structures:** We supplemented a P56 mouse brain coronal section provided by Allen Mouse Brain Atlas[1] (Available from atlas.brain-map.org), and conducted detailed comparisons and visualizations between our spatial clustering results and this anatomical reference atlas (**Rebuttal Fig. 10**).

REDACTED

Rebuttal Fig. 10 | Comparison of the anatomical reference from the Allen Mouse Brain Atlas[1] coronal sections and the spatial domains identified by SpatialCOC on the ATAC slice of the mouse brain dataset.

- **Multimodal Biomarker Support:** We calculated and analyzed molecular markers in every tissue slice to further enhance the credibility of our results (**Rebuttal Fig. 11**). Visualizations are in Extended Data Figs. 4–5; detailed analyses are in Supplementary Note 6, “Supplementary Analyses of Multimodal Biomarkers”.

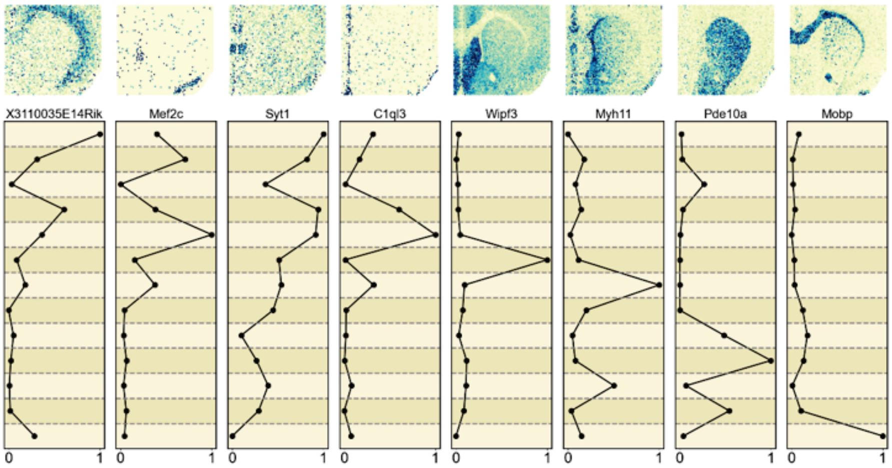

Rebuttal Fig. 11 | Marker Gene Identification: ATAC-seq Slice of Mouse Brain.

(3) Mouse Spleen Dataset

In the absence of ground truth annotations, we performed joint validation using H&E histological images and multimodal biomarkers:

- **Histological Validation**: Based on the histological image characteristics—divided into vesicular and smooth regions—we compared them with the clustering results (**Rebuttal Fig. 12**).

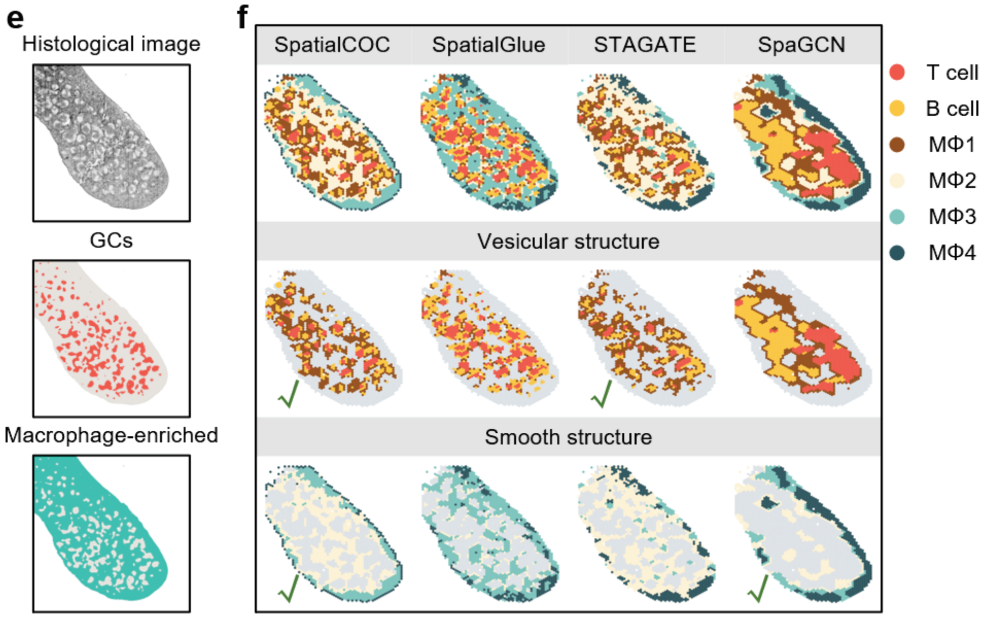

Rebuttal Fig. 12 | Histological Validation of Mouse Spleen Structures.

- **Multimodal Biomarker Support:** We have now supplemented analyses of marker genes and marker proteins for every cluster (**Rebuttal Fig. 13)**. Visualizations are in Extended Data Figs. 6–7; detailed analyses are in Supplementary Note 6, “Supplementary Analyses of Multimodal Biomarkers”.

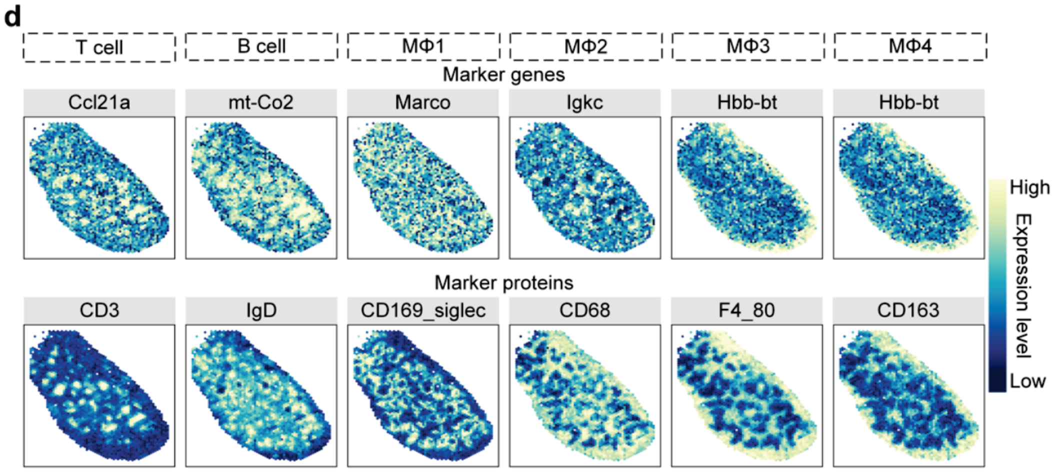

Rebuttal Fig. 13 | Multimodal Biomarkers of Mouse Spleen Dataset Slice 1.

(4) Mouse Thymus Dataset

No reliable labels or anatomical references were available for this dataset. We therefore assessed biological reliability through two cross-validation strategies: inter-section consistency, and multimodal biomarker support:

- **Inter-Section Consistency:** Parallel comparison of three replicate sections revealed that the major clusters as well as their trajectory are highly reproducible across replicates (**Rebuttal Fig. 14**).

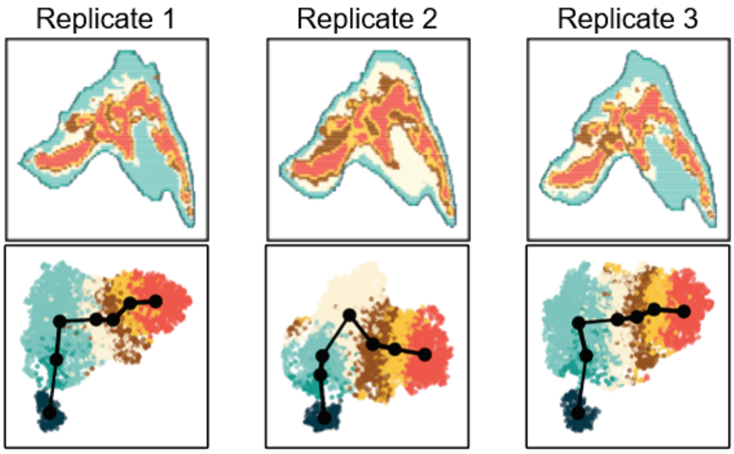

Rebuttal Fig. 14 | Inter-Section Consistency of Mouse Thymus Dataset.

- **Multimodal Biomarker Support:** Marker genes and proteins were calculated for each section (**Rebuttal Fig. 15**). Visualizations are in Extended Data Figs. 8; detailed analyses are in Supplementary Note 6, "Supplementary Analyses of Multimodal Biomarkers".

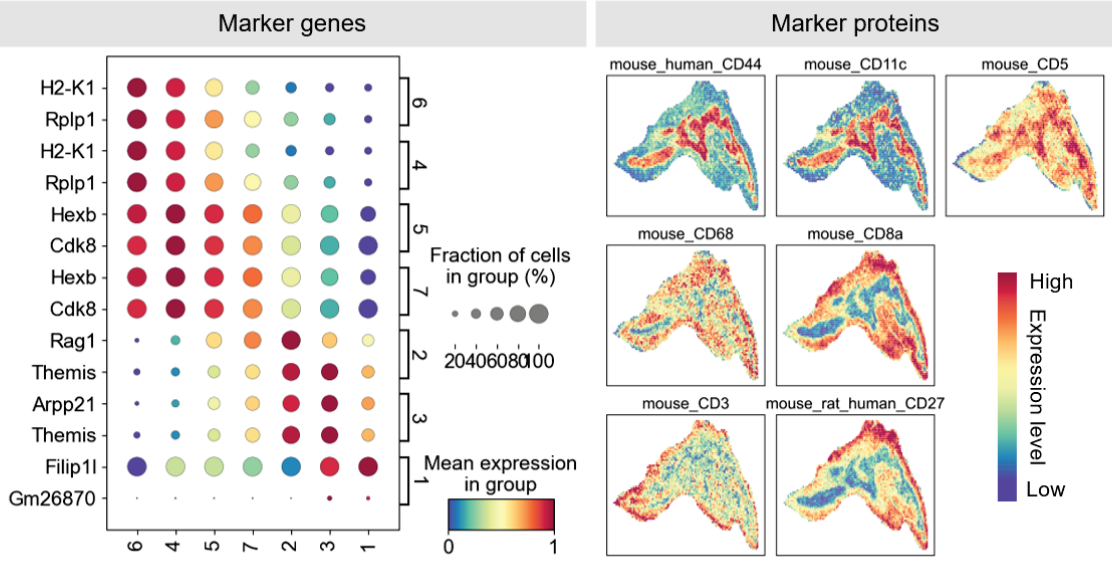

Rebuttal Fig. 15 | Multimodal Biomarkers of Mouse Thymus Dataset Replicate 1.

(5) **Mouse Embryo Dataset**

- **Known Anatomical Structures:** The spatial domain identification performance of SpatialCOC was assessed against the E13.5 mouse embryo annotation from the MOSTA[6] (Mouse Organogenesis Spatiotemporal Transcriptomic Atlas) as a reference (**Rebuttal Fig. 16**).

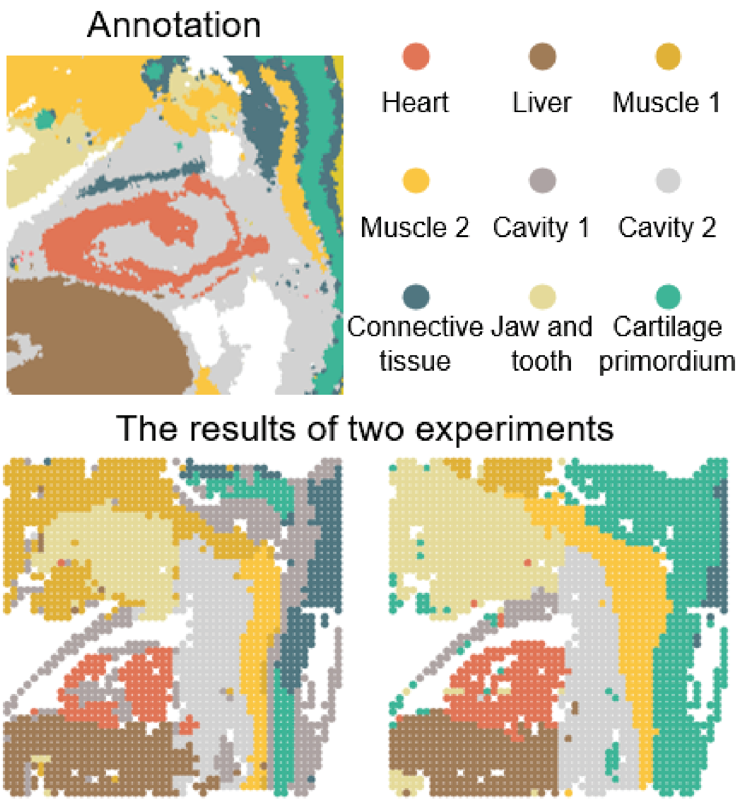

Rebuttal Fig. 16 | Comparison of the E13.5 mouse embryo annotation from the Mouse Organogenesis Spatiotemporal Transcriptomic Atlas (MOSTA) and the spatial domain clusters identified by SpatialCOC.

As this dataset is derived from epigenomic markers (H3K27ac, H3K27me3), the analysis of multimodal biomarkers was not performed. This case is presented to demonstrate the robust cross-slice integration potential of SpatialCOC.

In summary, the biological interpretations derived from all datasets were validated through multiple approaches, including referencing relevant anatomical structures, calculating multimodal biomarker support, and assessing consistency with histological images or across batches. These multifaceted validation strategies collectively enhance the credibility of our biological insights.

The relevant content is now stated in the following sections of the manuscript:

(1) Ground Truth Label or Known Anatomical Structures

- HLN-Augmented Dataset: Fig. 2a (Line 874).
- Mouse Brain Dataset: Extended Data Fig. 4a, d; 5a, d (Lines 1026, 1049).

(2) Multimodal Biomarker Visualizations and Analyses

- Mouse Brain Dataset: Extended Data Fig. 4c, f; 5c, f (Lines 1026, 1049).
- Mouse Spleen Dataset: Extended Data Fig. 6d; 7d (Lines 1073, 1092).
- Mouse Thymus Dataset: Extended Data Fig. 9 (Line 1125).
- A detailed description of all multimodal biomarker analyses is provided in Supplementary Note. 5 (Lines 223-356).

(3) Additional Supporting Evidences

- Histological Validation in Mouse Spleen Dataset: Fig. 3e (Line 898).
- Inter-section consistency in Mouse Thymus Dataset: Fig. 4e, f (Line 924).

5. Misalignment of methodological direction. The manuscript frames SpaKnit as a multi-omics integration tool, but it lacks comparison with state-of-the-art methods for multi-source data integration. In the context of multi-slice integration, no meaningful demonstration of batch effect removal is provided. The authors should incorporate benchmark comparisons in both domains.

Response: Thank you for your valuable comment regarding the benchmarking of multi-omics integration and batch effect removal. We have now included comparisons with state-of-the-art methods and introduced a graph-based metric to quantitatively assess batch effect removal.

(1) Spatial Multi-omics Integration Benchmarking: We have systematically included state-of-the-art methods that excel in both “effective use of spatial information” and “integration of multi-omics,” and have conducted comprehensive benchmarking centered on the following three key directions:

- Two spatial multi-omics data integration methods: SpatialGlue, and COSMOS.
- Three single-cell multi-omics data integration methods: Seurat WNN, MultiVI, and MultiMAP.
- Two spatial transcriptome methods: STAGATE, and SpaGCN.

All comparisons were conducted using a unified preprocessing pipeline. Each method was executed using either its default settings or a consistently chosen parameter set to ensure fairness. The full benchmark protocol, methodological details, and result analyses are given in the “Benchmarking methods” section of the Supplementary Information.

We believe that this systematic benchmarking enables a comprehensive and integrative assessment of our method's ability to integrate multi-omics data with spatial information.

(2) Demonstration of Batch Effect Removal: we have added quantitative metrics to better support our conclusions regarding the effectiveness of our method in removing batch effects.

Quantitative metrics for batch effect removal are primarily categorized into two types. The first relies on a shared embedding space to evaluate batch mixing, such as the Local Inverse Simpson's Index (LISI[8]), which quantifies label heterogeneity in local cell neighborhoods. The second assesses biological structural consistency without a shared embedding, as exemplified by SPIRAL[11], which employs Gromov-Wasserstein distance to measure the alignment of spatial or topological structures. We apply this second type of metric to evaluate the batch consistency of PAGA graph analysis results obtained from different methods.

Specifically, we quantified batch effects by calculating the cosine similarity between node-aligned PAGA graphs constructed from different biological replicates. This was achieved by searching through all possible node permutations to identify the best alignment between graphs from different replicates. A smaller distance indicates better reproducibility of biological structures across samples, reflecting more effective removal of batch effects.

We employed the Frobenius norm as our distance metric between adjacency matrices. For two graphs represented by adjacency matrices $\boldsymbol{A}$ and $\boldsymbol{B}$ of size $n \times n$, the graph distance under a permutation π is defined as:

$$D_{\pi}(\boldsymbol{A}, \boldsymbol{B}) = \| \boldsymbol{A} - \boldsymbol{P}_{\pi}^{T} \boldsymbol{B} \boldsymbol{P}_{\pi} \|_{F}$$

where:

- $\boldsymbol{P}_{\pi}$ is the permutation matrix corresponding to permutation π;
- $\| \cdot \|_{F}$ denotes the Frobenius norm.

Then we searched all possible node permutations to find the best alignment between graphs from different replicates:

$$D_{min}(\boldsymbol{A}, \boldsymbol{B}) = \min_{\pi \in S_n} D_{\pi}(\boldsymbol{A}, \boldsymbol{B})$$

where S_n is the symmetric group of all permutations on n elements. This optimal matching accounts for potential label switching between replicates.

Unfold the upper triangular parts of the aligned matrices $\boldsymbol{A}$ and $\boldsymbol{B}$ into two one-

dimensional vectors $\boldsymbol{a}$ and $\boldsymbol{b}$, and then calculate their cosine similarity: $similarity = \frac{\boldsymbol{a} \cdot \boldsymbol{b}}{\|\boldsymbol{a}\| \cdot \|\boldsymbol{b}\|}$ to quantify the batch effects.

Higher similarity scores in this comparison indicate better performance, as they reflect superior reproducibility and reduced batch effects across replicates (**Rebuttal Table 3**).

Rebuttal Table 3. Cosine Similarity Between PAGA Graphs

	SpatialCOC	SpatialGlue	Seurat	STAGATE
Rep1 vs Rep2	**0.967985**	0.936008	0.819309	0.963164
Rep1 vs Rep3	**0.992636**	0.874468	0.849186	0.943693
Rep2 vs Rep3	**0.96498**	0.84534	0.925135	0.919335

Rebuttal Table 3 presents a quantitative assessment of batch effect removal. The results show that our method consistently achieves higher cosine similarities (indicating better performance) in all pairwise comparisons. We have also incorporated a visualization of this metric (**Rebuttal Fig. 17**).

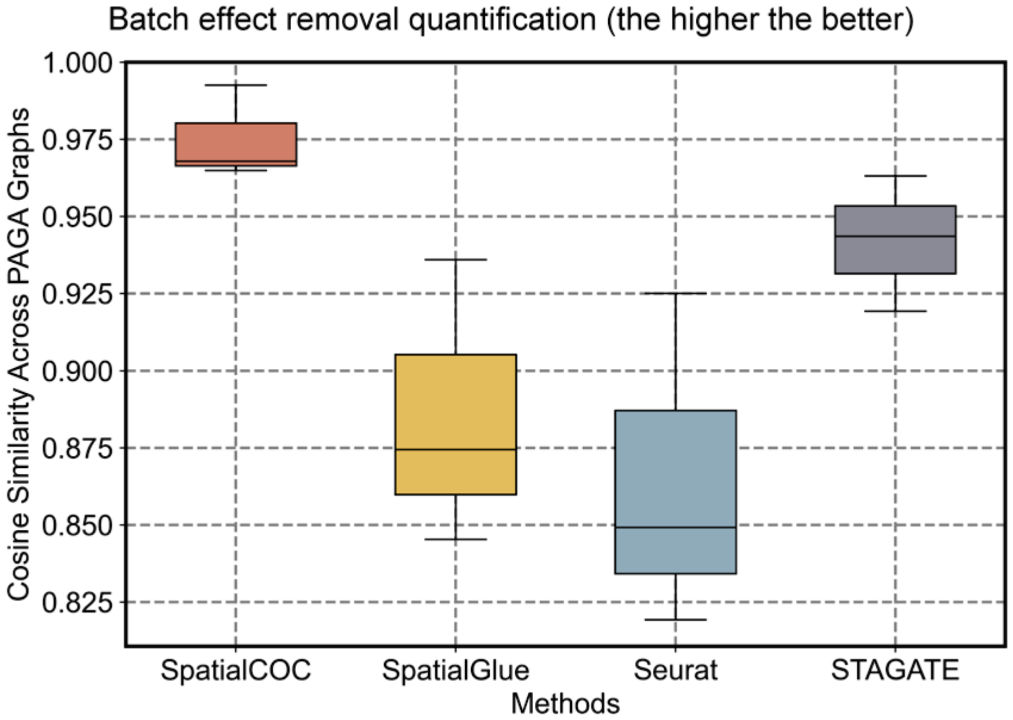

Rebuttal Fig. 17 | Batch Effects Removal Quantification (The Higher the Better).

In summary, by incorporating state-of-the-art methods for multi-source data integration, we established a comprehensive and systematic benchmarking framework.

Furthermore, we developed a graph similarity-based metric for batch effect evaluation, thereby enriching the quantitative evidence for batch effect removal.

The relevant content is now stated in the following sections of the manuscript:

(1) For Spatial Multi-Omics Integration Benchmarking:

- Section “Introduction” (Lines 96-100).
- Supplementary Note.3 Benchmarking Methods (Lines 99-139).

(2) For Demonstration of Batch Effect Removal:

- Fig. 4f Quantitative Measurement of Batch Effect Removal (Line 924).
- Supplementary Note.4 Evaluation Metrics (Lines 203-221).

6. Insufficient multi-Omics analysis. Given that SpaKnit is positioned as a multi-modal integration method, focusing solely on spatial domain identification is insufficient. The authors should provide detailed analyses across different modalities to substantiate the integrative capabilities of the method.

Response: Thank you for your valuable comment regarding multi-omics analyses. We sincerely appreciate the importance of demonstrating how a spatial multi-modal integration method should elucidate complementary relationships between different modalities beyond spatial domain identification.

We would like to clarify that this objective aligns directly with our core methodological principle. Our original manuscript already contained analyses demonstrating how the "cross-omics correction" strategy provides more reliable biological insights compared to conventional "weighted-sum" approaches. Building upon your valuable suggestion, we have now further strengthened and systematized these analyses in the revised manuscript, with the key enhancements detailed below:

(1) Addressing Modality-Specific Noise: We verified that our method effectively addresses inherent challenges in each modality. For instance, in response to pervasive background noise in transcriptomic data, our method performs corrective adjustments to generate a denoised and highly reliable joint embedding (**Rebuttal Fig. 18**). This embedding can be directly applied to downstream tasks beyond spatial domain identification—such as trajectory inference (demonstrated in subsequent analyses)—highlighting its generalizability.

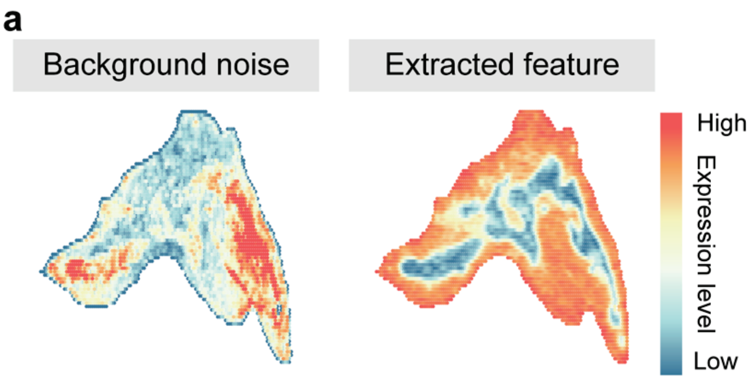

Rebuttal Fig. 18 | “Background Noise” and Feature Extraction in RNA Modality.

(2) Cross-Modality Denoising and Correction: To directly compare the advantages of the "cross-omics correction" strategy over the "weighted-sum" approach, we conducted graded noise perturbation experiments on three biological replicate sections of the Mouse Thymus Dataset (**Rebuttal Fig. 19**). The results indicate that, even under strong noise interference, biological patterns inferred using our method—such as cellular hierarchical structures—exhibit superior robustness compared to those identified by other methods.

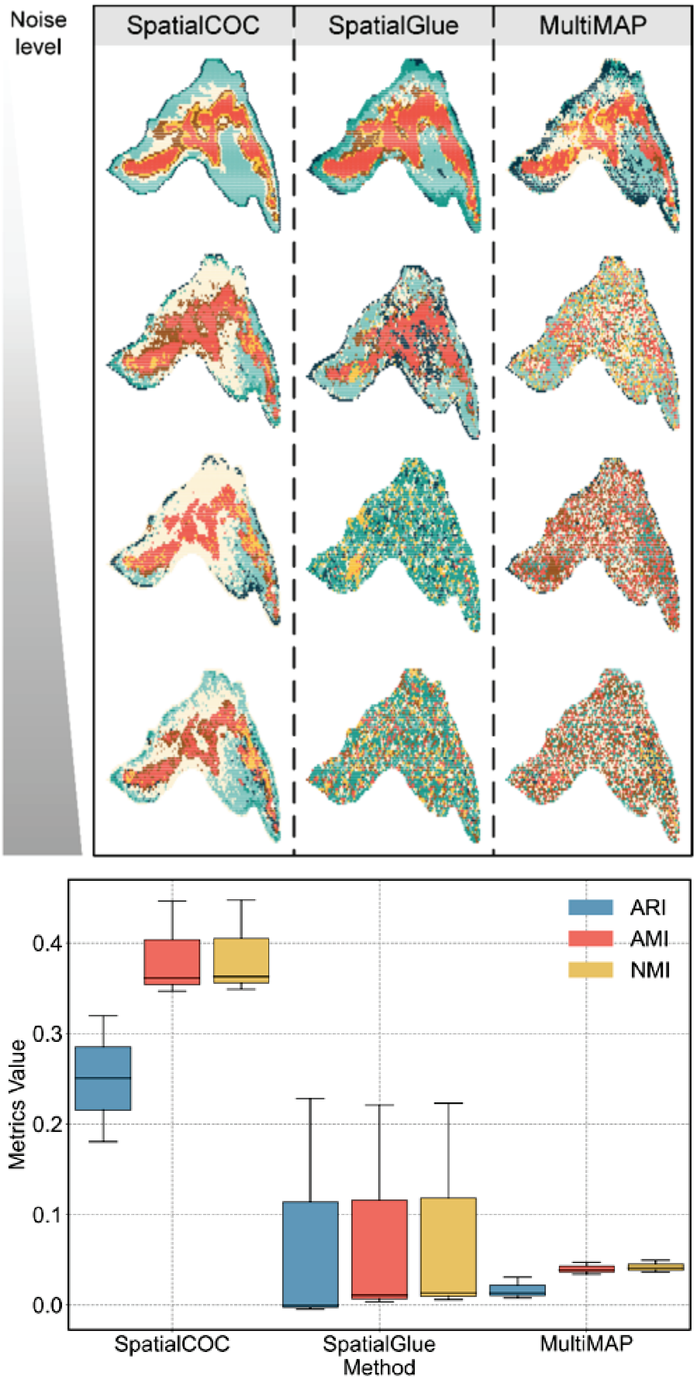

Rebuttal Fig. 19 | Robustness Test on Mouse Thymus Dataset.

(3) Removing Batch Effects and Enhancing Reproducibility: We further evaluated the stability of our integration method in cross-sample analyses (**Rebuttal Fig. 20**). By analyzing the consistency of trajectory inference results across three biological replicate sections, we demonstrated that our method significantly alleviated batch effects and ensured highly reproducible biological inferences across different experimental samples.

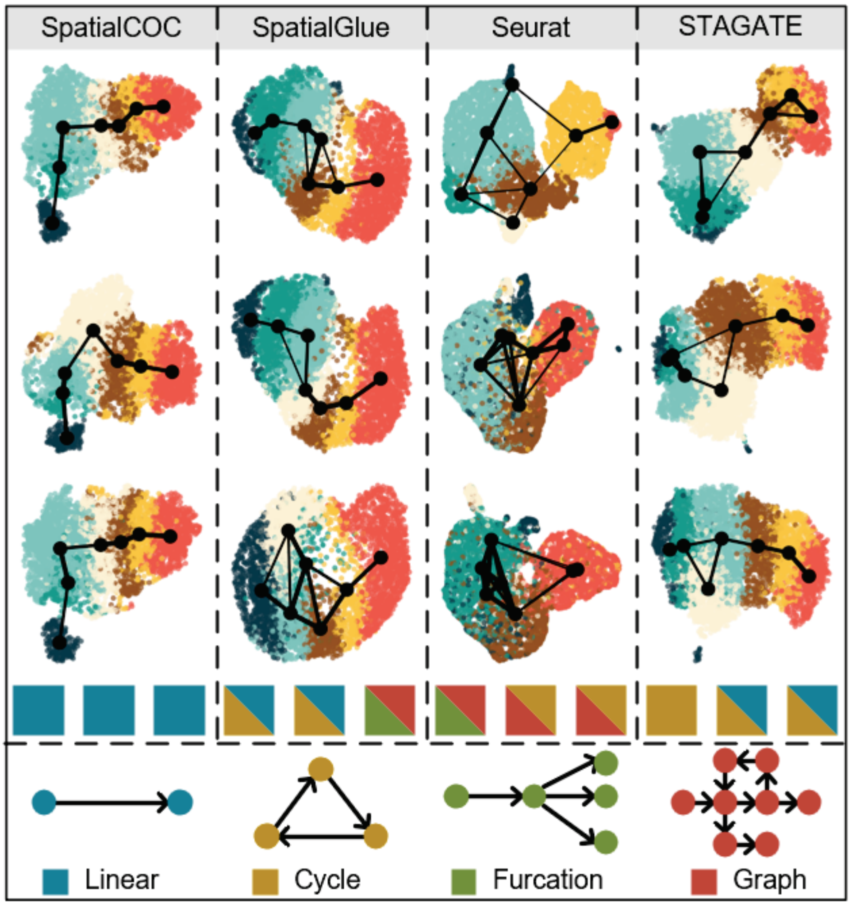

Rebuttal Fig. 20 | Trajectory Inference Performance.

In conclusion, these multi-omics analyses that go beyond spatial clustering and are in-depth, have made significant contributions in interpreting modal relationships, correcting data noise, and ensuring the reproducibility of results.

The relevant content is now stated in the following sections of the manuscript:

- Analyses: subsection "Achieving Robust, Batch-Consistent Trajectory Inference on Mouse Thymus Dataset" (Line 318-378).
- Visualizations: Fig. 4 Robustness Test and Trajectory Inference on Mouse Thymus Slices (Line 924).

7. The case studies presented involve only two datasets, which is insufficient to support the method's general applicability. Additional spatial omics datasets with biologically relevant contexts should be incorporated to demonstrate the utility and versatility of SpaKnit.

Response: Thank you for your valuable comment regarding the case studies. We appreciate this clarification. Our original presentation may have been unclear. To clarify, after introducing the integrative framework, we first validated its feasibility in **two foundational cases**, followed by a comprehensive analysis across **three real-world biological cases**, and culminating with **two extended cases** to demonstrate its broader potential.

(1) Two Foundational Case Studies

These studies were designed to systematically validate the effectiveness of the proposed framework:

- Diverse Spatial Distribution Patterns: Validates the effectiveness of the SCM module to capture varying spatial patterns (**Related to Fig. 2a-c; Extended Data Fig. 2; Supplementary Figure S4**).
- Different Noise Combinations: Demonstrates that the COC module can effectively extract and integrate multi-omics modalities under noisy conditions (**Related to Fig. 2d-f, Extended Data Fig. 3; Supplementary Figure S5**).

(2) Three Real-World Biological Case Studies

Each case highlights superior insights enabled by our framework, and the following are some of the key conclusions:

- Four slices of the Mouse Brain Dataset: Preserved a balance between spatial smoothness and biological relevance, accurately identifying mouse brain structures (**Related to Fig. 3a-c; Extended Data Fig. 4-5**).
- Two slices of the Mouse Spleen Dataset: Our method successfully captures region-specific spatial domain when analyzing regions with varying degrees of smoothness (**Related to Fig. 3d-f; Extended Data Fig. 6-7**)

- Three slices of the Mouse Thymus Dataset: Consistently inferred trajectories across independently analyzed slices, demonstrating robust reproducibility (**Related to Fig. 4; Extended Data Fig. 8-9**).

(3) Two Extended Case Studies

We have included two extended scenarios to demonstrate the potential of SpatialCOC:

- Cross-Slice: Joint modeling of multiple tissue sections.
- Few-Shot: Limited availability of high-quality data.

Detailed analyses of these extended case studies are provided in our response to **Comment #1, #2 of Reviewer #2**.

In summary, the employed datasets encompass a wide range of biologically relevant contexts, including human lymph nodes, as well as mouse brain, thymus, spleen, and embryo. Through our well-designed and rigorous case studies, we comprehensively demonstrate the utility and versatility of SpatialCOC.

Thank you for raising this important point and we hope this clarification addresses your concern regarding the rigor of the case study design.

Minor issues:

1. In Figure 1, the reconstruction error of the autoencoder should be computed on data with consistent dimensions. The discrepancy between the figure and the description in the Methods section is confusing.

Response: Thank you for your valuable comment regarding the methodological detail. We sincerely appreciate this observation and clarify that in the COC module, the reconstruction loss is computed between the reconstructed and original embeddings in the latent space, which share the same dimensions.

The original presentation in Fig. 1, intended to emphasize the denoising effect, may not have clearly illustrated this point, leading to potential confusion. Following your suggestion, we have revised Fig. 1 to accurately reflect the computational process and ensure consistency with the description in the Methods section, thereby improving the rigor and clarity of the manuscript.

The following is the detailed list of revisions:

(1) We use standardized squares with slashed filling to represent the embeddings before and after reconstruction, demonstrating dimensional consistency (**Rebuttal Fig. 21**).

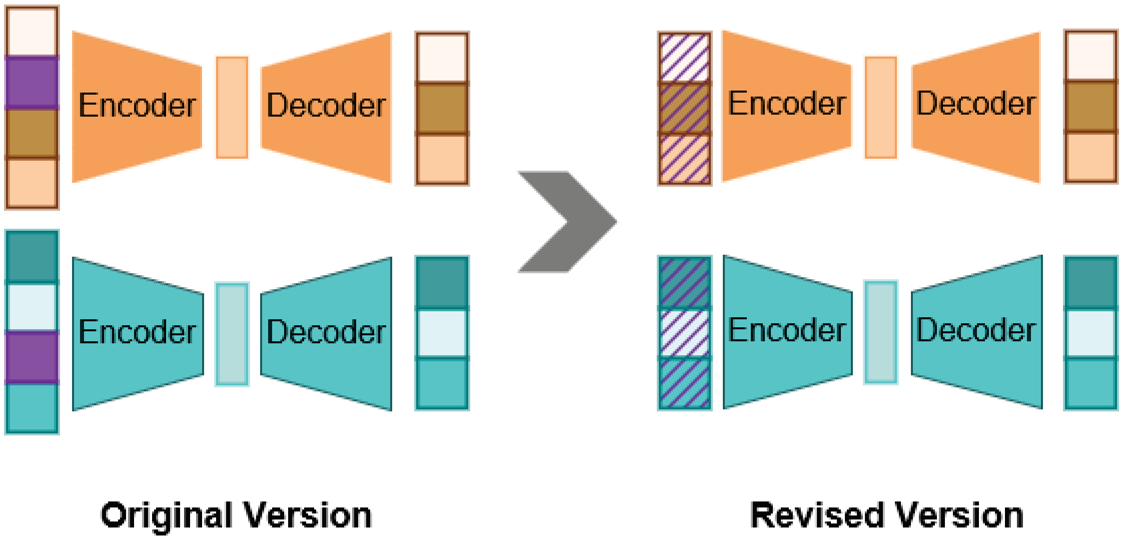

Rebuttal Fig. 21 | Embeddings Before and After Revision.

(2) **Rebuttal Table 3** lists the complete revised text for Fig. 1 in the main text:

Rebuttal Table 3. Original and Revised Text with Reasons for Revision.

Original Text	Revised Text	Reason for Revision
Single modality representation	Spatial information Integration	Emphasizing logic and alignment with the main text: First, spatial information is integrated through Spatial Continuous Mapping, followed by multi-omics integration via cross-omics correction between modalities.
Multimodal Integration	Multi-Omics Integration	
Implicit Neural Representation (INR) module	Spatial Continuous Mapping (SCM) module	
Deep Canonically Correlated Auto-Encoder (DCCAE) module	Cross-Omics Correction (COC) module	

Finally, we have provided the current version of Fig. 1 (**Rebuttal Fig. 22**) for your review. We hope the revised figure meets both rigorous and aesthetic requirements, and we thank you again for your valuable comment.

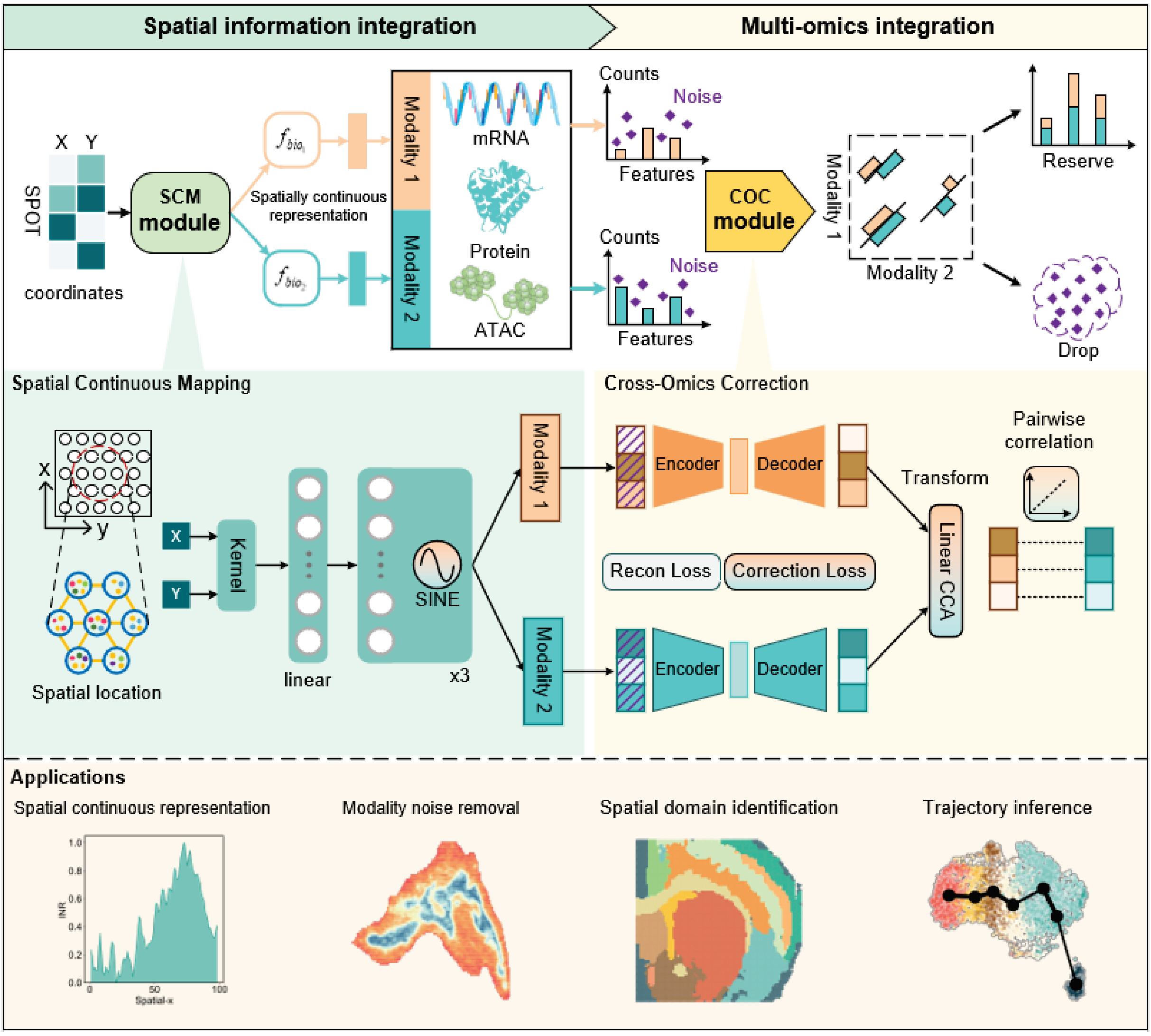

Rebuttal Fig. 22 | Overview of Spatial Multi-omics Integration using SpatialCOC.

2. There is inconsistent notation throughout the Methods section—for example, the symbol x is used ambiguously to represent different concepts.

Response: Thank you for your comment regarding the details of the Methods section. We have thoroughly revised this section to ensure notational consistency.

For clarity, we **define the key notations** used in this study. We consider a typical spatial multi-omics dataset with two omics layers, formalized as a tuple $\mathcal{D} = (\mathbf{X}, \mathbf{Z}_1, \mathbf{Z}_2)$. The tuple consists of spatial coordinates and two multi-omics matrices, defined as follows:

- **Spatial coordinates** $\mathbf{X} \in \mathbb{R}^{n \times 2}$ define the 2D locations of spots and are shared by each omics modality, where n denotes the number of spots, and $\mathbf{X}_i$ are the $i-\text{th}$ spot's spatial coordinates in $\mathbf{X}$.
- **Omics expression matrices** $\mathbf{Z}_1 \in \mathbb{R}^{n \times m_1}, \mathbf{Z}_2 \in \mathbb{R}^{n \times m_2}$ capture molecular profiles across two omics modalities, where m_1, m_2 are the respective feature dimensions of the two omics modalities.

Neural networks are employed in both stages of our framework. To avoid confusion, we define the following notations:

- **Embeddings:** $\mathbf{e}_{i1}^{(\cdot)}, \mathbf{e}_{i2}^{(\cdot)}$ denote the embeddings of the two omics in the SCM stage, while $\mathbf{h}_{i1}^{(\cdot)}, \mathbf{h}_{i2}^{(\cdot)}$ represent the corresponding embeddings in the COC stage.
- **Weights and Biases.** $\{\mathbf{W}_{\cdot\cdot}, \mathbf{b}_{\cdot\cdot}\}$ denote the trainable weight matrices and bias vectors in the SCM stage, while $\{\mathbf{U}_{\cdot\cdot}, \mathbf{c}_{\cdot\cdot}\}$ denote those in the COC stage.

Based on the definitions above, we have revised Equations (4–10) and (16), including their mathematical expressions and corresponding symbolic explanations. The relevant content is now stated in section “Methods”, subsection “SpatialCOC

Framework" (Lines 532-649, 725).

3. Appendix Figure 4c lacks an adequate explanation of the displayed gene expression patterns.

Response: Thank you for your comment regarding the marker gene analyses. We agree that a comprehensive interpretation of marker genes significantly enhances the credibility and biological relevance of our findings.

In response, we have made the following additions:

- Added descriptions of the marker genes in the main text. (Lines 267-273).
- We have added a new section in the Supplementary Information, which provides a detailed analysis of the Multimodal biomarker for each dataset (Supplementary Note 5, "Supplementary Analyses of Multimodal Biomarkers", Lines 223-356).

4. The Methods section should be expanded to describe downstream analyses, including spatial domain identification and trajectory inference strategies.

Response: Thank you for your valuable comment regarding the downstream methodology. In response, we have now moved the detailed descriptions of spatial clustering, differential gene analysis, and trajectory inference from the Supplementary Information to the Methods section of the main text to ensure better coherence and accessibility. Additionally, we have included specifics regarding UMAP-based dimensionality reduction visualization to provide a more comprehensive description of the methodology. We believe these revisions significantly improve the clarity and completeness of our methodological presentation.

The relevant content is now stated in the following sections of the manuscript:

(1) “Downstream Analysis” subsection of the Methods (Lines 786-816).

5. The English writing should be polished to improve readability.

Response: Thank you for your valuable comment regarding the readability of our manuscript. In response, we have conducted a comprehensive language revision of the entire manuscript. The following are some representative examples:

(1) Abstract

Original: …, Here, we introduce SpaKnit, a framework that treats multi-omics data as continuous functions mapped to spatial coordinates, …

Revised: …, Here, we introduce SpatialCOC, a framework that treats spatial information as prior knowledge to learn omics-specific spatial distributions, …

Reason: Clarify the key information "prior knowledge" and its purpose.

(2) Introduction

Original: Existing methodologies for integrating spatial multi-omics often rely on structured representations, such as neighborhood graphs that unify omics modalities and spatial information (e.g., SpatialGlue, SpaTrio, STAGATE, SpaGCN, and GraphST) to integrate multi-omics modalities by "weighted summation" or by identifying common components (e.g., Seurat WNN, MultiVI, MultiMAP, and GLUE).

Revised: Existing integrative methods for spatial multi-omics data often rely on structured representations—such as neighborhood graphs—to uniformly characterize multiple modalities (e.g., SpatialGlue, COSMOS, SpaTrio, STAGATE, SpaGCN, and GraphST). These methods subsequently integrate them by "weighted summation" or by extracting shared latent embeddings (e.g., Seurat WNN, MultiVI, MultiMAP, and GLUE).

Reason: Split it into two sentences for easier reading.

(3) Results

Original: To address the unique challenges of spatial multi-omics and fully utilize the potential of omics modalities and spatial information, …

**Revised:** To address the key challenges of integrating spatial multi-omics—the diversity in the spatial distribution of omics data and the nonlinearity of inter-omics relationships
**Reason:** Add key information.

(4) Discussion

**Original:** An exciting future direction is extending the INR module to incorporate multi-scale data, such as image data, tissue microenvironment data, and temporal dynamics.
**Revised:** Extending the SCM module to handle non-aligned data will be a key focus for future development, which will require systematic alignment strategies.
**Reason:** Remove redundant information.

We have revised the entire text to enhance its readability. For any revised content, we have marked it with the #006D77 color and supplemented it with italicized underlines. Please refer to the revised manuscript.

Reviewer #1 (Remarks on code availability):

The code provides an explanatory document that elaborately introduces the presentation of each figure in the article

Response: Thank you for your thorough review on our codes. We believe that a comprehensive open-source multi-omics integration platform can significantly contribute to the advancement of this field. For any questions, please feel free to contact us on GitHub.

Reviewer #2

Summary

SpaKnit proposes a novel framework for spatial multi-omics integration by modeling modalities as continuous spatial functions and aligning them via a deep canonical correlation autoencoder. It clearly addresses some known limitations of prior methods like reliance on local graphs or linear summation. However, it is not convincing that SpaKnit really solves the core integration challenges in a way that's broadly useful or generalizable. For example, what happens when we don't have perfectly paired multi-modal data? Can it do anything in low-sample or few-shot settings? These are real bottlenecks in spatial omics today. Also, the lack of a clear training/validation/test split makes it hard to assess generalization at all.

Strengths

1. SpaKnit treats each modality as a continuous function of space via Implicit Neural Representation (INR), enabling spatially smooth, resolution-independent reconstruction.

2. DCCAE models nonlinear correlations between modalities while explicitly removing modality-specific noise.

3. The benchmarking across multiple synthetic spatial patterns and real datasets is thorough and generally supports the method's robustness, especially under noise perturbation.

Response: Thank you for acknowledging the innovation and systematic benchmarking in our manuscript, as well as for raising profound insights into the universality of the methods. These comments are crucial for improving our work.

To clarify, our method has been renamed **SpatialCOC**, standing for the first **cross-omics correction** method for spatial multi-omics data.

Below, we provide detailed responses to each of the specific concerns raised.

1. SpaKnit is trained on paired modalities with shared spatial coordinates. But in reality, spatial transcriptomics and proteomics (e.g., Slide-seq and CODEX) are often collected on different slices. There’s no mechanism here for handling unpaired or weakly paired data—so what can SpaKnit do in those cases?

Response: Thank you for your valuable comment regarding the applicability of our method. We first clarify the primary design objective and then present experimental evidence demonstrating the framework's extension to cross-slice integration tasks.

Previous work[12] has categorized multi-omics integration algorithms into three aspects (**Rebuttal Fig. 23**):

- Horizontal integration: Focuses on deciphering the associations across different omics modalities;
- Vertical integration: Primarily addresses batch effect correction;
- Mosaic integration: Integrates datasets that share at least one type of omics modality data.

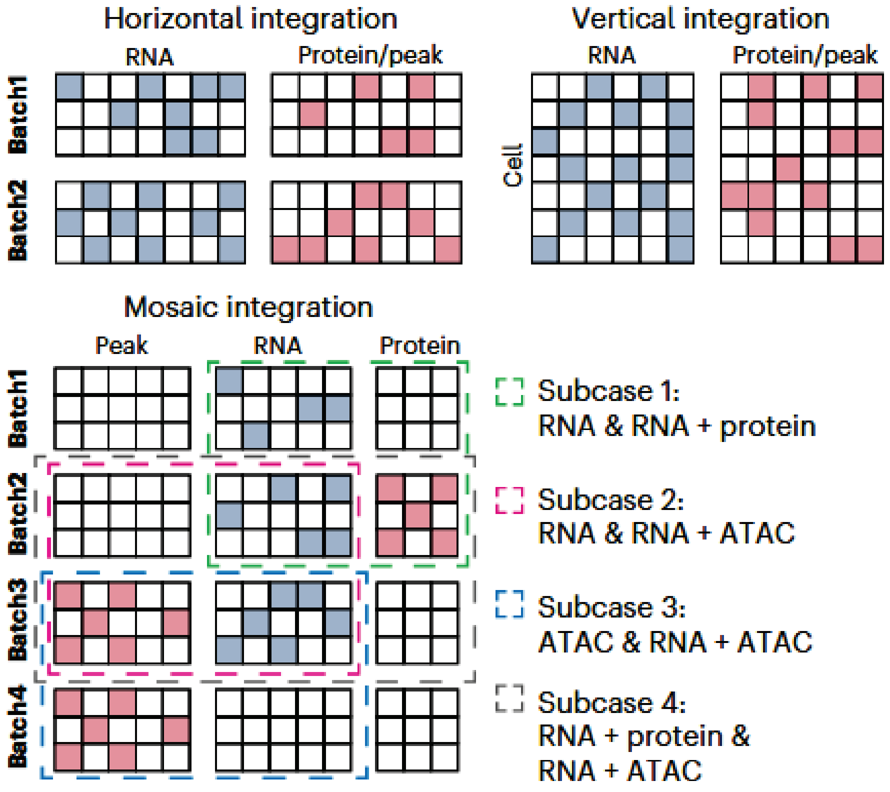

Rebuttal Fig. 23 | Multi-Omics Integration Category[12]

(1) Design Objective: Vertical Integration on Single-Slice Task. The recently proposed Spatial Mux-seq technology, which successfully integrates five omics modalities with spatial coordinates on a single slice. Effectively addressing the challenges of deep integration and noise correction in such datasets is essential for unlocking their full biological potential.

(2) Expanding Potential: Cross-Slice Task. Although our current study focuses on vertical integration, the underlying SpatialCOC framework has the potential to handle unpaired data (**Rebuttal Fig. 24**). Its core component—implicit neural representations—can effectively construct a continuous space and achieve spatial alignment across different tissue sections.

Extended Scenario: Cross-Slice Task

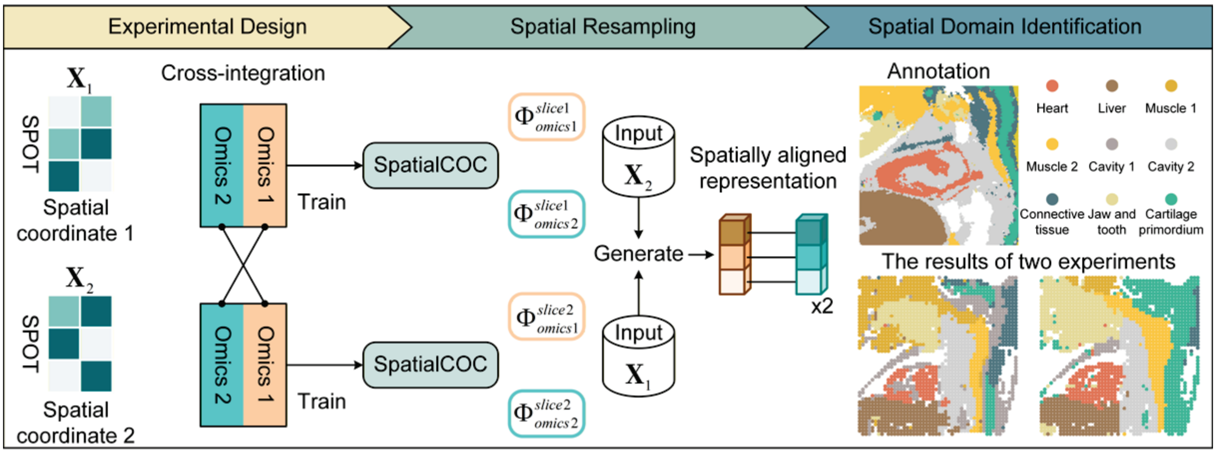

Rebuttal Fig. 24 | Extended Scenario: Cross-Slice. Cross-integration of H3K27ac and H3K27me3 profiles via spatial resampling enables spatial domain identification and performance evaluation.

We extended SpatialCOC to address this Cross-Slice scenario using E13 mouse embryo data (Spatial-Mux-seq)[9]:

- **Experimental Design.** We performed cross-integration using two biological replicates, each with H3K27me3 and H3K27ac data: 1) Integrate H3K27ac (Section 1) with H3K27me3 (Section 2); 2) The reverse combination.
- **Spatial Resampling Strategy.** SpatialCOC learned the continuous distribution of each section. We then resampled one section's omics data onto the other's spatial coordinates, enabling cross-slide integration by leveraging

shared biological patterns in replicates.

- **Downstream Analysis Verification.** The spatially-aligned omics expressions generated from the two experimental groups were integrated using SpatialCOC and subsequently subjected to spatial domain identification.

As shown in **Rebuttal Fig. 24**:

- Both experiments accurately identified major anatomical regions in the tissue sections—including the Heart, Liver, and Muscle.
- Although the two experiments were performed on different tissue sections, they showed strong consistency in identifying these major regions.

These results confirm that SpatialCOC effectively addresses cross-slice scenarios. By capturing continuous omics features within tissues, it achieves robust cross-slice generalization. This capability arises from the model's use of biological replicates from the same tissue type, which inherently share fundamental biological patterns in their feature profiles.

In summary, while SpatialCOC was originally designed for vertical integration on single-slice tasks, its underlying architecture demonstrates an inherent potential for cross-slice integration, as supported by the previous experiments. We have detailed this extension in the discussion section and plan to further enhance these capabilities in future versions.

The relevant content is now stated in the following sections of the manuscript:

- Section "Discussion" (Lines 395-400, 421-425).

2. The paper claims robustness across datasets, but the model is trained separately on each dataset. There's no attempt at cross-tissue or cross-modality generalization. No few-shot or transfer learning experiment is conducted. So how would this work if I have only limited labeled multimodal data in a new tissue?

Response: Thank you for your valuable comment regarding the learning strategy. Our description of robustness may have been unclear. We clarify that robustness here is quantified by applying the model independently to different slices from the same dataset and then measuring the consistency of the results. Accordingly, SpatialCOC is trained separately on each individual dataset; no cross-slice or cross-modal generalization is involved.

However, the few-shot learning strategy you proposed has been highly enlightening to us. It plays a crucial role in spatial multi-omics: we have observed that as the number of measurement modalities increases, the proportion of high-quality data points decreases while the proportion of noisy data points increases. This highlights the challenge of leveraging limited high-quality data for comprehensive analysis.

To explore this, we tested SpatialCOC on a tissue sample profiled by Spatial Mux-seq, demonstrating the potential utility of our strategy (**Rebuttal Fig. 25**):

- We first computed and visualized the average RNA expression per spot. Notably, the overall values of some rows and columns are relatively low—likely attributable to uneven sequencing depth or capture bias.
- We screened all rows/columns for outliers with the median absolute deviation (MAD) rule[13], flagging as "low-quality" any spot whose expression fell below a threshold of 1.5 × MAD.
- After removing these unreliable measurements, we trained the SCM module of SpatialCOC on the remaining spots to learn a continuous representation.
- The learned representation was then used to generate a global expression pattern, which can be utilized for downstream analyses.

Extended Scenario: Few-Shot Task

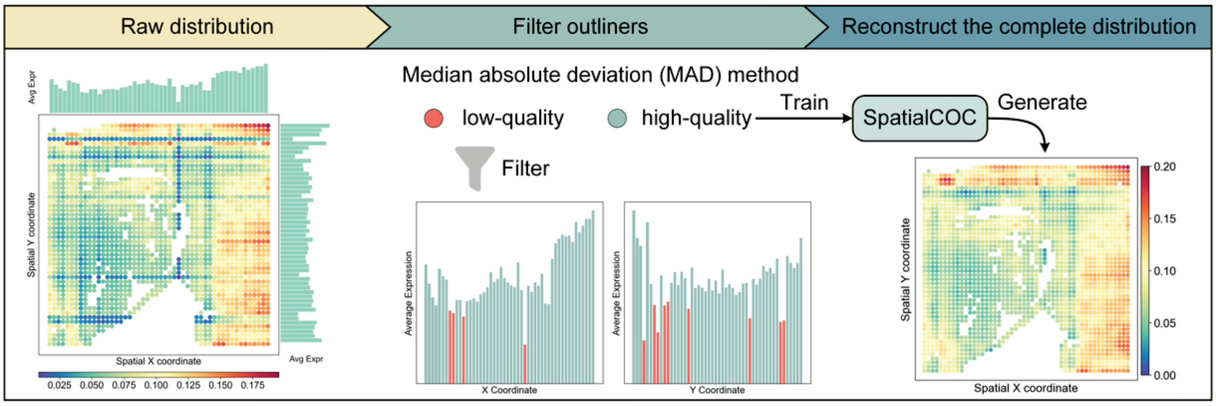

Rebuttal Fig. 25 | Extended Scenario: Few-Shot (only a subset of high-quality sample points was available).

As shown in **Rebuttal Fig. 25**, SCM module can generate a robust, globally continuous representation from only sparse, high-fidelity observations. Consequently, when only a handful of high-quality multimodal spots are available, the trained INR can synthesize a complete omics landscape, ready for downstream analysis without additional imputation steps. For demonstration purposes, the MAD-based outlier flagging can be swapped for routine cell filtering steps without altering the pipeline.

In summary, although SpatialCOC does not explicitly address cross-tissue or cross-modality generalization, its generative capabilities demonstrate strong potential for extension into few-shot learning paradigms. We anticipate incorporating this functionality in future versions.

3. The manuscript never says how the data was partitioned into training, validation, or test sets. Was there a holdout? Was generalization assessed across slices? Without this, it's really hard to know whether the results reflect general patterns or overfitting.

Response: Thank you for your valuable comment regarding the dataset division. We did not explicitly state the data partitioning scheme, which may have caused confusion, and we apologize for this oversight.

The training process is conducted in a fully unsupervised manner (**Rebuttal Fig. 26**)—meaning that no splitting of the dataset into training, validation, and test sets is required.

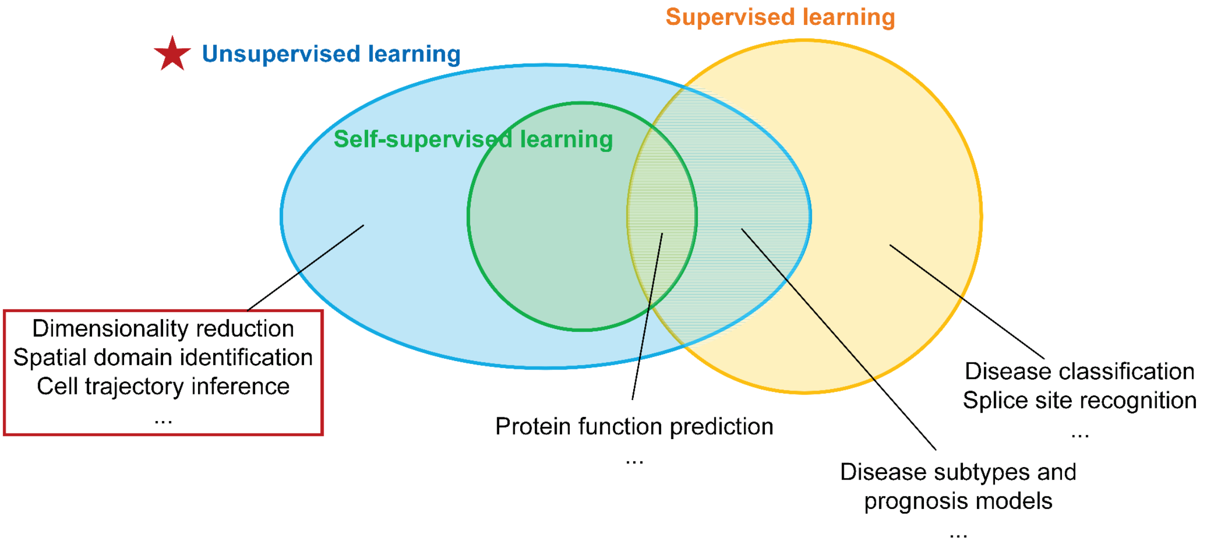

Rebuttal Fig. 26 | Description of the Training Strategy. Explanation of the relationships among unsupervised, supervised, and self-supervised learning. An unsupervised analytical framework is adopted, upon which downstream tasks—including spatial domain identification and trajectory inference—are performed.

Specifically, for each slice, we perform unsupervised training of SpatialCOC on the observed multi-omics data via the following steps (**Rebuttal Fig. 27**):

- Data preparation: Pre-process each omics layer separately (filtering, normalization, etc.) and then align the modalities using spatial information.
- Training of the deep learning framework: An integrated low-dimensional embedding is obtained by optimizing the training loss function described in

Equation (16)-(20) in the main text.

- Downstream analysis: The resulting embedding is used for downstream tasks such as spatial domain identification and trajectory inference.

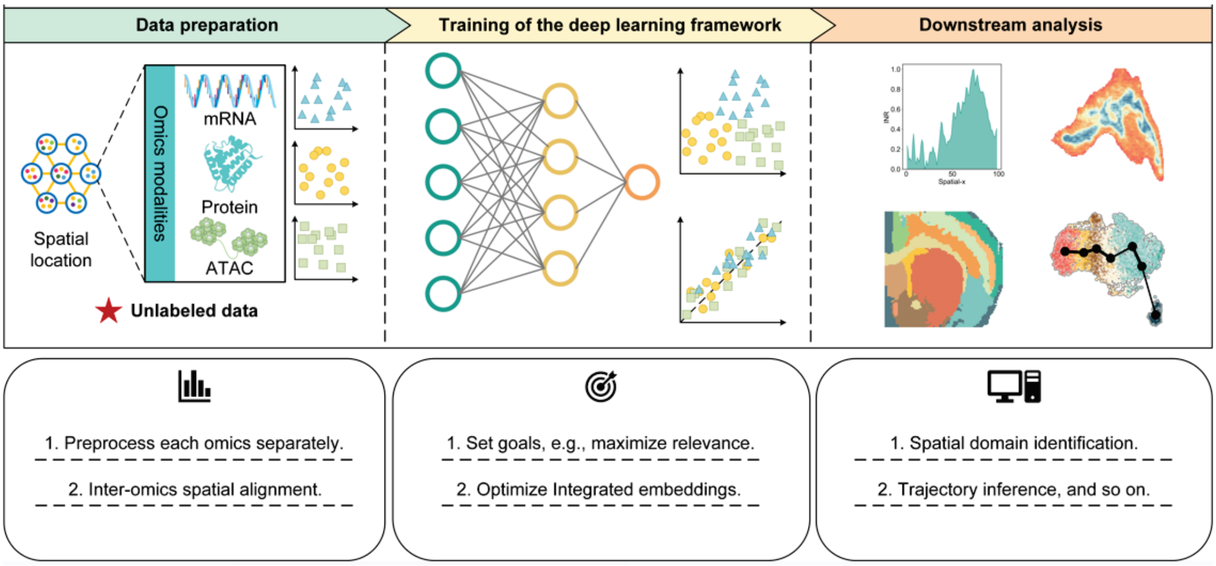

Rebuttal Fig. 27 | Unsupervised Learning Integration Framework.

The relevant content is now stated in the following sections of the manuscript:

- Section "Methods", subsection "Model training of SpatialCOC" (Lines 701-707).
- Supplementary Figure S7. Framework for Unsupervised Learning Integration in SpatialCOC (Line 569).

4. Is L_{INR} optimized together with the joint Loss of stage 2? If not, how do you select the model eventually? How early stopping or best model checkpoints are chosen in stage 1, whether INR and DCCAE are cross-validated together or independently, whether downstream performance (e.g., ARI or Moran's I) is used for model selection. The Human Lymph Node dataset is said to be "spatially allocated based on true labels" (p.16), but how exactly is this mapping done? Is there spatial autocorrelation in the raw data, or was it imposed by the simulation?

Response: Thank you for your valuable comment regarding the methodological details.

(1) Model Training Strategy

The training process is divided into two stages: first, mono-modality representation integrates individual omics data along with their spatial information; then, multimodal integration combines multiple omics modalities. This separation is due to the significant difference in training objectives between the two stages. Joint training could compromise interpretability. By examining the spatial distribution of individual omics outputs from Stage 1, we can assess whether the training is proceeding as expected.

(2) Early Stopping Strategy in Stage 1

SpatialCOC supports both raw multi-omics data and their PCA-reduced embeddings as input. As a result, there is no single criterion for early stopping. However, we have provided recommended training epochs for each dataset in our open-source tutorials. By monitoring the convergence trend of the loss curve, users can determine a suitable range for early stopping.

(3) Validation Strategy for the Two Modules

We primarily employ a cross-validated approach for validation because:

- The final integrated embeddings, which incorporate both spatial and inter-omics relationships, are ultimately used for downstream analysis.
- Although the two modules are trained separately, they are logically sequential and interdependent (see response to **Comment #1 from Reviewer #1** for details).

The rationale for this cross-validation strategy is to evaluate the contribution of each module using complementary types of metrics. For module 1, we can directly assess its output or downstream spatial metrics (such as Moran's I or CHAOS). For module 2, while ensuring the preservation of spatial information, we employ clustering accuracy metrics (e.g., ARI, AMI, NMI) for validation.

Furthermore, our ablation studies independently demonstrate the individual contributions of each module and the benefits brought by the proposed loss functions (**Rebuttal Fig. 28**).

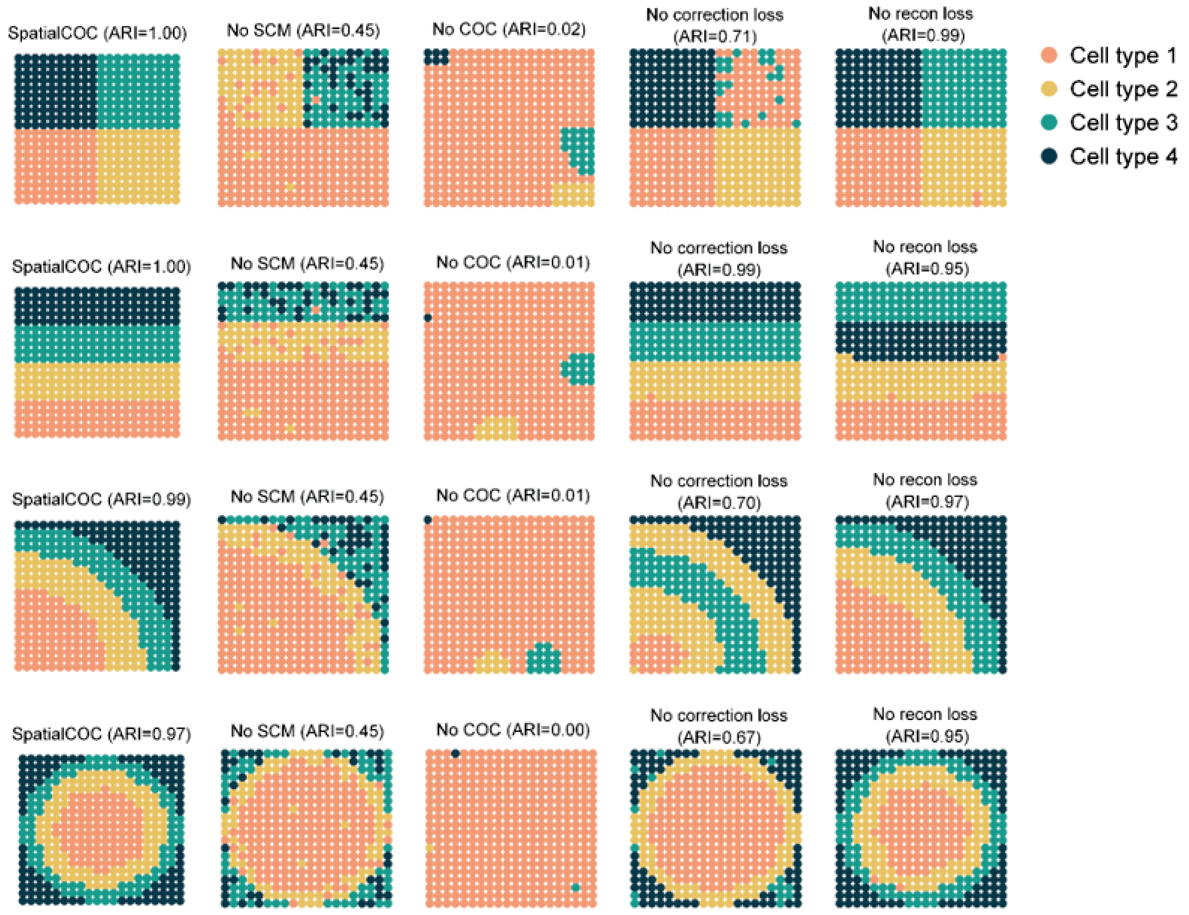

Rebuttal Fig. 28 | Ablation Experiments. Two modules were removed separately, as well as two loss functions used in the integrated training. The final spatial domain identification results were compared.

(4) Usage of Downstream Metrics

Downstream performance metrics are not used during the model training phase. We did not incorporate metrics for optimization for model training or selection. However,

these metrics can inform the evaluation of hyperparameter choices.

(5) Spatial Information Mapping of the Human Lymph Node (HLN) Dataset

- Selecting the four most abundant cell types from the HLN dataset.
- Simulating four distinct spatial patterns: quadrant, stripe, arc, and layered.
- Mapping each cell type to a distinct spatial region.

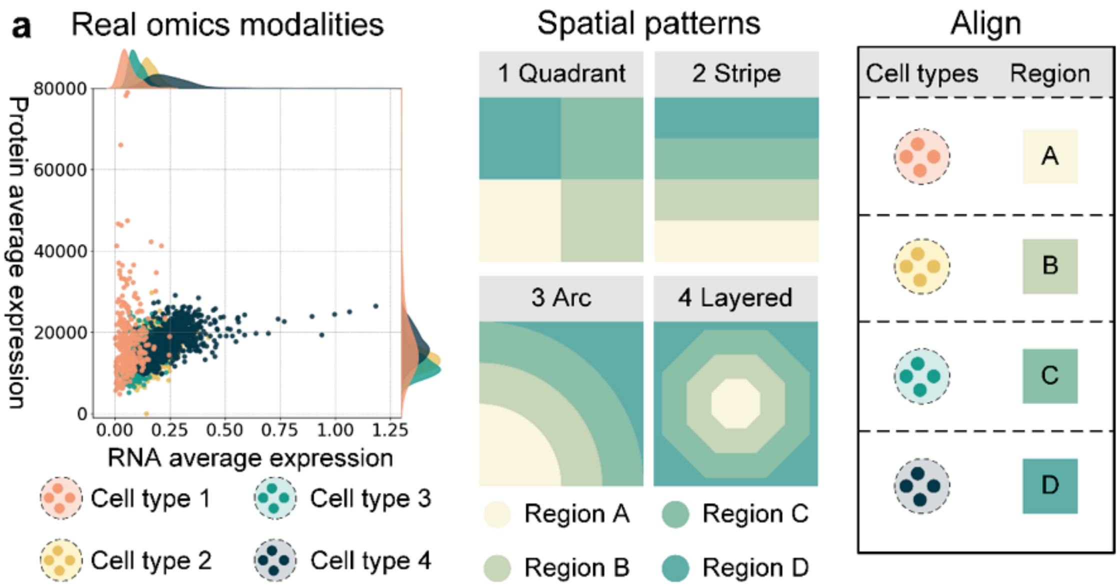

Rebuttal Fig. 29 | Simulation of Four Spatial Distribution Patterns. Four spatial distributions of increasing complexity are simulated: quadrant, stripe, arc, and layered. Real omics expressions and labels from the Human Lymph Node dataset are assigned to the simulated spatial regions.

The simulation process was Inspired by the work of Yang, P. et al. [14], which we have enhanced and improved upon.

The relevant content is now stated in the following sections of the manuscript:

(1) Model Training Strategy

- Section "Methods", subsection "Model training of SpatialCOC" (Lines 708-715).

(2) Early Stopping Strategy

- Section "Code availability" (Lines 855-858).

(3) Validation Strategy for the Two Modules

- Extended Data Fig. 1b Ablation Experiments (Line 954).
- Supplementary Figure S3. Inter-Module Logic Verification (Line 510).

5. Gaussian and pepper noise are used to simulate technical variability (p.16), but are these really representative of spatial omics noise? Dropouts, autofluorescence, segmentation artifacts—those aren't discussed. Also, there's no detail on how noise levels were adjusted. Were there SNR thresholds?

Response: Thank you for your valuable comment regarding the noise simulation. Our original description may have been unclear, we clarify that the detailed methodology for noise simulation, was already provided in Supplementary Note 3.

(1) Noise Type Selection

We model noise that reflects realistic conditions as closely as possible. Spatial multi-omics technologies frequently face a trade-off: optimizing the capture of one modality can compromise the molecular integrity required for another. Furthermore, some modalities are susceptible to "dropout" events, which reduce sensitivity in subsequent sequencing or imaging rounds.

Thus, we introduce three types of noise to the real datasets: Gaussian noise, pepper noise ("dropout" events), and mixed noise (combination of Gaussian and pepper noise).

- Gaussian Noise Simulation. To simulate measurement errors and random fluctuations in real biological signals.
- Pepper Noise Simulation. A Boolean mask was introduced to simulate "dropout" events.
- Mixed Noise Simulation. we combine Gaussian noise and pepper noise to simulate the compound effects of measurement errors and "dropout" events in biological data.

We believe this noise modeling strategy effectively captures key aspects of real-world technical variability. Details on the noise addition procedure are in **Supplementary Note 3. Simulations**.

(2) Noise Gradient Adjustments

We established four noise combinations (pairwise Gaussian and Pepper) with three gradient levels each. The Signal-to-Noise Ratio (SNR) was computed as a diagnostic

metric to confirm that the noise intensity increased logically across levels, rather than serving as a selection criterion for the gradients themselves (**Rebuttal Table 4**).

Rebuttal Table 4 | Description of noise added to the simulated datasets of different noise conditions. Each noise combination was applied with three levels of intensity, which increased sequentially. μ_1, μ_2 denote the mean values of two modalities, respectively.

Dataset	Noise level	omics 1			SNR (dB)	omics 2			SNR (dB)
		Mean value	Standard deviation	Dropout rate		Mean value	Standard deviation	Dropout rate	
Noise condition 1	1	μ_1	0.5	N/A	9.62	μ_2	0.5	N/A	7.42
	2	μ_1	1		4.47	μ_2	1		4.42
	3	μ_1	2		-1.51	μ_2	2		-0.36
Noise condition 2	1	μ_1	0.5	N/A	9.63	N/A		0.05	12.62
	2	μ_1	1		4.46			0.1	10.65
	3	μ_1	2		-1.5			0.2	7.08
Noise condition 3	1	N/A		0.05	13.03	μ_2	0.5	N/A	7.39
	2			0.1	10.01	μ_2	1		4.38
	3			0.2	7.01	μ_2	2		-0.37
Noise condition 4	1	N/A		0.05	13	N/A		0.05	13.69
	2			0.1	10			0.1	10.14
	3			0.2	7.01			0.2	7.38

We have included the noise addition in the Supplementary Information as follows:

(1) For Noise Type Selection:

- Supplementary Note. 6 Simulations (Lines 358-433).

(2) For Noise Gradient Adjustments:

- Supplementary Note. 4 Evaluation Metrics (Lines 142-151).
- Supplementary Table S1 (Line 580).

6. Fig. 3a The reciprocal gradient method used here is unconventional. Though later Moran score makes more sense, the current choice feels a bit ad hoc.

Response: Thank you for your valuable comment regarding the quantification of spatial continuity. We fully agree that method evaluation should be based on rigorous metrics. The Moran's I score quantifies spatial autocorrelation, capturing both global spatial patterns and local smoothness. Therefore, we supplemented our analysis by calculating Moran's I score as follows:

- Original and INR-reconstructed expression data were obtained from four mouse brain slices.
- For each spot, we computed the average expression across both omics modalities for both the original and reconstructed data.
- The Moran's I score was calculated using the `sq.gr.spatial_autocorr()` function from the Squidpy package.

The results are reported in **Rebuttal Table 5**:

Rebuttal Table 5. Moran's I socre of two omics and their INR.

Slice	Transcriptome	INR of Transcriptome	Epigenome	INR of Epigenome
ATAC	0.572646	0.941615	0.374078	0.805174
H3K4me3	0.415429	0.906844	0.325253	0.788599
H3K27ac	0.433379	0.912711	0.267781	0.764607
H3K27me3	0.540626	0.93443	0.305331	0.681131

We have replaced the previous smoothness score with Moran's I score. This change has been applied in Fig. 3 (Line 898) and in the main text (Lines 250-255).

7. Fig. 3c The dual-metric plot is great, but without error bars across slices, it's hard to tell how reproducible these metrics are.

Response: Thank you for your positive comment on our metric visualization. We fully agree that reproducible quantitative metrics are crucial for the comparative evaluation of computational methods. The calculation of these metrics is uniquely determined by the clustering results (i.e., the cluster label assigned to each spot). By fixing the random seed, the metric values remain consistent across runs, provided the clustering results are unchanged.

To enhance the rigor of our visualization, we have added significance tests for differences between experimental groups. Using paired t-test method, we evaluated the statistical significance for each of the dual metrics separately. We have added the results of inter-group significance testing to Fig. 3c (**Rebuttal Fig. 30**). This dual-metric analysis demonstrates that our method achieves an excellent balance between spatial continuity and biological relevance, significantly outperforming existing state-of-the-art methods.

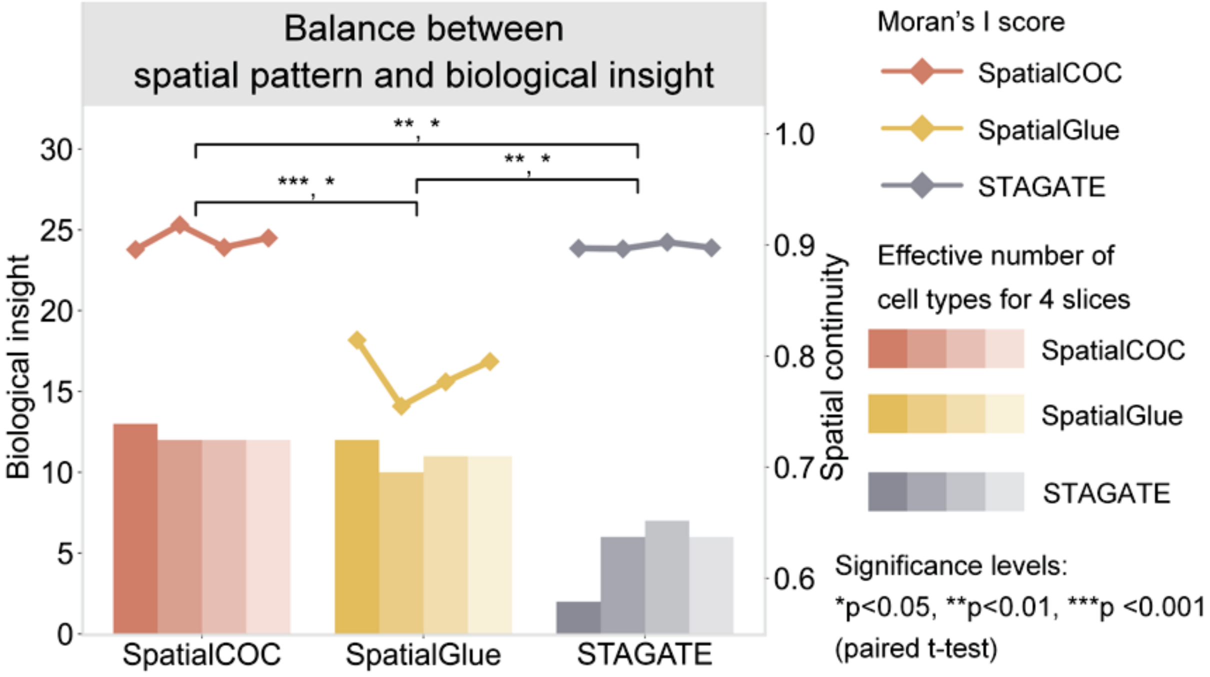

Rebuttal Fig. 30 | Quantification of Spatial Smoothness and Biological Insight.

The detailed results of the paired t-test are presented in **Rebuttal Table 6**.

Rebuttal Table 6. Dual-Metrics Significance Test.

	Spatial Continuity	Biological Insight
SpatialCOC vs SpatialGlue	t = 25.000, p = 0.0001***	t = 5.000, p = 0.0154*
SpatialCOC vs STAGATE	t = 12.358, p = 0.0011 **	t = 5.170, p = 0.0140*
SpatialGlue vs STAGATE	t = 9.258, p = 0.0027 **	t = 4.004, p = 0.0279*

8. Fig 4 The PAGA trajectories look compelling, but the parameters used for inference aren't reported. PAGA is sensitive to clustering resolution and graph construction—how were these chosen?

Response: Thank you for your valuable comment regarding the details of the PAGA method. We fully acknowledge that the PAGA method is sensitive to parameter settings. Accordingly, we have moved the detailed parameters and implementation steps of PAGA from the supplementary information to the Methods section:

After obtaining the integrated multimodal embeddings, we performed the trajectory inference task using the PAGA method. We first constructed the nearest neighbor graph using the `sc.pp.neighbors()` with *n_neighbors=50* (a parameter chosen for this dataset to ensure a fair and consistent comparison across methods). Subsequently, we performed PAGA analysis using the `sc.tl.paga()` with default parameters.

To ensure transparency and reproducibility of our PAGA analysis, we have also provided the relevant execution code (The code below is open-source and can be accessed as mentioned in the Materials availability section of the main text):

```
sc.pp.neighbors(
adata,
use_rep=embed_key,
key_added=f'neighbors_{cluster_key}',
n_neighbors=50
)
sc.tl.paga(
adata,
groups=cluster_key,
neighbors_key=f'neighbors_{cluster_key}'
)
adata.uns[f'paga_{cluster_key}'] = adata.uns['paga'].l.copy()
```

9. Fig. 4a Claims about identifying cortex, medulla, and connective tissue aren't supported by marker gene expression or histological overlays. A simple validation would make these claims stronger.

Response: Thank you for your comment regarding the multimodal biomarker analyses. We fully agree that a comprehensive interpretation of marker genes significantly enhances the credibility and biological relevance of our findings.

In response, we have made the following additions to the manuscript:

- Extended Data Fig. 9 Validation of Marker Genes and Proteins Across Three Replicates of the Mouse Thymus Dataset (Line 1125).
- We have added a new section in the Supplementary Information, which provides a detailed analysis of the marker genes for each dataset (Supplementary Note 5, "Supplementary Analyses of Multimodal Biomarkers" Lines 223-356).

10. The batch effects in Fig. 4d are shown visually, but no quantitative metric is used. Could the authors add something like batch entropy or LISI to objectively assess batch mixing?

Response: Thank you for your valuable comment regarding the quantification of batch effects. We fully agree that assessing batch effect removal requires robust quantitative evidence.

Quantitative metrics for batch effect removal are primarily categorized into two types. The first relies on a shared embedding space to evaluate batch mixing, such as the Local Inverse Simpson's Index (LISI[8]), which quantifies label heterogeneity in local cell neighborhoods. The second assesses biological structural consistency without a shared embedding, as exemplified by SPIRAL[11], which employs Gromov-Wasserstein distance to measure the alignment of spatial or topological structures. We apply this second type of metric to evaluate the batch consistency of PAGA graph analysis results obtained from different methods.

Specifically, we quantified batch effects by calculating the cosine similarity between node-aligned PAGA graphs constructed from different biological replicates. This was achieved by searching through all possible node permutations to identify the best alignment between graphs from different replicates. A smaller distance indicates better reproducibility of biological structures across samples, reflecting more effective removal of batch effects.

We employed the Frobenius norm as our distance metric between adjacency matrices. For two graphs represented by adjacency matrices $\boldsymbol{A}$ and $\boldsymbol{B}$ of size $n \times n$, the graph distance under a permutation π is defined as:

$$D_\pi(\boldsymbol{A}, \boldsymbol{B}) = \| \boldsymbol{A} - \boldsymbol{P}_\pi^T \boldsymbol{B} \boldsymbol{P}_\pi \|_F$$

where:

- $\boldsymbol{P}_\pi$ is the permutation matrix corresponding to permutation π;
- $\| \cdot \|_F$ denotes the Frobenius norm.

Then we searched all possible node permutations to find the best alignment between graphs from different replicates:

$$D_{min}(\boldsymbol{A},\boldsymbol{B}) = \min_{\pi \in S_n} D_{\pi}(\boldsymbol{A},\boldsymbol{B})$$

where S_n is the symmetric group of all permutations on n elements. This optimal matching accounts for potential label switching between replicates.

Unfold the upper triangular parts of the aligned matrices $\boldsymbol{A}$ and $\boldsymbol{B}$ into two one-dimensional vectors $\boldsymbol{a}$ and $\boldsymbol{b}$, and then calculate their cosine similarity: $similarity = \frac{\boldsymbol{a} \cdot \boldsymbol{b}}{\|\boldsymbol{a}\| \cdot \|\boldsymbol{b}\|}$ to quantify the batch effects.

Higher similarity scores in this comparison indicate better performance, as they reflect superior reproducibility and reduced batch effects across replicates (**Rebuttal Table 7**).

Rebuttal Table 7. Cosine Similarity Between PAGA Graphs

	**SpatialCOC**	**SpatialGlue**	**Seurat**	**STAGATE**
Rep1 vs Rep2	**0.967985**	0.936008	0.819309	0.963164
Rep1 vs Rep3	**0.992636**	0.874468	0.849186	0.943693
Rep2 vs Rep3	**0.96498**	0.84534	0.925135	0.919335

Rebuttal Table 7 presents a quantitative assessment of batch effect removal. The results show that our method consistently achieves higher cosine similarities (indicating better performance) in all pairwise comparisons. We have also incorporated a visualization of this metric (**Rebuttal Fig. 31**).

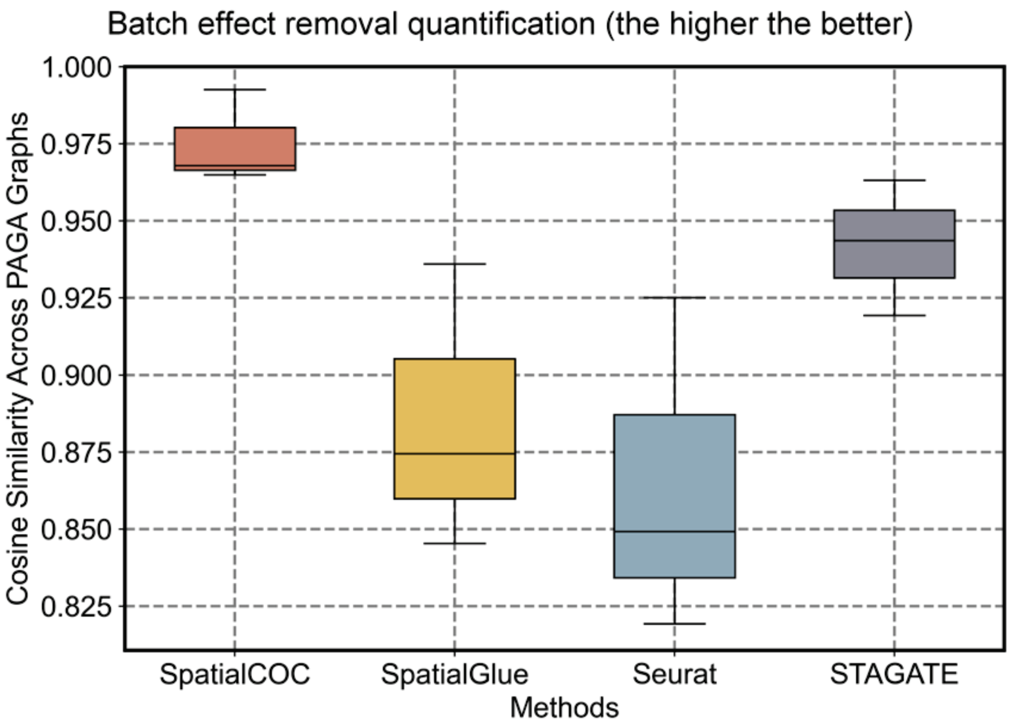

Rebuttal Fig. 31 | Batch Effects Removal Quantification (The Higher the Better)

The relevant content is now stated in the following sections of the manuscript:

- Fig. 4f Quantitative Measurement of Batch Effect Removal (Line 924).
- Supplementary Note.4 Evaluation Metrics (Lines 203-221).

11. The Gaussian kernel used in INR has σ fixed to 0.02—but why? Was this tuned? Did they test sensitivity? The spatial kernel is critical to performance and really should be better justified.

Response: Thank you for your valuable comment regarding the parameter setting of σ. This comment is closely relevant to **Comment #2 from Reviewer #1**. We have thoroughly discussed it and provided experimental verification results.

(1) Parameter Design

To capture the spatial patterns of omics data while maintaining spatial continuity, we introduced two key parameters in module 1:

- ω_0 (Frequency Parameter): Used in Equation (5), this parameter controls the frequency of the sinusoidal activation function, influencing the model's ability to capture global spatial patterns while maintaining continuity.
- σ (Kernel Bandwidth): Used in Equation (16), this is the bandwidth parameter of the Gaussian kernel, which enhances local spatial continuity.

(2) Systematic Testing of ω_0

From the perspective of Fourier analysis, a multi-layer composite structure with sine activation functions of appropriate frequencies can effectively approximate signals containing different frequency components (low frequencies: smooth regions; high frequencies: boundaries). Therefore, as long as ω_0 remains within a certain range, this structure can simultaneously capture both smooth regions and boundary features in the signal.

Using the Mouse Brain dataset as a representative example, we conducted a comparative analysis of the reconstruction performance achieved by sinusoidal activation functions across different frequency settings. The linear ReLU activation function served as the benchmark in this assessment (**Rebuttal Fig. 32**).

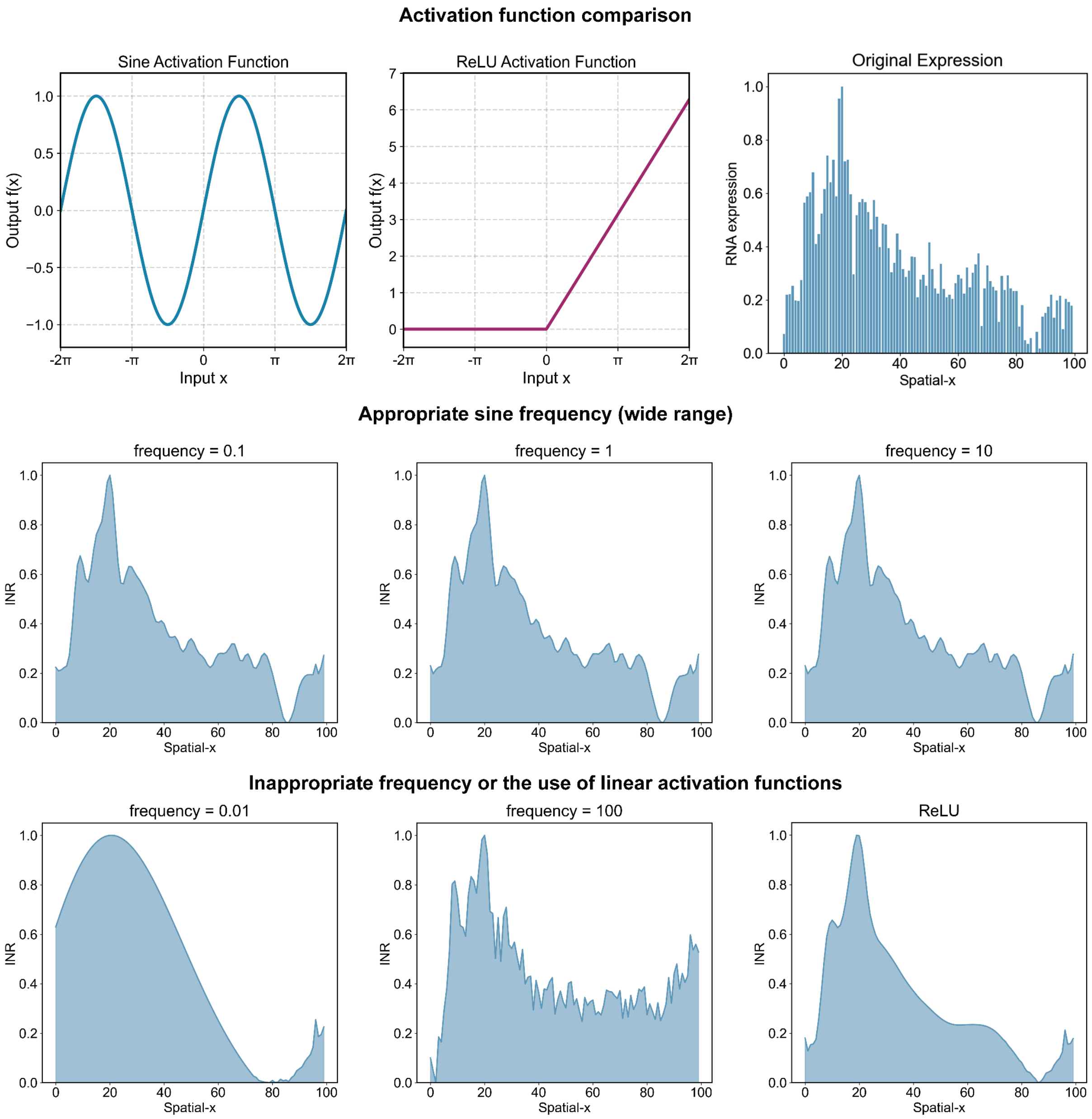

Rebuttal Fig. 32 | Comparison of Different Activation Functions Used in SCM Module. We compared the reconstruction performance of sinusoidal activation functions across different frequencies, using the ReLU (linear-type) activation as a benchmark.

As shown in **Rebuttal Fig. 32**:

- The sinusoidal activation function effectively captures both smooth regions and boundary information, outperforming the linear ReLU activation.
- Excessively low ω_0 values fail to capture edge signals, while overly high values cause overfitting to boundary details.

- Performance remains highly stable within the range [0.1, 10], making parameter selection straightforward.

We have included this analysis, along with a mathematical explanation, in the Supplementary Information, as also detailed in our response to **Comment #2 from Reviewer #1**.

(3) Systematic Testing of σ

This parameter (σ) serves as a smoothness regularizer in SpatialCOC. A larger value enhances the spatial smoothing effect, while a smaller value reduces it. Accordingly, we have set its default to a relatively small value of 0.02, which provides only limited local smoothing. We tested this setting across all datasets and observed consistently strong performance.

We supplemented the comparative experiments (removing Kernel and adjusting ω_0):

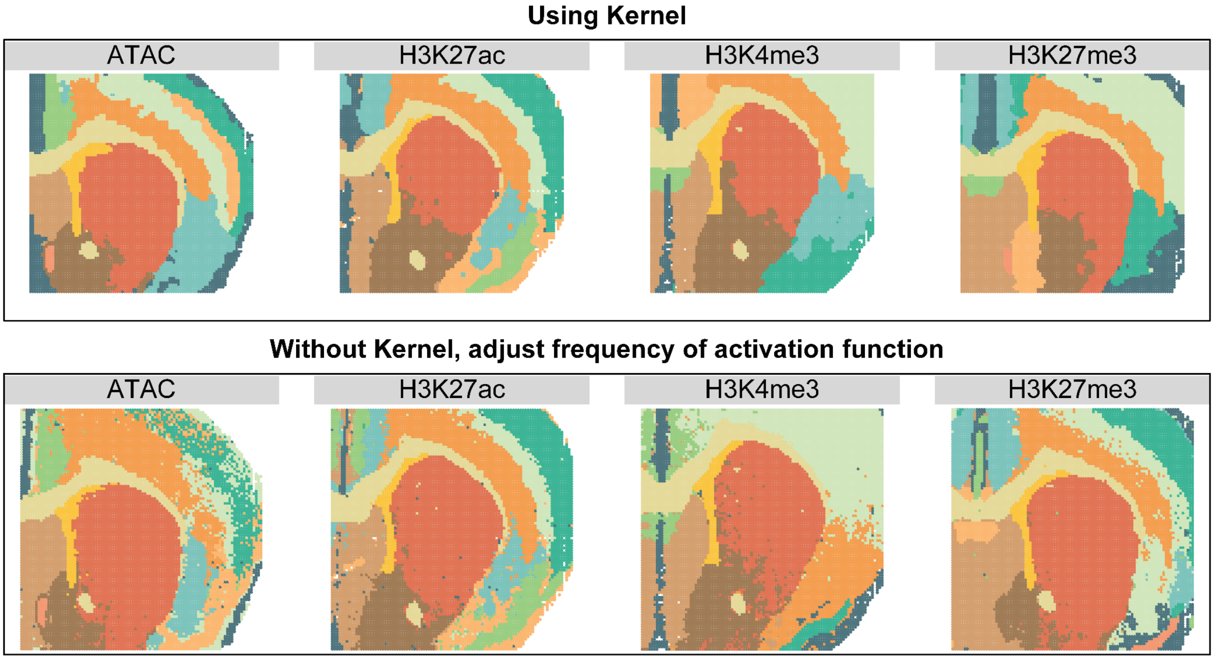

Rebuttal Fig. 33 | Effect of Kernel Removal. We compared the performance on the mouse brain dataset with and without the kernel.

As shown in **Rebuttal Fig. 33**, removal of the kernel maintains overall result stability, with only a reduction in local smoothness. Following your suggestion, we have made it an optional parameter. Given the inherent trade-off between smoothness and

boundary definition, users can now choose to enable or disable this kernel based on their specific needs.

In summary, we have conducted a supplementary analysis of two critical parameters in SpatialCOC—the frequency parameter $\boldsymbol{\omega}_0$ and the kernel bandwidth σ—both of which are significant for capturing spatial information. Our analysis provides deeper insights into their roles, which ultimately assists users in selecting appropriate parameter values for their own practical analyses.

The relevant content is now stated in the following sections of the manuscript:

- Supplementary Note 1. Supplementary Details of the SCM Module (Lines 20-89).
- Supplementary Figure S1. Comparison of Different Activation Functions Used in SCM Module (Line 492).
- Supplementary Figure S2. Effect of Kernel Removal (Line 501).

12. The benchmark mostly focuses on datasets with prominent tissue structures. What about rare cell types or smooth transitions between states?

Response: Thank you for the valuable comment regarding the scope of our benchmark datasets. A robust evaluation of integrative methods must account for the inherent spatial structural heterogeneity in real tissues.

We have categorized and summarized the complex spatial patterns in spatial multi-omics. Our simulated spatial patterns and the selection of real-tissue samples are highly diverse, comprehensively covering major patterns such as smooth gradients, sharp boundaries, hierarchical structures, and complex regions (**Rebuttal Fig. 34**).

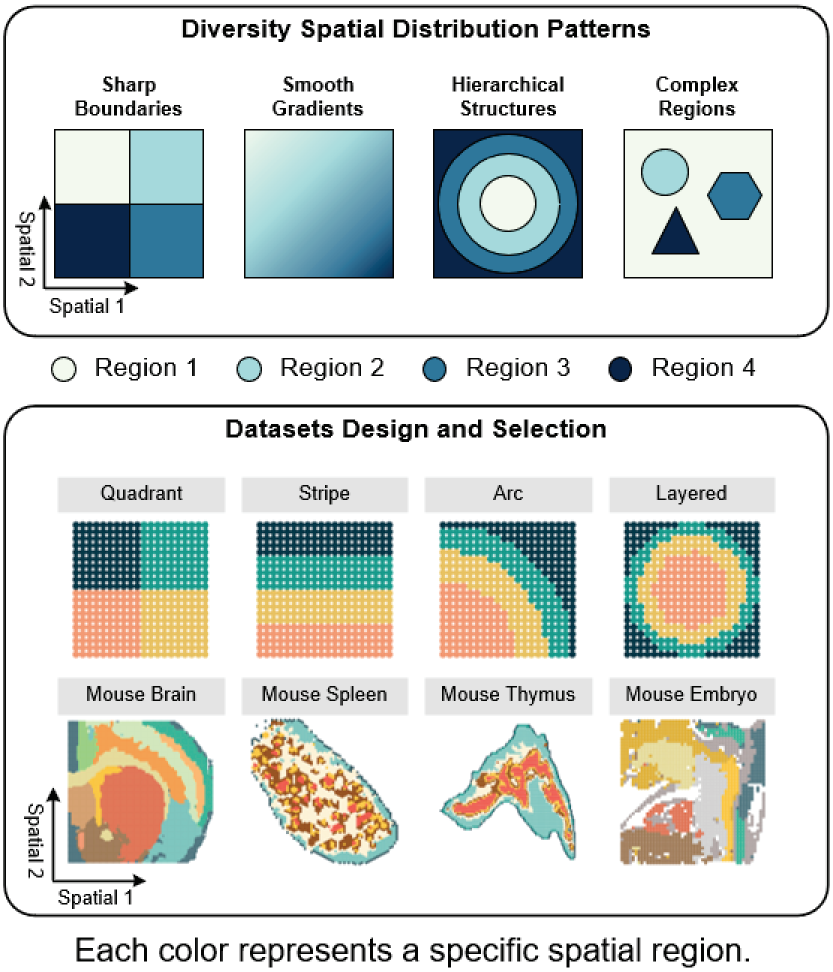

Rebuttal Fig. 34 | Real Spatial Patterns and Datasets Used For Evaluation. For both virtual data simulation and real-world data selection, we took into account the following diverse spatial distribution characteristics: smooth gradients, sharp boundaries, hierarchical structures, and complex regions.

As shown in **Rebuttal Fig. 34**:

- **Smooth Gradients:** Cell types or gene expression can form smooth continuous gradients within tissues, for instance, the gradual changes in gene expression observed across the cerebral cortex[4].
- **Sharp Boundaries:** At the interfaces between tissue domains, abrupt transitions in cellular composition or gene expression often occur, forming sharp anatomical boundaries, such as the demarcation between the hippocampus and the cortex[1].
- **Hierarchical Structures:** Within tissues and organs, such as pancreatic germinal centers[5] or thymic lobules, the spatial arrangement of cells often exhibits clear hierarchical or zonal organization.
- **Complex Regions:** Examples include organ primordia during mouse embryonic development, such as the developing liver and heart in the embryo[6].

In the simulated dataset, the spatial structures of various real tissues have been simulated except for the complex regions. Moreover, the selected real datasets all cover the following types of spatial structures:

- **Mouse Brain Dataset with Mixed Smooth and Boundary Regions.** The spatial structure of this dataset demonstrates two typical characteristics - it contains both continuous regions with smooth transitions in cell states (e.g., in cortical areas) and regions with clear tissue boundaries (e.g., the hippocampal-cortical junction).
- **Mouse Spleen Dataset with a Central–Peripheral Pattern.** Through combined histopathological image analysis, we found that this dataset exhibits a typical central-peripheral distribution pattern, containing both central cell aggregation areas (showing non-smooth distribution) and peripheral smooth transition regions.
- **Mouse Thymus Dataset with a Hierarchical Structure.** This dataset displays a distinct spatial hierarchy, enabling clear differentiation between the

central medullary area and peripheral cortical region. Particularly noteworthy is that SpatialCOC successfully identified the connective tissue transition zone in the central area, demonstrating its capability to capture subtle structural variations.

- **Mouse Embryo Dataset with Complex Tissue Architecture.** This dataset serves to evaluate the cross-slice integration capability of SpatialCOC, as it clearly reveals major anatomical structures like the heart, liver, and muscle. Results demonstrate that SpatialCOC accurately identifies these large-scale structures in complex developing tissues, confirming its effectiveness in cross-slice analysis.

In summary, the selected datasets comprehensively cover various spatial scenarios, including smooth gradients, sharp boundaries, hierarchical structures, and complex regions, thereby systematically validating the effectiveness and generalizability of SaptialCOC.

The relevant content is now stated in the following sections of the manuscript:

- Supplementary Note. 7 The Datasets Used For Evaluation (Lines 435-489).
- Supplementary Figure S6. The Datasets Used For Evaluation (Line 561).

Reviewer #3

I co-reviewed this manuscript with one of the reviewers who provided the listed reports. This is part of the Nature Communications initiative to facilitate training in peer review and to provide appropriate recognition for Early Career Researchers who co-review manuscripts.

Reviewer #3 (Remarks on code availability):

The code is well structured.

Response: We sincerely thank you for your valuable participation in the review process. Your perspective contributed to a more thorough and insightful review.
We also appreciate the co-reviewer initiative—it is a meaningful way to support early-career researchers while enhancing the quality and diversity of peer review.

References

1. Allen Reference Atlas – Mouse Brain Coronal Atlas. Available from atlas.brain-map.org. (Accessed: 2025-09-15).
2. Moshe Leshno, Vladimir Ya. Lin, Allan Pinkus, and Shimon Schocken. Multilayer feedforward networks with a nonpolynomial activation function can approximate any function. Neural Networks, 6(6):861–867 (1993).
3. Stade, E. Fourier Coefficients and Fourier Series. In Fourier Analysis, E. Stade (Ed.) (2005).
4. Maynard, K.R., Collado-Torres, L., Weber, L.M. *et al.* Transcriptome-scale spatial gene expression in the human dorsolateral prefrontal cortex. *Nat Neurosci* **24**, 425–436 (2021).
5. Ben-Chetrit, N., Niu, X., Swett, A.D. *et al.* Integration of whole transcriptome spatial profiling with protein markers. *Nat Biotechnol* **41**, 788–793 (2023).
6. Chen, A., Liao, S., Cheng, M. et al. Spatiotemporal transcriptomic atlas of mouse organogenesis using DNA nanoball-patterned arrays. Cell, 185(10), 1777-1792 (2022).
7. Qiu, P. Embracing the dropouts in single-cell RNA-seq analysis. *Nat Commun* **11**, 1169 (2020).
8. Korsunsky, I., Millard, N., Fan, J. *et al.* Fast, sensitive and accurate integration of single-cell data with Harmony. *Nat Methods* **16**, 1289–1296 (2019).
9. Saelens, W., Cannoodt, R., Todorov, H. et al. A comparison of single-cell trajectory inference methods. *Nat Biotechnol* **37**, 547–554 (2019).
10. Asp, M., Bergenstråhle, J. & Lundeberg, J. Spatially resolved transcriptomes-next generation tools for tissue exploration. *BioEssays* **42**, 1900221 (2020).
11. Guo, T., Yuan, Z., Pan, Y. *et al.* SPIRAL: integrating and aligning spatially resolved transcriptomics data across different experiments, conditions, and technologies. *Genome Biol* **24**, 241 (2023).
12. Hu, Y., Wan, S., Luo, Y. *et al.* Benchmarking algorithms for single-cell multi-omics prediction and integration. *Nat Methods* **21**, 2182–2194 (2024).

13. Hampel, F. R. The Influence Curve and its Role in Robust Estimation. *Journal of the American Statistical Association*, *69*(346), 383–393 (1974).
14. Yang, P., Jin, L., Liao, J. et al. Revealing spatial multimodal heterogeneity in tissues with SpaTrio. *Cell Genomics* **3**, 100446 (2023).
